# PPh_3_/NaI driven photocatalytic decarboxylative radical cascade alkylarylation reaction of 2-isocyanobiaryls†

**DOI:** 10.1039/d0ra03211e

**Published:** 2020-04-25

**Authors:** Ketan Wadekar, Suraj Aswale, Veera Reddy Yatham

**Affiliations:** Department of Organic Synthesis & Process Chemistry, CSIR-Indian Institute of Chemical Technology Hyderabad 500007 India; Academy of Scientific and Innovative Research (AcSIR) Ghaziabad 201002 India; CSIR-Indian Institute of Chemical Technology Hyderabad 500007 India; School of Chemistry, Indian Institute of Science Education and Research Thiruvananthapuram 695551 India reddy@iisertvm.ac.in reddy.iisc@gmail.com

## Abstract

The first triphenylphosphine/sodium iodide driven photocatalytic decarboxylative cascade cyclization of 2-isocyano-biaryls with alkyl *N*-hydroxyphthalimide (NHP) esters was developed. This operationally simple protocol results in multiple carbon–carbon bond formation under transition metal free conditions, affording a novel and environmentally benign entry to producing 6-alkyl phenanthridines with moderate to good yields.

## Abstract 2

PPh_3_/NaI driven photocatalytic decarboxylative cyclization of 2-isocyanobiaryls with alkyl NHP esters was developed. This simple protocol afforded a novel and environmentally friendly approach to furnish 6-alkyl phenanthridines in good yields.

## Graphical abstract

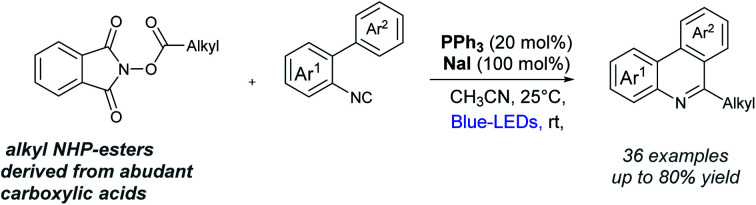

## Introduction

Visible-light photocatalysis has been widely recognized as a powerful tool in organic synthesis to construct carbon–carbon or carbon–heteroatom bonds.^1^ Through reactive intermediates such as radicals and radical ions, unique reactions that are previously inaccessible under thermal conditions can be accessed. Significant advances have been realized in this field by employing metal based catalysts^2^ or organic dyes^3*a*–*d*^ as photoredox catalysts. However, employing these metal based photoredox catalysts has certain disadvantages: such as; they are expensive, potentially toxic and limited availability.^3*e*^ Recently, visible light driven photoredox catalysis employing inexpensive chemicals such as combination of triphenylphosphine/sodium iodide and CeCl_3_ emerged as a robust alternative to generate radical entities under mild reaction conditions.^4^ This approach replaces metal based photocatalysts by inexpensive catalysts, thus overcoming some of the aforementioned problems.

Phenanthridine, a privileged structural core motif found in natural products.^5^ Many synthetic phenanthridine derivatives show bioactive and pharmaceutical properties, including anti-tumoral, antibacterial, antiviral, cytotoxic, and DNA inhibitory properties.^6,7^ In addition, phenanthridine derivatives reveal significant optoelectronic properties.^8^ Therefore, the development of new and efficient methods for the preparation of phenanthridine derivatives has gained significant importance in academic research. Recently, a cascade radical pathway involving radical addition to 2-isocyanobiaryls and subsequently intramolecular homolytic aromatic substitution has been developed,^9^ which allows the rapid assembly of a phenanthridine framework with high efficiency. In 2012, Chatani's group disclosed Mn(iii)-mediated radical cascade reaction of 2-isocyanobiaryls with boronic acid under thermal conditions.^10^ Subsequently, several groups put their efforts for the construction of 6-substituted phenanthridines through the reaction of 2-isocyanobiaryls with corresponding carbon radical precursors, such as simple alkanes,^11^ alcohols,^11*a*^ ethers,^12^ aldehydes,^13^ 2-bromide ethyl esters,^14^ aryl sulfonyl chlorides^15^ and 1,3-dicarbonyl compounds.^16^ Although these reported methods have their own specific applications, but remains associated with certain disadvantages such as employing metal catalysts, harsh reaction condition and use of stoichiometric amounts of oxidants. Very recently, few groups reported a metal-free approach that utilizes aryl amines,^17^ carboxylic acids,^18^ hydrazines^19^ acyl peroxides,^20^ as a carbon radical precursor, which appears to be an environmentally benign method for accessing 6-substituted phenanthridines. However, most of acyl peroxides, alkyl amines and hydrazenes are commercially unavailable. Thus, further new methodologies are quite desired for the synthesis of 6-substituted phenanthridines.

Recently decarboxylative functionalization of alkyl *N*-hydroxyphthalimide (NHP) esters in carbon–carbon bond formation reactions have gained a significant importance, as they convert widely available and inexpensive chemicals into valuable chemicals and reactive intermediates for synthesis.^21^ Photo decarboxylation of alkyl *N*-hydroxyphthalimide (NHP) esters has been widely used for organic synthesis,^22^ as the liberation of phthalimide and volatile CO_2_ as a by-products indicates a strong driving force for the reaction, forming versatile radical intermediates to build more valuable products. Herein, we report the first PPh_3_/NaI driven photocatalyzed decarboxylative cascade alkylarylation reaction of 2 isocyanobiaryls in the presence of visible light irradiation at room temperature.

## Results and discussions

**Fig. 1:**
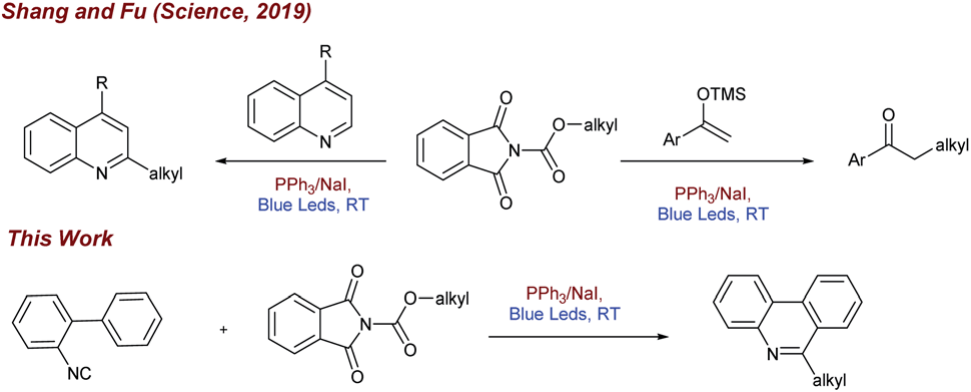
Decarboxylative alkylations driven by PPh_3_/NaI photoredox catalysis.

**Fig. 2:**
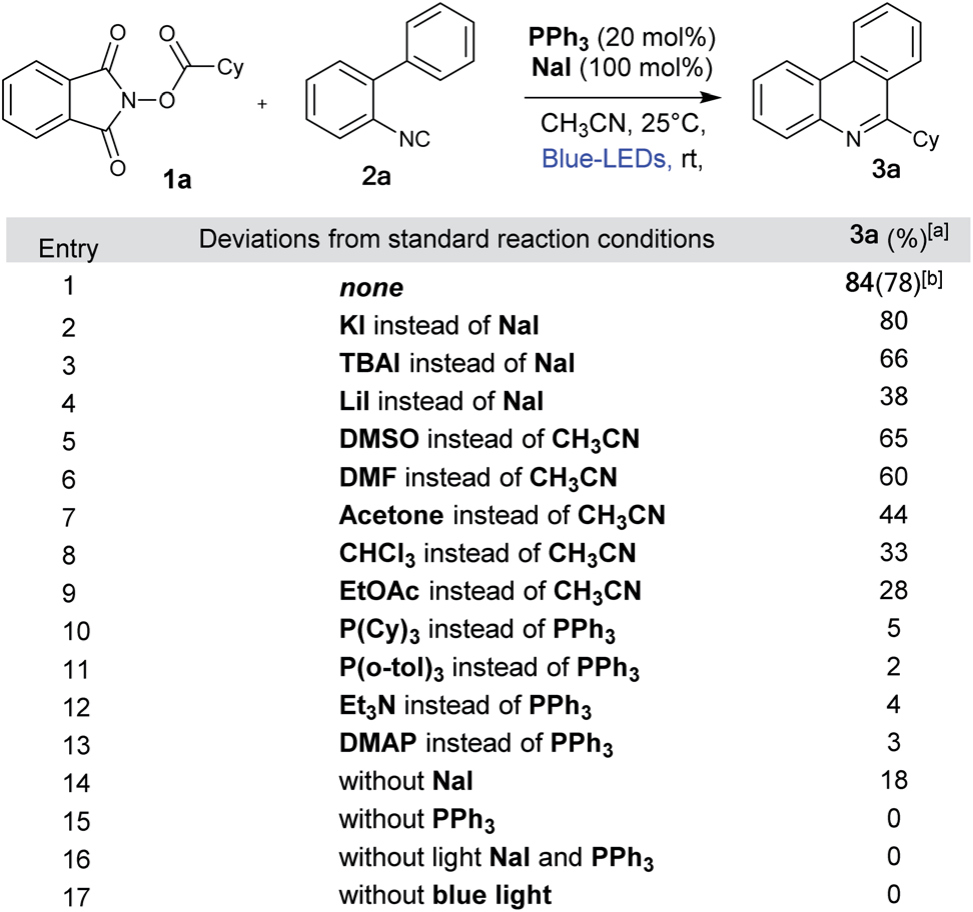
Optimization of the reaction conditions. 1a (0.3 mmol), 2a (0.2 mmol), PPh_3_ (20 mol%), NaI (100 mol%), CH_3_CN (3 mL) at 25 °C, 455 nm LED for 24 h. ^[a]^NMR yields using benzyl alcohol as internal standard. ^[b]^Isolated yield.

Inspired by the recent work of Shang and Fu (Fig. 1) who developed photocatalytic decarboxylative alkylations mediated by triphenylphosphine/sodium iodide,^4*d*^ we wondered how these activated carboxylic acids (1) would behave under similar reaction conditions (Fig. 1) in presence of 2-isocyanobiaryls (2). At first, we chose 1a and 2a as model reactants to optimize the reaction conditions for the desired decarboxylative radical cascade alkylarylation using inexpensive PPh_3_/NaI as a photocatalyst. To our delight, when a solution of 1.5 equiv. of 1a with 1.0 equiv. of 2a in the presence of PPh_3_ (20 mol%) and NaI (100 mol%) in acetonitrile was illuminated with a blue LED (455 nm) at 25 °C for 24 h, product 3a was formed in 84% yield (Fig. 2, entry 1). Replacement of NaI by KI also proceeded smoothly to give product 3a in 80% yield (Fig. 2, entry 2). The product formation 3a was slightly decreased upon employing other iodide salts (Fig. 2, entries 3 and 4). Solvents such as dimethylsulfoxide (DMSO) and dimethylformamide (DMF) in place of MeCN afforded 3a in 65% and 60% yield respectively (Fig. 2, entries 5 and 6). While solvents such as acetone, chloroform (CHCl_3_) and EtOAc had a deleterious effect on the reaction outcome (Fig. 2, entries 7–9). When triphenyl phosphine was replaced by other phosphines such as PCy_3_ and P(*o*-tol)_3_, caused a drastic reduction in the yield of the reaction (Fig. 2, entries 10 and 11). Substitution of PPh_3_ by other nitrogen donors such as DMAP, Et_3_N observed trace amount of product (Fig. 2, entries 12 and 13). Additionally, control experiments confirmed that a catalytic amount of PPh_3_ and continuous blue light irradiation were necessary for the reaction to occur. Not even traces amount of 3a were observed in the absence of light or the PPh_3_ (Fig. 2, entries 15 and 17).

**Fig. 3:**
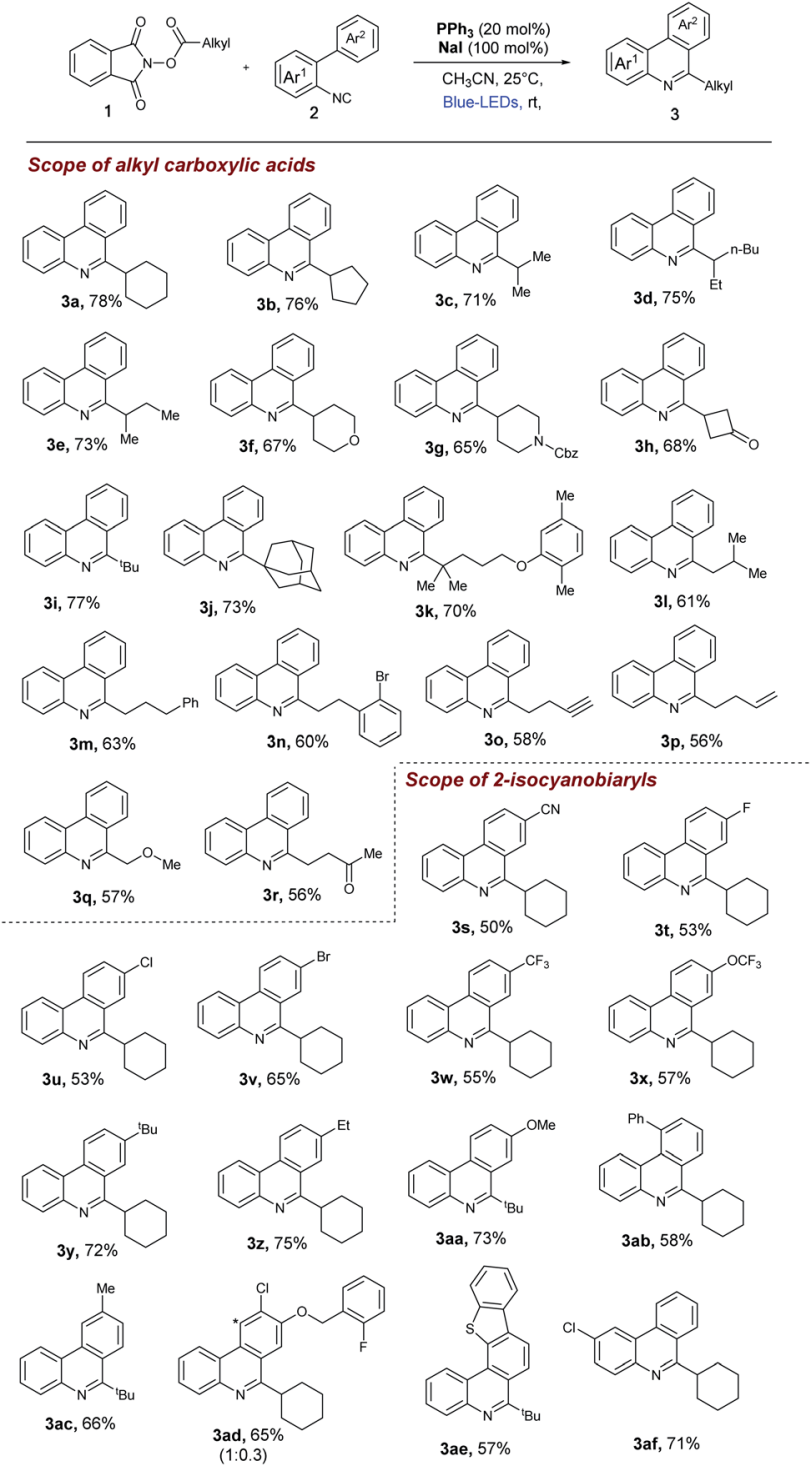
Decarboxylative cascade alkylarylation reaction of 2-isocyanobiaryls with alkyl NHP-esters. Reaction conditions as given in Fig. 2 (entry 1). Isolated yields, average of at least two independent runs. *regioisomer.

With the optimized reaction conditions in our hand, we survey the scope of the reaction. As shown in Fig. 3, a broad range of alkyl NHP esters (1), reacted with 2-isocyanobiphenyl (2a) providing the corresponding 6-alkyl phenanthridines in good to moderate yields. Different types of secondary cyclic (1a–1b) and acyclic (1c–1f) alkyl NHP esters participate in this reaction to provide good yields (3a–3f, 67–78%). We were pleased to find that different functional groups such as amide (1g) and ketone (1h) can be tolerated in our reaction conditions to produce the corresponding 6-substituted phenanthridines (3g and 3h) in 65% and 68% yield. Tertiary alkyl NHP esters were smoothly converted to 6-alkyl phenanthridines in good yields (3i–3k, 70–77%). Primary alkyl NHP-esters containing variety of functional groups readily participated in this reaction and gave corresponding 6-alkyl phenanthridines in good to moderate yields. Primary alkyl NHP-esters derived from isovaleric acid, 4-phenyl buteric acid and 3-bromo-phenyl proponoic acid gave the corresponding 6-substituted phenanthridines (3l–3n) in moderate yields (60–63%). Terminal alkynes (1o) and alkenes (1p) are tolerated under these reaction conditions gave the corresponding products 3o and 3p in 58% and 56% yield. Also, primary alkyl NHP-esters (1q and 1r) derived from 2-methoxy aceticacid and 4-oxopentanoic acid were successfully converted to 6-alkyl phenanthridines 3q and 3r in moderate yields (57% and 56%).

Next, the scope of 2-isocyanobiaryls was examined. As shown in Fig. 3, first, the electronic variation at the *para* position of the Ar^2^ ring was studied with NHP-esters (1a and 1i). Electron withdrawing groups such as cyano, fluoro, chloro, bromo, trifluoro methyl, trifluoromethoxy groups were all well tolerated, giving 6-substituted phenanthridines (3s–3x) in 50–65% yields. Electron donating groups such as *t*butyl, ethyl, methoxy gave the corresponding 6-alkyl substituted phenanthridines (3y–3aa) in 72–75% yields. Next *ortho*- and *meta*-substituted 2-isocyano biphenyls were tested in our reaction conditions. In case of *ortho*-phenyl substituted 2-isocyano biphenyl gave the corresponding substituted phenanthridines (3ab) in 58% yield. In case of *meta*-methyl substituted 2-isocyano biphenyl gave the corresponding 6-alkyl phenanthridine (3ac) as a single regio-isomer in 66% yield. In case of *meta*–*para* substituted 2-isocyano biphenyl gave the 6-alkyl phenanthridines (3ad) as mixture of regio-isomers 65% yield. Dibenzothiofuran and benzyloxy groups can be tolerated in our reaction conditions. In case of 2-isocyano biphenyl containing dibenzothiofuran with alkyl NHP-ester (1i) gave the 6-alkyl phenantharidine (3ae) in 57% yield. Finally, we tested one example of 2-isocyanobiphenyl (2a) by introducing substitution on Ar^1^ ring was studied with NHP-esters (1a) in our reaction conditions provided 6-alkyl phenantharidine (3af) in 71% yield. Further, we have found that Katritzky's *N*-cyclohexylpyridinium salt (4) and Togni's reagent (5) can be activated by PPh_3_/NaI system to deliver 3a (75% yield) and 6-trifluoromethyl substituted phenanthridine (3ag) in good yield (78% yield, see ESI†).

**Fig. 4:**
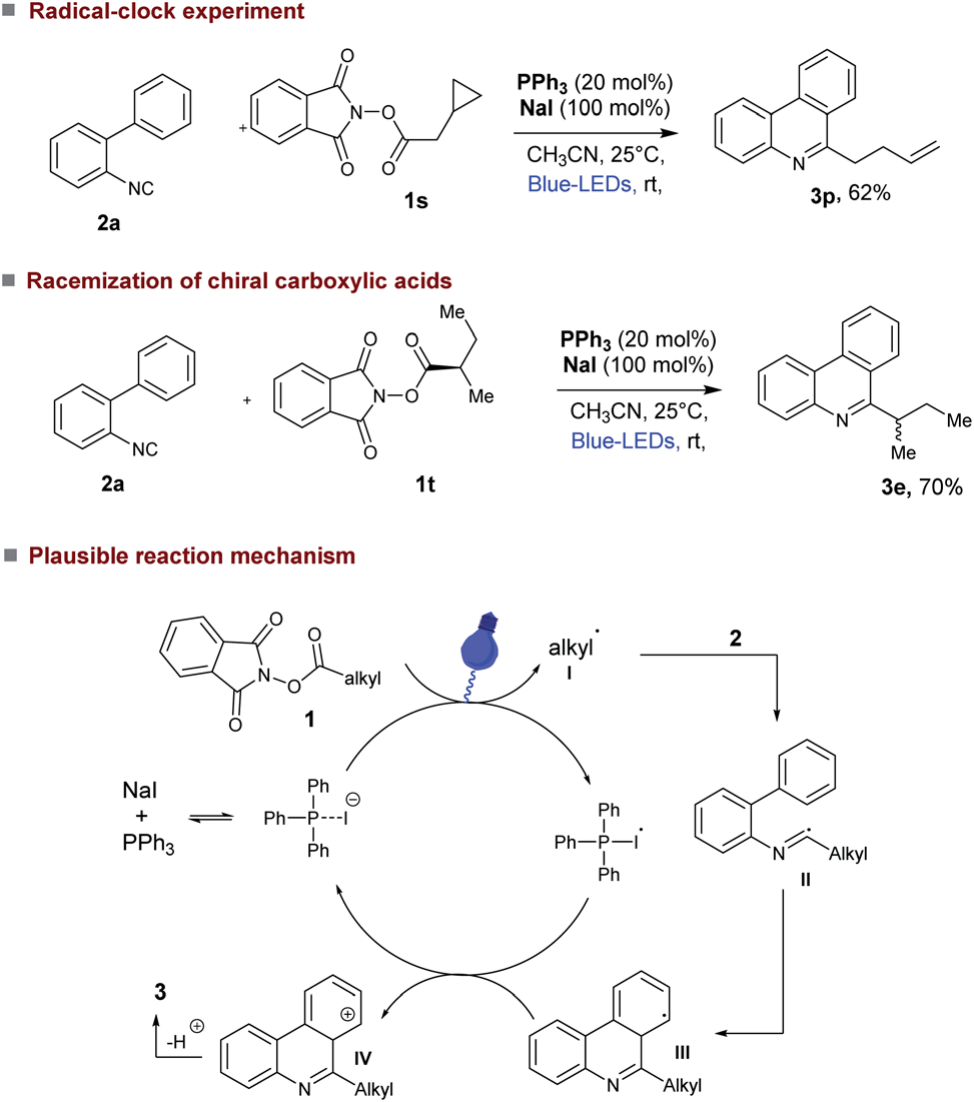
Preliminary mechanistic investigations; (Upper) radical clock experiment; (Center) racemization of chiral alkyl NHP-ester; (Bottom) plausible reaction mechanism.

The efficiency of our photocatalytic decarboxylative radical cascade cyclization leads us to conduct some preliminary mechanistic studies (Fig. 4). As anticipated ON/OFF experiment indicated that our reaction required continuous blue light irradiation to proceed (see ESI†). We currently believe that this decarboxylative cascade cyclization reaction could proceed *via* the generation of alkyl radicals. In a radical clock experiment using alkyl NHP-ester (1s) derived from 2-cyclopropylacetic acid under our reaction conditions, the ring-opened product 3p was isolated (Fig. 4, upper). Moreover, secondary chiral alkyl NHP-ester (1t) derived from enantiopure (*S*)-2-methylbutanoic acid provided the racemic 6-alkyl substituted phenanthridines product 3e (Fig. 4, center).

Based on these experimental observations and the report of Rui Shang and Yao Fu *et al.*^4*d*^ we propose that the decarboxylative cascade cyclization reaction proceeds *via* formation of charge transfer complex (CTC)^23^ (see ESI† for UV-visible absorption spectra) between PPh_3_, NaI and NHP-ester (1a) (Fig. 4).^4*d*^ After photo fragmentation of this CTC complex generates key alkyl radical I and PPh_3_-I˙. Subsequently, the generated alkyl radical is added to 2-isocyanobiaryl 2, which produce imine radical II that can form intermediate III through an intramolecular radical cyclization. Further oxidation of III by PPh_3_-I˙ (*E*_red_ = 0.69 *vs.* SCE)^4*d*^ produces the corresponding carbocation IV and NaI, PPh_3_. The carbocation IV loses a proton under reaction conditions to provide the desired product 3.

## Conclusions

In summary, we have developed a general strategy for the catalytic, radical decarboxylative cascade alkyl arylation of alkyl NHP-esters using an inexpensive PPh_3_/NaI system as a photocatalyst. This operationally simple protocol allows an efficient synthesis of 6-substituted phenanthridines in moderate to good yields under metal free conditions. Furthermore we showed that in our photocatalytic conditions, different carbon radical precursors can be activated to deliver 6-substituted phenanthridines.

## Conflicts of interest

There are no conflicts to declare.

## Supplementary Material

RA-010-D0RA03211E-s001
