# Supplementary material for: PPh3/NaI driven photocatalytic decarboxylative radical cascade alkylarylation reaction of 2-isocyanobiaryls
Source: RSC Adv. 2020 Apr 25;10(28):16510–4. doi: 10.1039/d0ra03211e (PMC9052874; doi:10.1039/d0ra03211e)
Supplement: RA-010-D0RA03211E-s001 [file RA-010-D0RA03211E-s001.pdf]

Electronic Supplementary Material (ESI) for RSC Advances.
This journal is © The Royal Society of Chemistry 2020

Supporting Information

PPh_3/NaI driven photocatalytic decarboxylative radical cascade alkylarylation reaction of 2-isocyanobiaryls

Ketan Wadekar,[a‡] Suraj Aswale[b‡] and Veera Reddy Yatham[*b]

[a]CSIR-Indian Institute of Chemical Technology, Hyderabad, India, and Academy of Scientific and Innovative Research (AcSIR), Ghaziabad, India

[b]CSIR-Indian Institute of Chemical Technology (CSIR-IICT) Hyderabad 500007, India

Dr. V. R. Yatham, Assistant professor, School of Chemistry, Indian Institute of Science Education and Research, Thiruvananthapuram, 695551, India.

[‡]Equally contributed to this work

*e-mail: reddy@iisertvm.ac.in, reddy.iisc@gmail.com,

Table of contents

General Considerations: S3

Synthesis and characterization of alkyl NHP-esters: S4

Synthesis and characterization of 2-isocyanobiaryls S5

Optimization details: S9

Synthesis and characterization of products: S12

Mechanistic studies S29

NMR spectra S33

General Considerations:

Unless otherwise stated, all commercial reagents were used without additional purification. All reactions were conducted in 10 ml crimp glass vials. Analytical thin layer chromatography (TLC) was performed on precoated silica gel 60 F254 plates. Visualization via TLC was achieved by the use of UV light (254 nm). Column chromatography was undertaken on silica gel (100-200 mesh) using a proper eluent system. NMR spectra were recorded in chloroform-d at 300, 400 and 500 MHz for ^{1}H NMR spectra and 75, 100 or 125 MHz for ^{13}C NMR spectra. ^{19}F NMR NMR spectra were recorded in chloroform-d at 377 MHz. Chemical shifts are quoted in parts per million referenced to the appropriate solvent peak or 0.0 ppm for tetramethylsilane. The following abbreviations were used to describe peak splitting patterns when appropriate: s, singlet; d, doublet; t, triplet; q, quartet; sept, septet; dd, doublet of doublets; td, triplet of doublets; m, multiplet. Coupling constants, J, are reported in hertz. For ^{13}C NMR, chemical shifts are reported in parts per million referenced to the center of a triplet at 77.0 ppm of chloroform-d. FT-IR spectra were acquired using an Agilent Cary 630 bench-top spectrometer. High-resolution mass spectra (HRMS) [ESI^{+}] were obtained using either a TOF or a double-focusing spectrometer. Optical rotations were measured using an Anton Paar MCP200 polarimeter (10.0 cm cell path) at 20.0°C with at 589 nm wavelength in analytical grade chloroform. The UV-Vis measurements were performed on Shimadzu UV-1800 spectrometer.

The deactivated silica gel (35 wt% H_2O) was prepared by mixing silica gel and deionized water, followed by vigorous shaking until a fluffy powder was observed.

Photochemical reactions were irradiated with 455 nm LEDs (OSRAM Oslon® SSL 80 royal-blue LEDs (λ_{max}= 455 nm (± 15 nm), 3.5 V, 700 mA), which were installed on a passive cooling system at the bottom (7 mm from the bottom-plane of the vials) of a custom-made 6-vials reactor (aluminium), which was equipped with a liquid cooling system (see **Figure 1**).

Synthesis and characterization of alkyl NHP-esters:

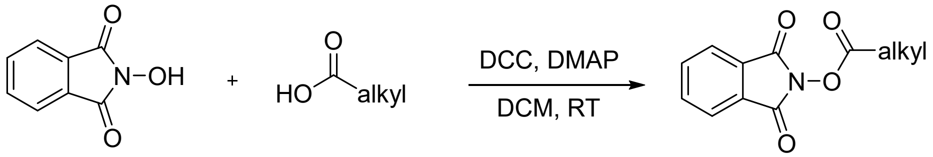

General procedure (A) for the synthesis of alkyl NHP-esters[1]

A round bottom flask was charged with carboxylic acid (1.0 equiv.), N-hydroxyphthalimide (NHPI, 1.0 equiv.) and DMAP (0.1 equiv.), CH_2Cl_2 (0.2 M) was added, followed by N,N'-dicyclohexylcarbodiimide (DCC, 1.1 equiv.), both at room temperature (RT). The mixture was allowed to stir until all the acid was consumed (as indicated by TLC). The resulting mixture was quickly filtered and the solid residue was rinsed with more CH_2Cl_2. The filtrate was concentrated in vacuo and purified by flash column chromatography using deactivated silica gel (35 wt% H_2O) to afford the corresponding alkyl NHP-ester, which was used without further purification unless otherwise noted.

Alkyl NHP-esters **1a-1f** were prepared according to known literature procedure.[1]

Alkyl NHP-esters **1g, 1i, 1m,** were prepared according to known literature procedure.[2]

Alkyl NHP-esters **1j** was prepared according to known literature procedure.[3]

Alkyl NHP-esters **1l** was prepared according to known literature procedure.[4]

Alkyl NHP-esters **1n** was prepared according to known literature procedure.[5]

Alkyl NHP-esters **1o** was prepared according to known literature procedure.[6]

Alkyl NHP-esters **1p** was prepared according to known literature procedure.[7]

Alkyl NHP-esters **1r** was prepared according to known literature procedure.[8]

Synthesis of Alkyl NHP-esters 1k and 1q.

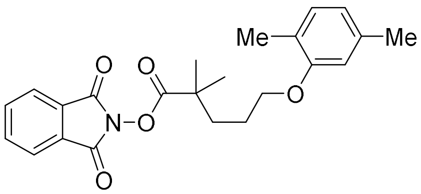

1,3-dioxoisoindolin-2-yl 5-(2,5-dimethylphenoxy)-2,2-dimethylpentanoate (1k): was prepared according to the general procedure (A)[1] from the corresponding 5-(2,5-dimethyl-phenoxy)-2,2-dimethylpentanoic acid (625 mg, 2.5 mmol, 1.0 equiv.), obtained alkyl NHP-ester **1k** as a white solid (819 mg, 83 yield). ^{1}H NMR (400 MHz, $CDCl_3$) 7.90-7.87 (m, 2H), 7.80-7.78 (m, 2H), 7.01 (d, 1H, J = 7.6 Hz), 6.67-6.66 (m, 2H), 4.02 (t, J = 7.5 Hz, 2H), 2.32

(s, 3H), 2.30 (s, 3H), 1.96 (brs, 4H),1.46 (s, 6H). ^{13}C NMR (100 MHz, $CDCl_3$) δ 173.89, 162.21, 157.07, 136.60, 134.80, 130.38, 129.17, 123.99, 123.72, 120.81, 112.12, 77.48, 77.16, 76.84, 67.85, 42.10, 37.52, 25.26, 25.12, 21.52, 15.91. FT-IR (neat, cm^{-1}):2931, 1790, 1748, 1697, 1586, 1512, 1465, 1374, 1267, 1137, 1049. HRMS (ESI): m/z calcd for $C_{23}H_{26}NO_5$ $(M+H)^+$: 396.1805, found: 396.0791.

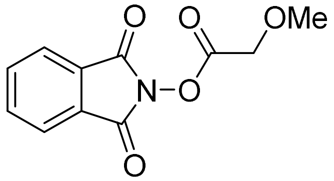

1,3-dioxoisoindolin-2-yl 2-methoxyacetate (1q): was prepared according to the general procedure (A) from the corresponding 2-methoxyacetic acid (450 mg, 5 mmol, 1.0 equiv.), obtained alkyl NHP-ester (**1q)** as a white solid (0.9 g, 80 yield). ^{1}H NMR (400 MHz, $CDCl_3$) 7.91-7.89 (m, 2H), 7.81-7.8 (m, 2H), 4.45 (s, 2H), 3.55 (s, 3H), ^{13}C NMR (100 MHz, $CDCl_3$) δ 166.8, 161.7, 135.0, 128.9, 124.2, 67.8, 59.9. FT-IR (neat, cm^{-1}):2930, 2856, 2117, 1827, 1743, 1660, 1525, 1456, 1362, 1080. HRMS (ESI): m/z calcd for $C_{11}H_9NNaO_5$ $(M+Na)^+$: 258.0373, found: 258.0391.

Synthesis and characterization of 2-isocyanobiaryls

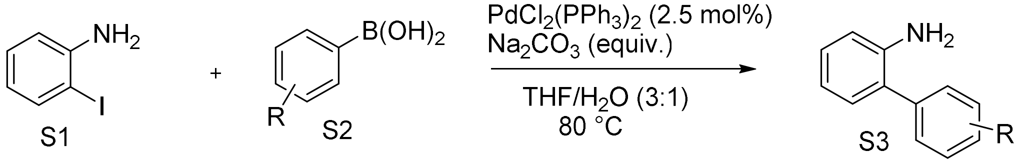

General procedure for the synthesis of biarylanilines (B):

To a 50 ml RBF was charged with 2-iodo aniline (**S1**, 5 mmol), arylboronic acid (**S2**, 5.5 mmol, 1.1 equiv.), Na_2CO_3 (2 equiv.) $PdCl_2(PPh_3)_2$ (5 mol%) and degassed THF/H_2O (3:1 ratio, 15 ml). The reaction mixture was heated at 80 ºC for 12 h under N_2 atmosphere. The resultant mixture was extracted with EtOAc (3 × 30 mL). The combined layers were washed with brine, dried over anhydrous Na_2SO_4, filtered and concentrated under reduced pressure. The residue was purified by column chromatography (90:10 Hexane/EtOAc) to provide the corresponding biarylaniline (**S3**) as pale yellow oil.

General procedure for the synthesis of 2-isocyanobiaryls from biarylamines (C)

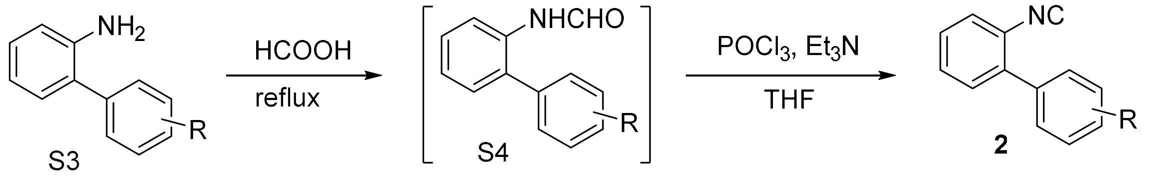

To a 25 ml RBF flask was charged with biaryl aniline (**S3**, 5 mmol) and formic acid (85% aq) (1.5 mL) in toluene (10 mL) is refluxed under N_2 atmosphere. The reaction was monitor by TLC (hexane/AcOEt = 3:1). After the reaction, volatile materials were evaporated under reduced pressure. The resulting residue containing the biarylformanilide **S4** was taken on to the next step without additional purification. To this biarylformanilide **S4**, dry THF (15 mL) and NEt_3 (25 mmol) were added with syringe. Then the mixture was cooled to 0 ^{o}C, $POCl_3$ (10 mmol, 2 equiv) was added dropwise with a syringe to the stirring solution at 0 ^{o}C for 2 h. The mixture was stirred at 0 ^{o}C for 2 h and at room temperature over night. After the reaction was complete (monitored by TLC), the mixture was quenched and neutralized by saturated aqueous solution of $NaHCO_3$. The aqueous layer was extracted with EtOAc for 3 times (50 mL × 3). Then the combined organic phases were dried over anhydrous Na_2SO_4. The solvent was removed under reduced pressure and the residue was purified by column chromatography on silica gel by using a mixture of hexane/EtOAc as an eluent to provide isocyanide **2** as green liquid. Further the colour impurities was removed by treatment of this compound with charcoal to provide pure isocyandes (2) as a light green liquid.

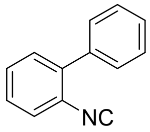

2a

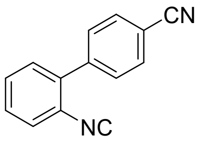

2b

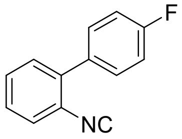

2c

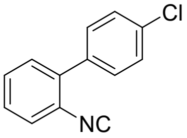

2d

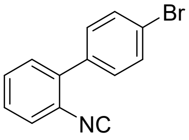

2e

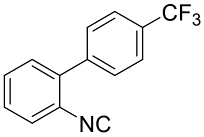

2f

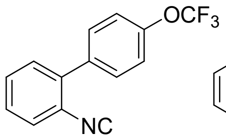

2g

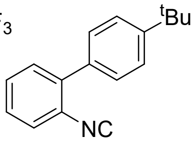

2h

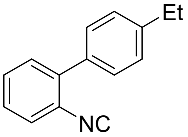

2i

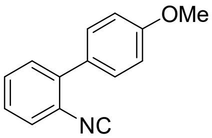

2j

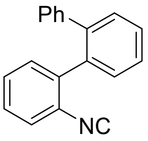

2k

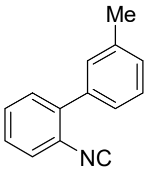

2l

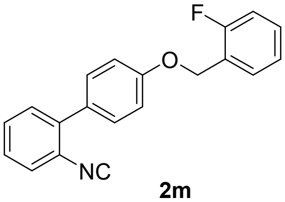

2m

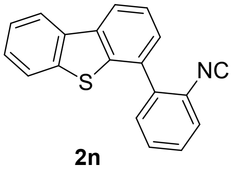

2n

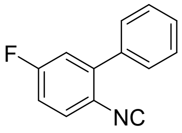

2o

2-isocyano biaryls **2h, 2e, 2f, 2g, 2n,** were prepared according to known literature procedure.[9]

2-isocyano biaryls **2b, 2j, 2l** were prepared according to known literature procedure.[10]

2-isocyano biaryl **2c, 2d, 2o** were prepared according to known literature procedure.[11]

Synthesis of compounds 2i, 2ab, 2ad.

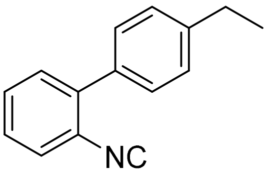

4'-ethyl-2-isocyano-1,1'-biphenyl (2i) was prepared from the corresponding biarylamine according to the general procedure (C) described above, obtained **2i** as a green liquid (865 mg, 84% over two steps). ^{1}H NMR (400 MHz, $CDCl_3$) 7.49-7.43 (m, 5H), 7.36-7.31 (m, 3H), 2.72 (q, J = 7.5 Hz, 2H), 1.29 (t, J = 7.5 Hz, 3H). ^{13}C NMR (100 MHz, $CDCl_3$) δ 166.4, 144.6, 138.9, 134.4, 130.6, 129.6, 129.0 (2C), 128.2 (2C), 127.9 (2C), 28.7, 15.5. HRMS (ESI): m/z calcd for $C_{15}H_{14}N$ $(M+H)^+$: 208.1121, found: 208.1131

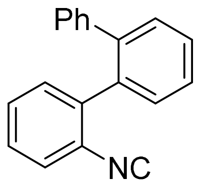

2-isocyano-1,1':2',1''-terphenyl (2k) was prepared from the corresponding biarylamine according to the general procedure (C) described above, obtained **2k** as a green liquid (1.1 g, 88% over two steps). ^{1}H NMR (400 MHz, $CDCl_3$) 7.53-7.46 (m, 3H), 7.41-7.39 (m, 1H), 7.31-7.27 (m, 3H), 7.24-7.21 (m, 4H), 7.16-7.14 (m, 2H). ^{13}C NMR (100 MHz, $CDCl_3$) δ 166.1, 141.6, 140.8, 139.4, 135.7, 131.8, 130.53, 130.51, 129.7 (2C), 128.9 (2C), 127.9 (2C), 127.5, 127.1, 126.9. HRMS (ESI): m/z calcd for $C_{19}H_{14}N$ $(M+H)^+$: 256.1121, found: 256.1132.

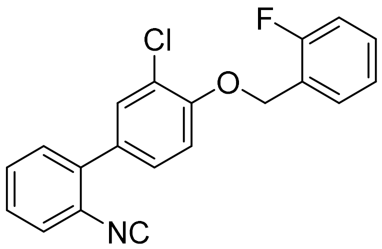

3'-chloro-4'-((2-fluorobenzyl)oxy)-2-isocyano-1,1'-biphenyl (2m) was prepared from the corresponding biarylamine according to the general procedure (C) described above, obtained **2m** as a green liquid (1.3 g, 79% over two steps). ^{1}H NMR (400 MHz, $CDCl_3$) 7.65-7.62 (m, 1H), 7.57 (d, 2.5 Hz, 1H), 7.49-7.31 (m, 6H), 7.23-7.19 (m, 1H), 7.13-7.09 (m, 2H), 5.29 (s, 2H). ^{13}C NMR (100 MHz, $CDCl_3$) δ 166.8, 160.0 (d, JC-F = 243 Hz), 154.1, 137.1, 130.9, 130.73, 130.4, 129.8 (d, JC-F = 8 Hz), 129.6, 129.3 (d, JC-F = 3.0 Hz), 128.4, 128.3, 127.9, 124.5 (d, JC-F = 2 Hz), 123.6, 123.4, 115.4, 115.2, 113.6, 64.6. ^{19}F NMR (377 MHz, $CDCl_3$): δ -118.7. HRMS (ESI): m/z calcd for $C_{20}H_{13}NClFNO$ $(M+H)^+$: 338.0742 , found: 338.076

Optimization details:

General procedure for screening reactions: A 10 mL glass vial was charged with alkyl NHP-ester (0.3 mmol), 2-isocyano biphenyl (0.2 mmol), PPh_3(20 mol%), NaI (100 mol%) and a PTFE-coated stirring bar. The glass vial was sealed with a PTFE septum and dry degassed solvent (3 mL) was added to the reaction vial. The reactions were placed in a pre-programed temperature (25°C) controlled blue LED reactor (as shown in **Figure 1**) and the reaction mixture was irradiated with a 455 nm blue LED. After 24 hours, a sample of this solution was analyzed by ^1H NMR using benzyl alcohol as the internal standard to determine the yield.

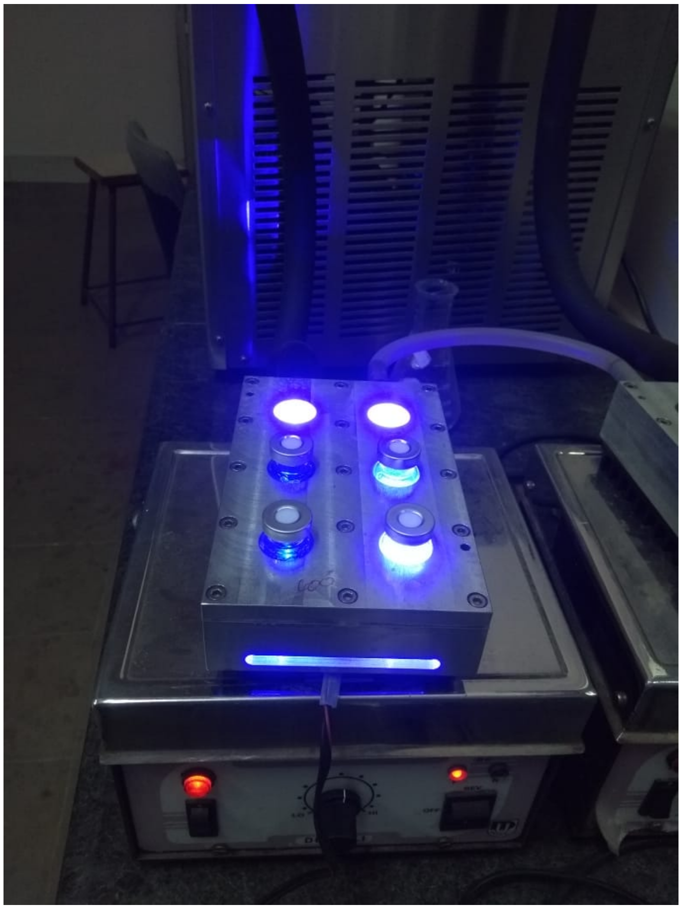

Figure 1: Blue LED reactor with magnetic stirring plate

Table S1: Screening of reaction parameters

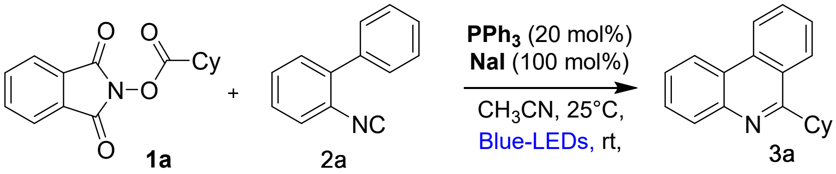

Entry	Solvents	3a (%)[a]
1	***none***	84(78)[b]
2	**KI** instead of **NaI**	80
3	**TBAI** instead of **NaI**	66
4	**LiI** instead of **NaI**	38
5	**DMSO** instead of $\mathbf{CH_3CN}$	65
6	**DMF** instead of $\mathbf{CH_3CN}$	60
7	**Acetone** instead of $\mathbf{CH_3CN}$	44
8	$\mathbf{CHCl_3}$ instead of $\mathbf{CH_3CN}$	33
9	**EtOAc** instead of $\mathbf{CH_3CN}$	28
10	$\mathbf{P(Cy)_3}$ instead of $\mathbf{PPh_3}$	5
11	**P(*o*-tol)**$_3$ instead of $\mathbf{PPh_3}$	2
12	$\mathbf{Et_3N}$ instead of $\mathbf{PPh_3}$	4
13	**DMAP** instead of $\mathbf{PPh_3}$	3
14	without **NaI**	18
15	without $\mathbf{PPh_3}$	0
16	without light **NaI** and $\mathbf{PPh_3}$	0
17	with out **blue light**	0
18	with **blue light (410 nm)**	80
19	**DPPF** instead of $\mathbf{PPh_3}$	7
20	**DPPP** instead of $\mathbf{PPh_3}$	0
21	***Tris(o-furyl)phosphine*** instead of $\mathbf{PPh_3}$	0
22	$\mathbf{PPh_3}$ (5 mol%) instead of 10 mol% $\mathbf{PPh_3}$	40
23	**NaI** (50 mol%) instead of 100 mol% **NaI**	76
24	**Dioxane** instead of $\mathbf{CH_3CN}$	13
25	**Toulene** instead of $\mathbf{CH_3CN}$	6
26	**DCE** instead of $\mathbf{CH_3CN}$	7
27	**DIPEA** instead of $\mathbf{PPh_3}$	0
28	**DABCO** instead of $\mathbf{PPh_3}$	0%

[a]Determined by ^{1}H NMR, using benzyl alcohol as internal standard. [b]Isolated yield.

General procedure for decarboxylative radical cascade alkylarylation reaction of 2-isocyanobiaryls

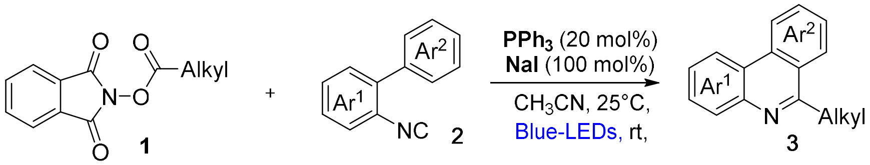

General procedure (GP1): A 10 mL glass vial equipped with a teflon-coated stirring bar was charged with alkyl NHP-ester (0.15- 0.3 mmol), 2-isocyanobiaryl (0.1-0.2 mmol), PPh_3(20 mol%), NaI (100 mol%). The glass vial was sealed with a PTFE septum and dry degassed CH_3CN (3 mL) was added. The reaction was placed in a pre-programed temperature (25°C) controlled blue LED reactor (as shown in **Figure 1**) and the reaction mixture was irradiated with a 455 nm blue LED. After 24 hours, the reaction mixture was concentrated under reduced pressure. The product **3** was purified by flash chromatography on silica (hexane:AcOEt 95:5, followed by hexane:AcOEt 90:10).

Note: At same time we run two independent reactions for each substrate and the obtained yield was the combined average yield of these two independent runs.

Visual representation of the reaction set-up

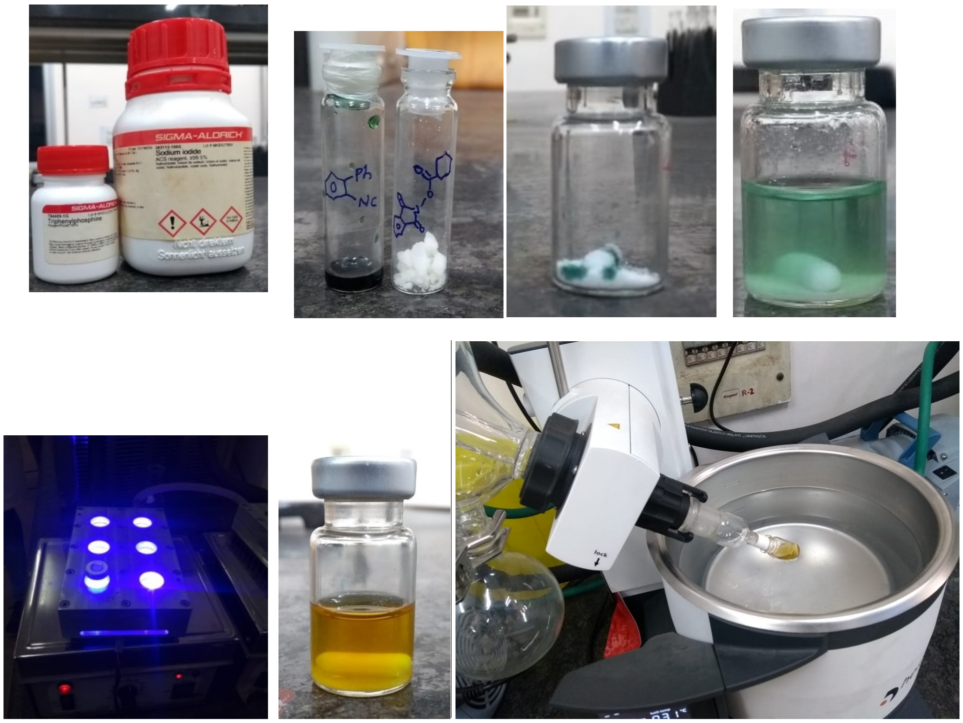

Figure 2: Visual representation of the reaction set-up.

Synthesis and characterization of products:

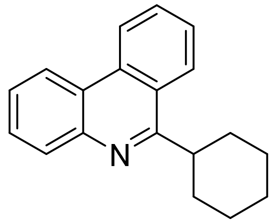

6-cyclohexylphenanthridine (3a): Following the general procedure **GP1**, two reactions of **2a** (0.2 mmol each one, 35.8 mg) with **1a** (0.3 mmol each one,81.9 mg) and after 24 hours, the reaction mixture was concentrated under reduced pressure. The product **3a** was purified by flash chromatography on silica (hexane:AcOEt 95:5, followed by hexane:AcOEt 90:10) afforded as yellowish oil (81.4 mg, 78% yield). ^{1}H NMR (400 MHz, $CDCl_3$) δ 8.65 (d, J = 8.0 Hz, 1H), 8.54 (dd, J = 8.0 Hz, 0.8 Hz 1H), 8.32 (d, J = 8.4 Hz, 1H), 8.15 (dd, J = 8.0 Hz, 0.8 Hz, 1H), 7.81 (td, J = 7.6 Hz, 1.2 Hz, 1H), 7.72-7.67 (m, 2H), 7.60 (td, J = 7.6 Hz, 1.2 Hz, 1H), 3.65 – 3.58 (m, 1H), 2.10-1.83 (m, 7H), 1.59-1.45 (m, 3H). ^{13}C NMR (100 MHz, $CDCl_3$) δ 165.4, 144.0, 133.1, 130.07, 130.06, 128.5, 127.1, 126.2, 125.7, 124.8, 123.4, 122.7, 121.9, 42.1, 32.4, 27.0, 26.4. The analytical data are consistent with published ones. [12]

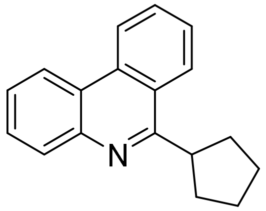

6-cyclopentylphenanthridine (3b): Following the general procedure **GP1**, two reactions of **2a** (0.2 mmol, 35.8 mg each one) with **1b** (0.3 mmol, 77.7 mg each one) and after 24 hours, the reaction mixture was concentrated under reduced pressure. The product **3b** was purified by flash chromatography on silica (hexane:AcOEt 95:5, followed by hexane:AcOEt 90:10) afforded as yellowish oil (75 mg, 76% yield). ^{1}H NMR ($CDCl_3$, 400 MHz): δ 8.64 (d, J = 8.2 Hz, 1H), 8.53 (dd, J = 8.1 Hz, 0.8 Hz, 1H), 8.34 (d, J = 8.2 Hz, 1H), 8.14 (dd, J = 8.1 Hz, 0.8 Hz, 1H), 7.81 (td, J = 8.1 Hz, 1.2 Hz, 1H), 7.72-7.66 (m, 2H), 7.60 (td, J = 8.1 Hz, 0.8 Hz, 1H), 4.08 (pent, J = 8.0 Hz, 1H), 2.31-2.17 (m, 4H), 1.97-1.91 (m, 2H), 1.83-1.78 (m, 2H); ^{13}C NMR ($CDCl_3$, 100 MHz): δ 164.3, 143.8, 133.07, 130.09, 130.0, 128.5, 127.1, 126.3, 126.2, 125.8, 123.6, 122.5, 121.9, 43.7, 32.3, 26.2. The analytical data are consistent with published ones.[12]

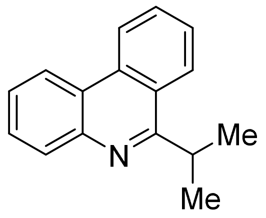

6-isopropylphenanthridine (3c): Following the general procedure GP1, two reactions of **2a** (0.2 mmol, 35.8 mg each one) with **1c** (0.3 mmol, 69.9 mg each one) and after 24 hours, the reaction mixture was concentrated under reduced pressure. The product **3c** was purified by flash chromatography on silica (hexane:AcOEt 95:5, followed by hexane:AcOEt 90:10) afforded as colorless oil (62.8 mg, 71% yield). ^{1}H NMR (400 MHz, $CDCl_3$) δ 8.65 (d, J = 8.2 Hz, 1H), 8.54 (d, J = 8.1 Hz, 1H), 8.33 (d, J = 8.2 Hz, 1H), 8.15 (d, J = 8.0 Hz, 1H), 7.82 (t, J = 7.6 Hz, 1H), 7.72-7.68 (m, 2H), 7.61 (t, J = 7.5 Hz, 1H), 4.01 (sept, J = 6.8 Hz, 1H), 1.53 (d, J = 6.8 Hz, 6H); ^{13}C NMR (100 MHz, $CDCl_3$) δ 165.9, 143.9, 133.2, 130.06 (2C), 128.5, 127.2, 126.3, 125.8, 124.8, 123.5, 122.7, 121.9, 31.6, 22.09.The analytical data are consistent with published ones.[13]

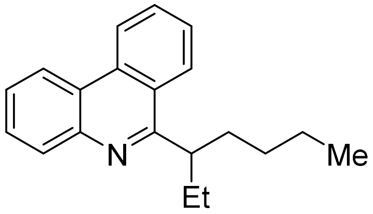

6-(heptan-3-yl)phenanthridine (3d): Following the general procedure GP1, two reactions of **2a** (0.2 mmol, 35.8 mg each one) with **1d** (0.3 mmol, 86.7 mg each one) and after 24 hours, the reaction mixture was concentrated under reduced pressure. The product **3d** was purified by flash chromatography on silica (hexane:AcOEt 95:5, followed by hexane:AcOEt 90:10) afforded as a white solid (83 mg, 75% yield). ^{1}H NMR (400 MHz, $CDCl_3$) δ 8.68 (d, J = 8.2 Hz, 1H), 8.57 (d, J = 8.1 Hz, 1H), 8.35 (d, J = 8.3 Hz, 1H), 8.15 (d, J = 8.0 Hz, 1H), 7.83 (td, J = 7.6 Hz, 1.2 Hz, 1H), 7.73 – 7.68 (m, 2H), 7.62 (td, J = 7.6 Hz, 1.2 Hz, 1H), 3.69 – 3.64 (m, 1H), 2.15 – 2.08 (m, 2H), 1.90 – 1.85 (m, 2H), 1.31 – 1.27 (m, 4H), 0.88-0.81 (m, 6H). ^{13}C NMR (100 MHz, $CDCl_3$) δ 165.07, 144.07, 132.98, 130.08, 130.02, 128.47, 127.19, 126.34, 126.19, 125.82, 123.31, 122.64, 121.95, 43.73, 34.73, 30.28, 28.20, 23.16, 14.19, 12.59. The analytical data are consistent with published ones.[14]

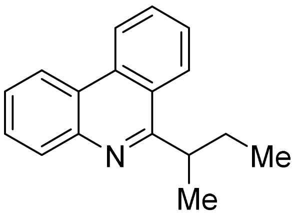

6-(sec-butyl)phenanthridine (3e): Following the general procedure GP1, two reactions of **2a** (0.2 mmol, 35.8 mg each one) with **1e** (0.3 mmol, 74.1 mg each one) and after 24 hours, the reaction mixture was concentrated under reduced pressure. The product **3e** was purified by flash chromatography on silica (hexane:AcOEt 95:5, followed by hexane:AcOEt 90:10) afforded as colorless oil (68.6 mg, 73% yield). ^{1}H NMR (400 MHz, $CDCl_3$) δ 8.66 (d, J = 8.2 Hz, 1H), 8.55 (d, J = 8.1 Hz, 1H), 8.34 (d, J = 8.3 Hz, 1H), 8.14 (d, J = 8.1 Hz, 1H), 7.84 – 7.80 (m, 1H), 7.72 – 7.68 (m, 2H), 7.63 – 7.60 (m, 1H), 3.80 – 3.73 (m, 1H), 2.22 – 2.13 (m, 1H), 1.87 – 1.78 (m, 1H), 1.49 (d, J = 6.8 Hz, 3H), 0.99 (t, J = 7.4 Hz, 3H). ^{13}C NMR (100 MHz, $CDCl_3$) δ 165.48, 143.89, 133.03, 129.96 (2C), 128.41, 127.11, 126.16, 125.68, 125.29, 123.35, 122.59, 121.85, 38.36, 29.21, 19.82, 12.50. The analytical data are consistent with published ones.[15]

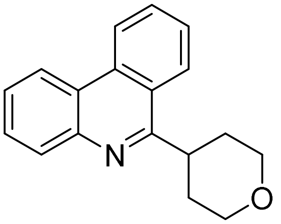

6-(tetrahydro-2*H*-pyran-4-yl)phenanthridine (3f): Following the general procedure GP1, two reactions of **2a** (0.2 mmol, 35.8 mg each one) with **1f** (0.3 mmol, 82.5 mg each one) and after 24 hours, the reaction mixture was concentrated under reduced pressure. The product **3f** was purified by flash chromatography on silica (hexane:AcOEt 90:10, followed by hexane:AcOEt 80:20) afforded as a white solid (70.5 mg, 67%). ^{1}H NMR (400 MHz, $CDCl_3$) δ 8.66 (d, J = 8.4 Hz, 1H), 8.55 (d, J = 8.1 Hz, 1H), 8.30 (d, J = 8.4 Hz, 1H), 8.14 (d, J = 8.1 Hz, 1H), 7.83 (td, J = 7.4 Hz, 1.1 Hz, 1H), 7.74-7.68 (m, 2H), 7.65-7.61 (td, J = 7.5 Hz, 1.1 Hz, 1H), 4.23-4.18 (m, 2H), 3.91-3.83 (m, 1H), 3.77-3.71 (m, 2H), 2.41-2.30 (m, 2H), 1.99-1.95 (m, 2H). ^{13}C NMR (100 MHz, $CDCl_3$) δ 163.09, 143.9, 133.2, 130.2 (2C), 128.6, 127.3, 126.5, 125.3, 124.6, 123.5, 122.9, 121.9, 68.4, 39.3, 32.1. The analytical data are consistent with published ones.[16]

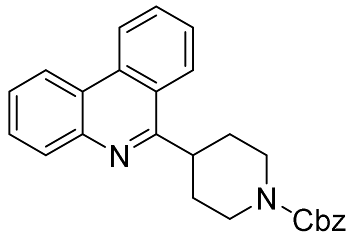

benzyl 4-(phenanthridin-6-yl)piperidine-1-carboxylate (3g): Following the general procedure GP1, two reactions of **2a** (0.1 mmol, 17.9 mg each one) with **1g** (0.15 mmol, 61.2 mg each one) and after 24 hours, the reaction mixture was concentrated under reduced pressure. The product **3g** was purified by flash chromatography on silica (hexane:AcOEt 90:10, followed by hexane:AcOEt 80:20) afforded as yellowish solid (51.5 mg, 65% yield). ^{1}H NMR (400 MHz, $CDCl_3$) δ 8.67 (d, J = 8.4 Hz, 1H), 8.55 (d, J = 8.1 Hz, 1H), 8.28 (d, J = 8.4 Hz, 1H), 8.11 (d, J = 8.1 Hz, 1H), 7.83 (t, J = 7.6 Hz, 1H), 7.73-7.68 (m, 2H), 7.65-7.61 (m, 1H), 7.42-7.35 (m, 5H), 5.19 (s, 2H), 4.43 (brs, 2H), 3.83-3.77 (m, 1H), 3.12 (brs, 2H), 2.20-2.06 (m, 4H), 1.99-1.95 (m, 2H). ^{13}C NMR (100 MHz, $CDCl_3$) δ 162.9, 155.4, 143.8, 137.1, 133.2, 130.3, 130.1, 128.7 (2C), 128.6 (2C), 128.1(2C), 128.0 (2C), 127.4, 126.6, 125.2, 124.6, 123.5, 122.9, 121.9, 67.2, 44.5, 39.9, 31.2. HRMS (ESI): m/z calcd for $C_{26}H_{25}N_2O_2$ $(M+H)^+$: 397.1911, found: 397.1915.

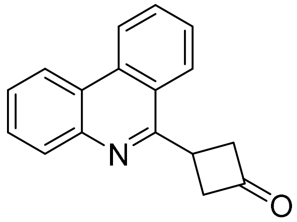

3-(phenanthridin-6-yl)cyclobutan-1-one (3h): Following the general procedure GP1, two reactions of **2a** (0.2 mmol, 35.8 mg each one) with **1h** (0.3 mmol, 77.7 mg each one) and after 24 hours, the reaction mixture was concentrated under reduced pressure. The product **3h** was purified by flash chromatography on silica (hexane:AcOEt 90:10, followed by hexane:AcOEt 80:20) afforded as a yellowish oil (67.2 mg, 68% yield). ^{1}H NMR (400 MHz, $CDCl_3$) δ 8.67 (d, J = 8.4 Hz, 1H), 8.55 (d, J = 8.1 Hz, 1H), 8.17 (t, J = 7.6 Hz, 1H), 7.88 (t, J = 7.8 Hz, 1H), 7.77-7.64 (m, 3H), 4.52-4.41 (m, 1H), 4.02-392 (m, 2H), 3.62-3.52 (m, 2H). ^{13}C NMR (100 MHz, $CDCl_3$) δ 207.0, 159.8, 143.4, 133.3, 130.7, 130.1, 128.9, 127.6, 127.0, 125.7, 124.9, 123.9, 122.9, 122.1, 52.3 (2C), 27.9. HRMS (ESI): m/z calcd for $C_{17}H_{14}NO$ $(M+H)^+$: 248.1070, found: 248.1078.

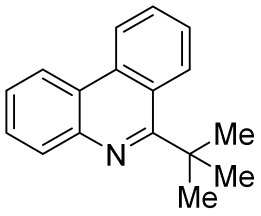

6-(tert-butyl)phenanthridine (3i): Following the general procedure GP1, two reactions of **2a** (0.2 mmol, 35.8 mg each one) with **1i** (0.3 mmol, 74 mg each one) and after 24 hours, the reaction mixture was concentrated under reduced pressure. The product **3i** was purified by flash chromatography on silica (hexane:AcOEt 95:5, followed by hexane:AcOEt 90:10) afforded as yellow oil (72.4 mg, 77% yield). ^{1}H NMR: (400 MHz, $CDCl_3$) δ 8.69 (d, J = 8.4 Hz, 1H), 8.64 (d, J = 8,4 Hz, 1 H), 8.53 (d, J = 8.0 Hz, 1 H), 8.13 (d, J = 8.0 Hz, 1 H), 7.79 (td, J = 7.6 Hz, 1.2 Hz, 1 H), 7.67-7.60 (m, 2H), 1.74 (s, 9 H);^{13}C NMR: (100 MHz, $CDCl_3$) δ 166.7, 143.1, 134.1, 130.4, 129.3, 128.5, 128.4, 126.6, 126.0, 124.4, 123.5, 123.1, 121.7, 40.3, 31.3. The analytical data are consistent with published ones.[17]

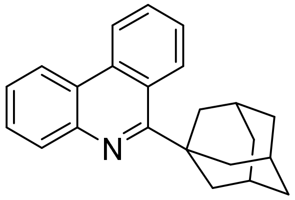

6-((3r,5r,7r)-adamantan-1-yl)phenanthridine (3j): Following the general procedure GP1, two reactions of **2a** (0.1 mmol, 35.8 mg each one) with **1j** (0.15 mmol, 48.7 mg each one) and after 24 hours, the reaction mixture was concentrated under reduced pressure. The product **3j** was purified by flash chromatography on silica (hexane:AcOEt 95:5, followed by hexane:AcOEt 90:10) afforded as white solid (45.7 mg, 73% yield). ^{1}H NMR: (400 MHz, $CDCl_3$) δ 8.85 (d, J = 8.4 Hz, 1H), 8.7 (d, J = 8,4 Hz, 1 H), 8.53 (d, J = 8.0 Hz, 1 H), 8.12 (d, J = 8.0 Hz, 1 H), 7.77 (td, J = 7.6 Hz, 1.2 Hz, 1 H), 7.71-7.60 (m, 3H), 2.49 (s, 6H), 2.24 (brs, 3H), 1.95-1.88 (m, 6H). ^{13}C NMR: (100 MHz, $CDCl_3$) δ 166.1, 143.2, 134.1, 130.3, 129.2, 128.4, 128.1, 126.5, 125.8, 124.5, 123.4, 123.2, 121.7, 43.1, 42.1, 37.3, 29.4. The analytical data are consistent with published ones.[18]

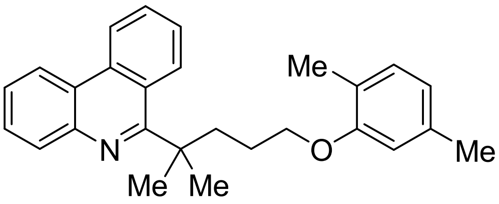

6-(5-(2,5-dimethylphenyl)-2-methylpentan-2-yl)phenanthridine (3k): Following the general procedure GP1, two reactions of **2a** (0.1 mmol, 35.8 mg each one) with **1k** (0.15 mmol, 59.3 mg, each one) and after 24 hours, the reaction mixture was concentrated under reduced pressure. The product **3k** was purified by flash chromatography on silica (hexane:AcOEt 95:5, followed by hexane:AcOEt 90:10) afforded as yellowish solid (53.6 mg, 70% yield). ^{1}H NMR: (400 MHz, $CDCl_3$) δ 8.71-8.66 (m, 2H), 8.55 (d, J = 8,4 Hz, 1 H), 8.15-8.14 (m, 1 H), 7.79 (t, J = 7.6 Hz, 1 H), 7.73-7.70 (m, 1H), 7.66-7.61 (m, 2H), 6.98-6.96 (m, 1H), 6.63-6.62 (m, 1H), 6.50 (s, 1H), 3.85-3.83 (m, 2H), 2.40-2.37 (m, 2H), 2.25 (s, 3H), 2.12 (s, 3H), 1.77 (s, 6H), 1.69-1.64 (m, 2H). ^{13}C NMR: (100 MHz, $CDCl_3$) δ 164.3, 155.9, 141.8, 135.3, 132.8, 129.3, 129.1, 128.3, 127.3, 126.5, 125.4, 125.1, 123.6, 122.4, 122.3, 121.9, 120.5, 119.4, 110.6, 67.0, 42.4, 38.5, 28.6, 24.3, 20.3, 14.7. HRMS (ESI): m/z calcd for $C_{27}H_{30}NO$ $(M+H)^+$: 384.2322, found: 384.2329

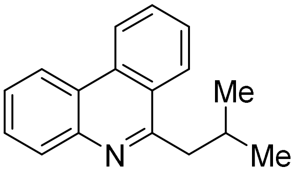

6-isobutylphenanthridine (3l) Following the general procedure GP1, two reactions of **2a** (0.2 mmol, 35.8 mg each one) with **1l** (0.3 mmol, 74 mg each one) and after 24 hours, the reaction mixture was concentrated under reduced pressure. The product **3l** was purified by flash chromatography on silica (hexane:AcOEt 95:5, followed by hexane:AcOEt 90:10) afforded as a colorless oil (57.3 mg, 61% yield). ^{1}H NMR (500 MHz, $CDCl_3$) δ 8.64 (d, J = 8.4 Hz, 1H), 8.54 (d, J = 8.0, 1H), 8.26 (d, J = 8.0 Hz, 1H), 8.15 (d, J = 8.0, 1H), 7.82 (t, J = 8.4, 1H), 7.73−7.67 (m, 2H), 7.62 (td, J = 8.4, 1.2 Hz, 1H), 3.26 (d, J = 7.2 Hz, 2H), 2.45−2.33 (m, 1H), 1.05 (d, J = 6.8 Hz, 6H); ^{13}C NMR (125 MHz, $CDCl_3$) δ 161.7, 143.8, 133.0, 130.3, 129.8, 128.7, 127.2, 126.6, 126.4, 125.7, 123.7, 122.5, 122.0, 45.0, 29.3, 23.0. The analytical data are consistent with published ones.[19]

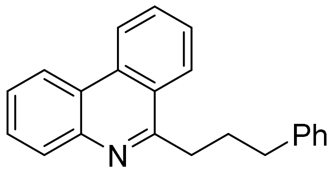

6-(3-phenylpropyl)phenanthridine (3m): Following the general procedure GP1, two reactions of **2a** (0.2 mmol, 35.8 mg each one) with **1m** (0.3 mmol, 92.7 mg each one) and after 24 hours, the reaction mixture was concentrated under reduced pressure. The product **3m** was purified by flash chromatography on silica (hexane:AcOEt 95:5, followed by hexane:AcOEt 90:10) afforded as a yellow solid (74.8 mg, 63% yield). ^{1}H NMR (400 MHz, $CDCl_3$) δ 8.64 (d, J = 8.3 Hz, 1H), 8.54 (d, J = 8.0 Hz, 1H), 8.12 (d, J = 8.1 Hz, 2H), 7.82 (t, J = 7.3 Hz, 1H), 7.75-7.60 (m, 3H), 7.33- 7.20 (m, 5H), 3.41 (t, J = 7.9 Hz, 2H), 2.87 (t, J = 7.6 Hz, 2H), 2.33-2.23 (m, 2H); ^{13}C NMR (100 MHz, $CDCl_3$) δ 160.8, 142.7, 141.1, 131.9, 129.3, 128.6, 127.6 (2C),127.5, 127.3 (2C), 126.2, 125.3, 125.2, 124.8, 124.2, 122.6, 121.5, 120.9, 35.0, 34.6, 29.9. The analytical data are consistent with published ones.[20]

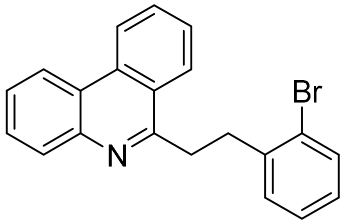

6-(2-bromophenethyl)phenanthridine (3n): Following the general procedure GP1, two reactions of **2a** (0.1 mmol, 17.9 mg each one) with **1n** (0.15 mmol, 56 mg each one) and after 24 hours, the reaction mixture was concentrated under reduced pressure. The product **3n** was purified by flash chromatography on silica (hexane:AcOEt 95:5, followed by hexane:AcOEt 90:10) afforded as a yellow oil (43.3 mg, 60% yield). and analytical data are consistent with published ones.[1] ^{1}H NMR (400 MHz, $CDCl_3$): δ = 8.65 (d, J = 8.0 Hz, 1H), 8.56 (dd, J = 8.0, 0.8 Hz, 1H), 8.36 (d, J = 8.0 Hz, 1H), 8.17 (dd, J = 8.0, 0.8 Hz, 1H), 7.86–7.81 (m, 1H), 7.76–7.58 (m, 4H), 7.39 (dd, J = 7.6, 1.2 Hz, 1H), 7.23 (td, J = 7.6, 1.2 Hz, 1H), 7.09 (td, J = 8.0, 1.6 Hz, 1H), 3.71–3.67 (m, 2H), 3.44–3.41 (m, 2H); ^{13}C NMR (100 MHz, $CDCl_3$): δ = 159.7, 142.7, 140.3, 131.8 (2C), 129.8, 129.3, 128.6, 127.6, 126.8, 126.6, 126.3, 125.4, 125.2, 124.4, 123.4, 122.7, 121.4, 120.9, 35.0, 34.4. The analytical data are consistent with published ones.[21]

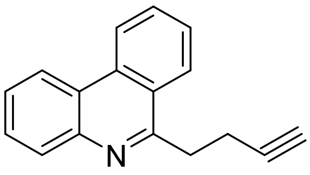

6-(but-3-yn-1-yl)phenanthridine (3o): Following the general procedure GP1, two reactions of **2a** (0.2 mmol, 35.8 mg each one) with **1o** (0.3 mmol, 72.9 mg each one) and after 24 hours, the reaction mixture was concentrated under reduced pressure. The product **3o** was purified by flash chromatography on silica (hexane:AcOEt 95:5, followed by hexane:AcOEt 90:10) afforded as a colorless oil (53.6 mg, 58% yield). and analytical data are consistent with published ones.[1] ^{1}H NMR (400 MHz, $CDCl_3$) δ 8.65 (d, J = 8.4 Hz, 1H), 8.55 (d, J = 8.0, 1H), 8.27 (d, J = 8.0 Hz, 1H), 8.13 (d, J = 8.0, 1H), 7.85 (td, J = 8.4, 1.2 Hz, 1H), 7.74-7.69 (m, 2H), 7.63 (td, J = 8.4, 1.2 Hz, 1H), 3.65-3.62 (m, 2H), 2.95−2.92 (m, 1H), 2.02 (t, J = 6.8 Hz, 1H); ^{13}C NMR (100 MHz, $CDCl_3$) δ 159.3, 143.7, 132.9, 130.5, 129.8, 128.7, 127.5, 126.7, 125.9, 125.3, 123.8, 122.6, 122.0, 84.3, 69.0, 34.5, 17.4. HRMS (ESI): m/z calcd for $C_{17}H_{14}N$ $(M+H)^+$: 232.1121, found: 232.1130.

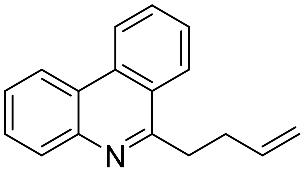

6-(but-3-en-1-yl)phenanthridine (3p): Following the general procedure GP1, two reactions of **2a** (0.2 mmol, 35.8 mg each one) with **1p** (0.3 mmol, 73.5 mg each one) and after 24 hours, the reaction mixture was concentrated under reduced pressure. The product **3p** was purified by flash chromatography on silica (hexane:AcOEt 95:5, followed by hexane:AcOEt 90:10) afforded as a yellow solid (52.2 mg, 56% yield). ^{1}H NMR: (400 MHz, $CDCl_3$) δ 8.65 (d, J = 8.2 Hz , 1H), 8.55 (dd, J = 8.2, 1.3 Hz, 1H), 8.25 (d, J = 8.1 Hz, 1H), 8.13 (dd, J = 8.1 Hz, 1.1 Hz, 1H), 7.83(td, J = 8.3, 1.3 Hz, 1H), 7.73-7.68 (m, 2H), 7.64-7.60 (m, 1H), 6.10 – 6.0 (m, 1H), 5.20-5.14 (m, 1H), 5.06-5.02 (m, 1H), 3.49 – 3.45 (m, 2H), 2.74 – 2.69 (m, 2H).^{13}C NMR: (100 MHz, $CDCl_3$) δ 161.4, 143.8, 138.1, 133.0, 130.4, 129.7, 128.7, 127.4, 126.5, 126.2, 125.3, 123.7, 122.6, 122.0, 115.2, 35.5, 33.3. The analytical data are consistent with published ones.[22]

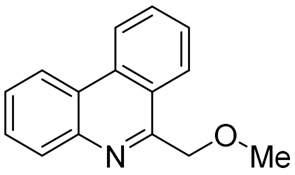

6-(methoxymethyl)phenanthridine (3q): Following the general procedure GP1, two reactions of **2a** (0.2 mmol, 35.8 mg each one) with **1q** (0.3 mmol, 70.5 mg each one) and after 24 hours, the reaction mixture was concentrated under reduced pressure. The product **3q** was purified by flash chromatography on silica (hexane:AcOEt 90:10, followed by hexane:AcOEt 80:20) afforded as a yellow oil (50.8 mg, 57% yield). ^{1}H NMR (400 MHz, $CDCl_3$) δ 8.66 (d, J = 8.4 Hz, 1H), 8.58 (dd, J = 8.0 Hz, 0.8 Hz, 1H), 8.43 (d, J = 8.0 Hz, 1H), 8.19 (d, J = 8.0, 0.8 Hz, 1H), 7.87 (td, J = 8.4, 1.2 Hz, 1H), 7.76-7.67 (m, 3H), 5.15 (s, 2H), 3.53 (s, 3H). ^{13}C NMR (100 MHz, $CDCl_3$) δ 156.1, 142.3, 132.1, 129.6, 129.2, 127.6, 126.4, 126.1, 125.6, 124.0, 123.3, 121.2, 121.0, 74.7, 57.6. HRMS (ESI): m/z calcd for $C_{15}H_{14}NO$ $(M+H)^+$: 224.1070, found: 224.1077.

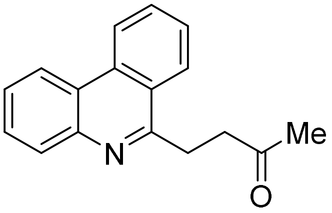

4-(phenanthridin-6-yl)butan-2-one (3r): Following the general procedure GP1, two reactions of **2a** (0.2 mmol, 35.8 mg each one) with **1r** (0.3 mmol, 78.3 mg each one) and after 24 hours, the reaction mixture was concentrated under reduced pressure. The product **3r** was purified by flash chromatography on silica (hexane:AcOEt 95:5, followed by hexane:AcOEt 90:10) afforded as a yellowish oil (55.7 mg, 56% yield). ^{1}H NMR (400 MHz, $CDCl_3$) δ 8.62 (d, J = 8.4 Hz, 1H), 8.54 (d, J = 8.0 Hz, 1H), 8.28 (d, J = 8.0 Hz, 1H), 8.05 (d, J = 8.0 Hz, 1H), 7.84 (td, J = 8.4, 1.2 Hz, 1H), 7.72-7.68 (m, 2H), 7.63-7.60 (m, 1H), 3.69 (t, J = 7.0 Hz, 2H), 3.19 (t, J = 7.0 Hz, 2H), 2.38 (s, 3H). ^{13}C NMR (100 MHz, $CDCl_3$) δ 208.8, 159.4, 143.5, 132.7, 130.4, 129.6, 128.5, 127.4, 126.4, 125.7, 125.4, 123.7, 122.4, 121.9, 40.2, 30.6, 29.1. HRMS (ESI): m/z calcd for $C_{17}H_{16}NO$ $(M+H)^+$: 250.1226, found: 250.1235.

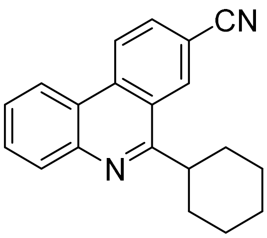

6-cyclohexylphenanthridine-8-carbonitrile (3s): Following the general procedure GP1, two reactions of **2b** (0.2 mmol, 40.8 mg each one) with **1a** (0.3 mmol, 81.9 mg each one) and after 24 hours, the reaction mixture was concentrated under reduced pressure. The product **3s** was purified by flash chromatography on silica (hexane:AcOEt 95:5, followed by hexane:AcOEt 90:10) afforded as yellowish solid (57.2 mg, 50% yield). ^{1}H NMR (400 MHz, $CDCl_3$) δ 8.71 (d, J = 8.6 Hz, 1H), 8.64 (s, 1H), 8.53 (d, J = 8.2 Hz, 1H), 8.15 (d, J = 8.2 Hz, 1H), 7.97 (d, J = 8.5 Hz, 1H), 7.79 (t, J = 7.4 Hz, 1H), 7.67 (t, J = 7.7 Hz, 1H), 3.57-3.51 (m, 1H), 2.05-1.85 (m, 7H), 1.64-1.41 (m, 2H), 1.47-1.41 (m, 1H); ^{13}C NMR ($CDCl_3$, 100 MHz): δ 164.6, 144.9, 135.8, 131.3, 131.3, 130.4, 130.3, 127.1, 124.3, 124.06, 122.5, 122.1, 119.1, 110.6, 42.1, 32.4, 26.8, 26.3. The analytical data are consistent with published ones.[23]

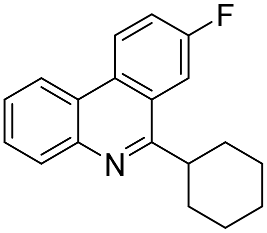

8-fluoro-6-cyclohexylphenanthridine (3t): Following the general procedure GP1, two reactions of **2c** (0.1 mmol, 19.7 mg each one) with **1a** (0.15 mmol, 40.9 mg each one) and after 24 hours, the reaction mixture was concentrated under reduced pressure. The product **3t** was purified by flash chromatography on silica (hexane:AcOEt 95:5, followed by hexane:AcOEt 90:10) afforded as a yellow solid (29.5 mg, 53% yield). ^{1}H NMR (400 MHz, $CDCl_3$) δ 8.64 (dd, J = 9.2 Hz, 4.4 Hz, 1H), 8.47 (d, J = 6.6 Hz, 1H), 8.13 (d, J = 6.6 Hz, 1H), 7.91 (dd, J = 8.4 Hz, 2.0 Hz, 1H), 7.71-7.68 (m, 1H), 7.62-7.54 (m, 2H), 3.50-3.44 (m, 1H), 2.07-1.84 (m, 8H), 1.62-1.41 (m, 2H). ^{13}C NMR (100 MHz, $CDCl_3$) δ 164.53, 161.2 (d JC-F = 245.6 Hz), 143.6, 130.2, 129.8 (d, JCF = 1.4 Hz), 128.4, 126.67, 126.2 (d JC-F = 7.5 Hz), 125.25 (d, JC-F = 8.6 Hz), 123.05, 121.72, 119.2 (d JC-F = 24.2 Hz),, 110.3(d JC-F = 22 Hz), 42.31, 32.32, 26.94, 26.40.The analytical data are consistent with published ones.[12]

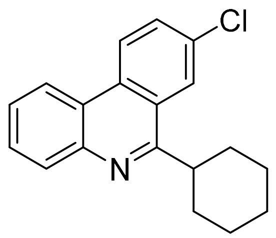

8-choloro-6-cyclohexylphenanthridine (3u): Following the general procedure GP1, two reactions of **2c** (0.1 mmol, 21.3 mg each one) with **1a** (0.15 mmol, 40.9 mg each one) and after 24 hours, the reaction mixture was concentrated under reduced pressure. The product **3u** was purified by flash chromatography on silica (hexane:AcOEt 95:5, followed by hexane:AcOEt 90:10) afforded as a white solid (31.3 mg, 53% yield). ^{1}H NMR (400 MHz, $CDCl_3$) δ 8.58 (d, J = 9.0 Hz, 1H), 8.49-8.47 (m, 1H), 8.25 (d, J = 2 Hz, 1H), 8.13-8.12 (m, 1H), 7.76 (dd, J = 9.0, 2.5 Hz, 1H), 7.73-7.61 (m, 1H), 7.63-7.60 (m, 1H), 3.54-3.48 (m, 1H), 2.06-1.91 (m, 8H), 1.62-1.40 (m, 2H). ^{13}C NMR (100 MHz, $CDCl_3$) δ 164.4, 143.9, 133.2, 131.9, 130.2, 128.9, 128.3, 126.7, 126.2, 124.6, 122.9, 121.8, 121.4, 42.0, 32.4, 26.9, 26.4. The analytical data are consistent with published ones.[12]

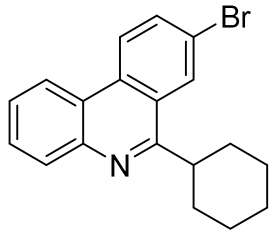

8-bromo-6-cyclohexylphenanthridine (3v): Following the general procedure GP1, two reactions of **2d** (0.1 mmol, 25.8 mg each one) with **1a** (0.15 mmol, 40.9 mg each one) and after 24 hours, the reaction mixture was concentrated under reduced pressure. The product **3v** was purified by flash chromatography on silica (hexane:AcOEt 95:5, followed by hexane:AcOEt 90:10) afforded as a yellow solid (44.0 mg, 65% yield). ^{1}H NMR (400 MHz, $CDCl_3$) δ 8.5 (d, J = 9.1 Hz, 1H), 8.48 (dd, J = 8.0 Hz, 1.5 Hz 1H), 8.41 (d, J = 2.0 Hz, 1H), 7.9 (dd, J = 9.0 Hz, 2.5 Hz, 1H), 7.73-7.70 (m, 1H), 7.62-7.59 (m, 1H), 3.53-3.48 (s, 1H), 2.06-1.86 (m, 6H), 1.62-1.39 (m, 4H). ^{13}C NMR (100 MHz, $CDCl_3$) δ 164.3, 143.9, 133.2, 131.9, 130.2, 128.9, 128.3, 126.7, 126.2, 124.6, 122.9, 121.8, 121.4, 42.0, 32.4, 26.9, 26.4. HRMS (ESI): m/z calcd for $C_{19}H_{19}BrN$ (M+H)+ : 340.0695, found: 340.0710.

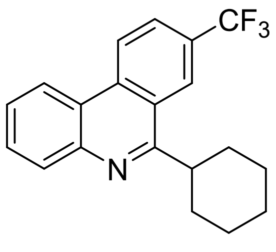

6-cyclohexyl-8-(trifluoromethyl)phenanthridine (3w): Following the general procedure GP1, two reactions of **2e** (0.1 mmol, 24.7 mg each one) with **1a** (0.15 mmol, 40.9 mg each one) and after 24 hours, the reaction mixture was concentrated under reduced pressure. The product **3w** was purified by flash chromatography on silica (hexane:AcOEt 95:5, followed by hexane:AcOEt 90:10) afforded as a white solid (36.2 mg, 55% yield). ^{1}H NMR (400 MHz, $CDCl_3$) 8.75 (d, J = 8.8 Hz, 2H), 8.55-8.54 (m, 2H), 8.16 (dd, J = 8.8, 2.2 Hz, 1H), 8.0 (dd, J = 8.6, 2.1 Hz, 1H), 7.77 (td, J = 8.4, 1.2 Hz, 1H), 7.65 (td, J = 8.4, 1.2 Hz, 1H), 3.63-3.58 (m, 1H), 2.07-1.85 (m, 7H), 1.64-1.57(m, 2H), 1.45-1.42 (m, 1H). ^{13}C NMR (100 MHz, $CDCl_3$) δ 165.2, 144.7, 135.4, 130.3, 129.7, 129.15(q, JC-F = 32.4 Hz), 126.8, 125.9 (q, JC-F = 2.2 Hz), 124.6 (q, JC-F = 270 Hz), 124.2, 123.8, 122.1 (q, JC-F = 4 Hz), 122.5, 122.3, 42.1, 32.5, 26.9, 26.37. ^{19}F NMR (377 MHz, $CDCl_3$): δ -62.07, The analytical data are consistent with published ones.[12]

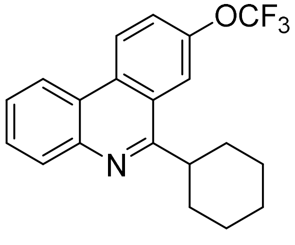

6-cyclohexyl-8-(trifluoromethoxy)phenanthridine (3x): Following the general procedure GP1, two reactions of **2f** (0.1 mmol, 26.3 mg each one) with **1a** (0.15 mmol, 40.9 mg each one) and after 24 hours, the reaction mixture was concentrated under reduced pressure. The product **3x** was purified by flash chromatography on silica (hexane:AcOEt 95:5, followed by hexane:AcOEt 90:10) afforded as a white solid (39.3 mg, 57% yield); and analytical data are consistent with published ones.[2] ^{1}H NMR (400 MHz, $CDCl_3$) 8.69 (d, J = 8.4 Hz, 1H), 8.5 (d, J = 8.4 Hz, 1H), 8.14 (d, J = 8.4 Hz, 1H), 8.09 (s, 1H), 7.74-7.61 (m, 3H), 3.52-3.47 (m, 1H), 2.07-1.87 (m, 8H), 1.62-1.60 (m 1H), 1.47-1.40 (m, 1H). ^{13}C NMR (100 MHz, $CDCl_3$) δ 164.6, 147.9, 144.1, 131.7, 130.3, 129.0, 126.8, 125.7, 124.9, 123.4, 122.7, 121.9, 120.6 (q, JCF = 256 Hz), 117.2, 42.3, 32.4, 26.9, 26.4. ^{19}F NMR (377 MHz, $CDCl_3$): δ -57.65. HRMS (ESI): m/z calcd for $C_{20}H_{19}F_3NO$ (M+H)+ : 346.1413, found: 346.1430.

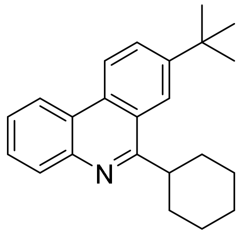

8-(tert-butyl)-6-cyclohexylphenanthridine (3y): Following the general procedure **GP1**, two reactions of **2g** (0.1 mmol, 23.5 mg each one) with **1a** (0.15 mmol, 40.9 mg each one) and after 24 hours, the reaction mixture was concentrated under reduced pressure. The product **3y** was purified by flash chromatography on silica (hexane:AcOEt 95:5, followed by hexane:AcOEt 90:10) afforded as white solid in 72% yield (45.6 mg). ^{1}H NMR (400 MHz, $CDCl_3$) 8.6 (d, J = 8.6 Hz, 1H), 8.50 (dd, J = 8.0 Hz, 0.8 Hz, 1H), 8.27 (d, J = 2.0 Hz, 1H), 8.11 (dd, J = 8.0, 0.8 Hz, 1H), 7.89 (dd, J = 8.4, 2.0 Hz, 1H), 7.68-7.65 (m, 1H), 7.59-7.56 (m, 1H), 3.59-3.56 (m, 1H), 2.11-1.84 (m, 7H), 1.64-1.58 (m, 2H), 1.48 (s, 9H), 1.45-1.42 (m, 1H). ^{13}C NMR (100 MHz, $CDCl_3$) 165.43, 150.05, 143.81, 130.93, 129.96, 128.35, 128.08, 126.15, 124.69, 123.49, 122.51, 121.82, 121.18, 42.35, 35.24, 32.45, 31.51, 27.06, 26.52. HRMS (ESI): m/z calcd for $C_{23}H_{28}N$ (M+H)+ : 318.2216, found: 318.2219.

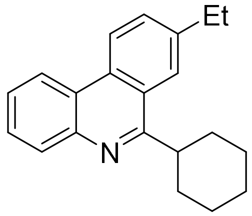

6-cyclohexyl-8-ethylphenanthridine (3z): Following the general procedure GP1, two reactions of **2h** (0.1 mmol, 20.7 mg each one) with **1a** (0.15 mmol, 40.9 mg each one) and after 24 hours, the reaction mixture was concentrated under reduced pressure. The product **3z** was purified by flash chromatography on silica (hexane:AcOEt 95:5, followed by hexane:AcOEt 90:10) afforded as a white solid (38.8 mg, 75% yield). ^{1}H NMR (400 MHz, $CDCl_3$) δ 8.56 (d, J = 8.2 Hz , 1H), 8.50 (dd, J = 8.2, 0.8Hz, 1H), 8.12 (dd, J = 8.1, 1.2 Hz, 1H), 8.08-8.07 (m, 1H), 7.69-7.65 (m, 2H), 7.60-7.56 (m, 1H), 3.65-3.58 (m, 1H), 2.91 (q, J = 7.6 Hz , 2H), 2.10-1.93 (m, 7H), 1.64-1.58 (m, 3H), 1.39 (t, J = 7.6 Hz, 3H). ^{13}C NMR: (100 MHz, $CDCl_3$) δ 165.2, 143.7, 143.3, 131.2, 130.6, 129.9, 128.05, 126.1, 125.0, 124.0, 123.5, 122.7, 121.7, 42.0, 32.4, 29.4, 27.0, 26.4, 15.9. HRMS (ESI): m/z calcd for $C_{21}H_{24}N$ $(M+H)^+$: 290.1903, found: 250.1906.

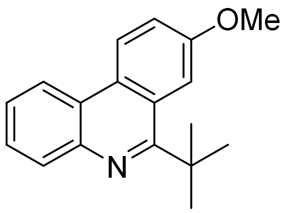

6-(tert-butyl)-8-methoxyphenanthridine (3aa): Following the general procedure GP1, two reactions of **2i** (0.1 mmol, 20.9 mg each one) with **1i** (0.15 mmol, 37.05 mg each one) and after 24 hours, the reaction mixture was concentrated under reduced pressure. The product **3aa** was purified by flash chromatography on silica (hexane:AcOEt 95:5, followed by hexane:AcOEt 90:10) afforded as a yellow solid (38.7 mg, 73% yield). ^{1}H NMR (400 MHz, $CDCl_3$) δ 8.59 (d, J = 9.1 Hz, 1H), 8.44 (d, J = 8.0 Hz, 1H), 8.10 (d, J = 8.0 Hz, 1H), 7.99 (d, J = 2.5 Hz, 1H), 7.65-7.56 (m, 2H), 7.44 (dd, J = 9.0, 2.0 Hz, 1H), 4.00 (s, 3H), 1.75 (s, 9H). ^{13}C NMR (100 MHz, $CDCl_3$) δ 165.7, 157.2, 142.2, 130.2, 128.2, 127.4, 126.5, 125.5, 124.4, 123.5, 121.1, 119.1, 109.7, 55.5, 40.0, 31.0. HRMS (ESI): m/z calcd for $C_{18}H_{20}NO$ $(M+H)^+$: 266.1539, found: 266.1546. The analytical data are consistent with published ones.[24]

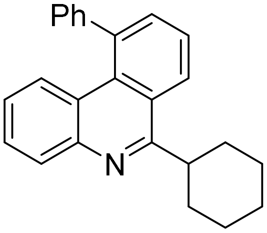

6-cyclohexyl-10-phenylphenanthridine (3ab): Following the general procedure GP1, two reactions of **2j** (0.1 mmol, 25.5 mg each one) with **1a** (0.15 mmol, 40.9 mg each one) and after 24 hours, the reaction mixture was concentrated under reduced pressure. The product **3ab** was purified by flash chromatography on silica (hexane:AcOEt 95:5, followed by hexane:AcOEt 90:10) afforded as a white solid (39 mg, 58% yield). ^{1}H NMR (400 MHz, $CDCl_3$) δ 8.34 (d, J = 7.2 Hz, 1H), 8.06 (d, J = 7.6 Hz, 1H), 7.69 (t, J = 8.0 Hz, 1H), 7.63-7.61 (m, 1H), 7.53-7.46 (m, 5H), 7.41-7.39 (m, 2H), 7.06-7.03 (m, 1H), 2.13-2.10 (m, 2H), 2.01-1.85 (m, 5H), 1.63-1.42 (m, 1H). ^{13}C NMR (100 MHz, $CDCl_3$) δ 165.1, 145.3, 144.5, 140.6, 133.7, 131.3, 129.9, 129.2 (2C), 129.1 (2C), 127.9, 127.6, 127.3, 126.3, 126.2, 125.2, 124.6, 123.5, 42.5, 32.6, 27.1, 26.5. HRMS (ESI): m/z calcd for $C_{25}H_{24}N$ $(M+H)^+$: 338.1903, found: 338.1909.

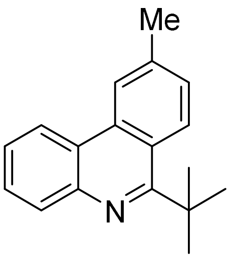

6-(tert-butyl)-9-methylphenanthridine (3ac): Following the general procedure GP1, two reactions of **2k** (0.2 mmol, 38.6 mg each one) with **1i** (0.3 mmol,74 mg each one) and after 24 hours, the reaction mixture was concentrated under reduced pressure. The product **3ac** was purified by flash chromatography on silica (hexane:AcOEt 95:5, followed by hexane:AcOEt 90:10) afforded as a yellowish oil (65.7 mg, 66% yield). The analytical data are consistent with published ones.^{1}H NMR (400 MHz, $CDCl_3$) δ 8.53 (s, 1H), 8.51 (s, 1H), 8.47 (s, 1H), 8.11 (d, J = 2.5 Hz, 1H), 7.68 (dt, J = 1.2, 7.4 Hz, 1H), 7.59 (dt, J = 7.6, 2.0 Hz, 1H),7.47 (dd, J = 7.6, 1.2 Hz, 1H), 2.63 (s, 3H), 1.72 (s, 9H); ^{13}C NMR (100 MHz, $CDCl_3$) δ 166.6, 143.2, 139.4, 134.3, 130.3, 128.3, 128.2, 127.7, 126.3, 123.4, 122.7, 122.5, 121.7, 40.24, 31.3, 22.1. HRMS (ESI): m/z calcd for $C_{18}H_{20}N$ $(M+H)^+$: 250.1590, found: 250.1596.

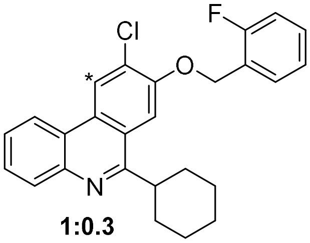

1:0.3

9-chloro-6-cyclohexyl-8-((2-fluorobenzyl)oxy)phenanthridines (3ad): Following the general procedure GP1, two reactions of **2l** (0.1 mmol, 33.8 mg each one) with **1a** (0.15 mmol, 40.9 mg each one) and after 24 hours, the reaction mixture was concentrated under reduced pressure. The product **3ad** was purified by flash chromatography on silica (hexane:AcOEt 90:10, followed by hexane:AcOEt 80:20) afforded as a yellowish solid (54.5 mg, 65% yield). The analytical data are consistent with published ones.[10] Major isomer: ^{1}H NMR (400 MHz, $CDCl_3$) δ 8.63 (s, 1H), 8.36 (d, J = 8.6 Hz, 1H), 8.08 (dd, J = 2.8 Hz, 8.8 Hz, 1H), 7.68-7.54 (m, 5H), 7.36-7.30 (m, 1H), 7.20-7.13 (m, 2H), 5.47 (s, 2H), 3.4-3.33 (m, 1H), 1.96-1.84 (m, 7H), 1.57-1.38 (m, 3H). ^{13}C NMR (100 MHz, $CDCl_3$): both major and minor isomer: δ 164.01, 161.69, 159.24, 153.93, 152.50, 143.53, 142.53, 130.21, 130.13, 130.07, 129.97, 129.62, 129.58, 128.31, 128.22, 127.96, 126.67, 126.57, 124.77, 124.74, 124.63, 124.38, 123.38, 123.24, 122.53, 122.37, 121.57, 121.51, 120.76, 117.28, 115.65, 115.44,

108.43, 65.70, 64.50, 45.09, 42.40, 33.45, 32.20, 27.11, 26.93, 26.40. ^{19}F NMR (377 MHz, $CDCl_3$): major isomer; δ -118.48, minor isomer; δ -118.65. HRMS (ESI): m/z calcd for $C_{26}H_{24}ClFNO$ (M+H)+ : 420.1525, found: 420.1539.

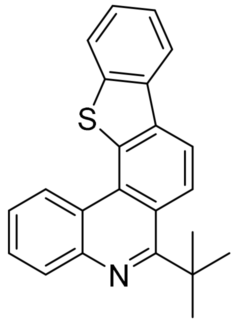

6-(tert-butyl)benzo[4,5]thieno[3,2-*k*]phenanthridine (**3ae**): Following the general procedure **GP1**, two reactions of **2m** (0.1 mmol, 28.6 mg each one) with **1i** (0.15 mmol, 37 mg each one) and after 24 hours, the reaction mixture was concentrated under reduced pressure. The product **3ae** was purified by flash chromatography on silica (hexane:AcOEt 95:5, followed by hexane:AcOEt 90:10) afforded as a yellow solid in 57% yield (38.8 mg).^{1}H NMR (400 MHz, $CDCl_3$) 9.19-9.16 (m, 1H), 8.82 (d, J = 8.8 Hz),), 8.47 (d, J = 8.8 Hz), 8.36-8.34 (m, 1H), 8.28-8.26 (m, 1H), 8.07-8.05 (m, 1H), 7.83-7.79 (m, 2H), 7.60-7.58 (m, 2H), 1.82 (s, 9H). ^{13}C NMR (100 MHz, $CDCl_3$) δ 166.9, 144.1, 140.4, 136.6, 135.01, 134.6, 130.9 (2C), 128.4, 127.5, 126.8, 125.2, 125.1 (2C), 124.3, 123.2, 122.4, 122.2, 119.5, 40.6, 31.7. HRMS (ESI): m/z calcd for $C_{23}H_{20}NS$ (M+H)+ : 342.1311, found: 342.1313.

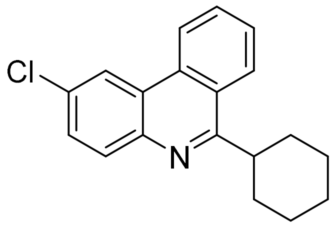

3,8-dimethoxy-6*H*-benzo[*c*]chromen-6-one (3af): Following the general procedure **GP1**, two reactions of **2n** (0.2 mmol, 42.8 mg each one) with **1a** (0.3 mmol, 81.9 mg each one) and after 24 hours, the reaction mixture was concentrated under reduced pressure. The product **3af** was purified by flash chromatography on silica (hexane:AcOEt 95:5, followed by hexane:AcOEt 90:10) afforded **3af** as a yellow oil in 71% yield (83.8 mg). ^{1}H NMR (500 MHz, $CDCl_3$) δ 8.55 (d, J = 8.4 Hz, 1H), 8.48 (s, 1H), 8.31 (d, J = 8.4 Hz, 1H), 8.05 (d, J = 8

Hz, 1H), 7.82 (t, J = 7.2 1H), 7.71 (t, J = 7.6 Hz, 1H), 7.62 (t, J = 8.4 Hz, 1H), 3.62-3.56 (m, 1H), 2.07-1.83 (m, 7H), 1.55-1.40 (m, 3H). ^{13}C NMR (125 MHz, $CDCl_3$): δ 165.78, 142.45, 132.16, 132.02, 131.55, 130.35, 128.96, 127.88, 125.84, 124.99, 124.58, 122.75, 121.62, 42.14, 32.40, 26.98, 26.44. The analytical data are consistent with published ones.[2

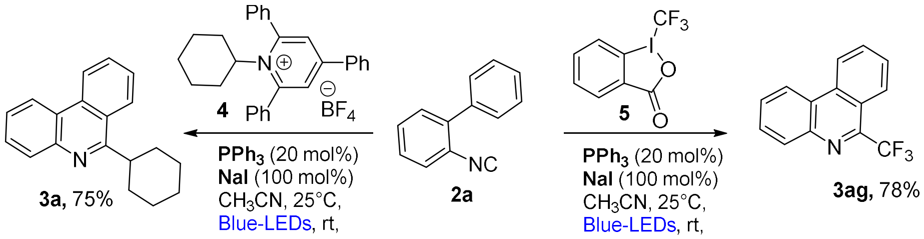

6-(trifluoromethyl)phenanthridine (3ag): Following the general procedure **GP1**, two reactions of **2a** (0.1 mmol, 17.9 mg each one) with **5** (0.15 mmol, 47.2 mg each one) and after 24 hours, the reaction mixture was concentrated under reduced pressure. The product **3ag** was purified by flash chromatography on silica (hexane:AcOEt 95:5, followed by hexane:AcOEt 90:10) afforded as pale yellow solid (38.5 mg, 78% yield); ^{1}H NMR (400 MHz, $CDCl_3$) δ 8.70 (d, J = 8.5 Hz, 1H), 8.62−8.61 (m, 1H), 8.40−8.38 (m, 1H), 8.30−8.29 (m, 1H), 7.95−7.91 (m, 1H), 7.84−7.76 (m, 3H); ^{13}C NMR (125 MHz, CDCl3) δ 146.5 (q, JC−F = 32.8 Hz), 141.8, 134.1, 131.5, 131.3, 129.4, 129.3, 128.2, 126.0 (q, JC−F = 3.3 Hz), 125.2, 122.6, 122.2, 122.0 (q, JC−F = 275.5 Hz), 121.9; ^{19}F NMR (377 MHz, $CDCl_3$) δ −63.5 (s, 3F); The analytical data are consistent with published ones.[26]

6-cyclohexylphenanthridine (3a): Following the general procedure **GP1**, two reactions of **2a** (0.1 mmol, 17.9 mg each one) with **4** (0.15 mmol. 71.5 mg each one) afforded **3a** as yellowish oil (39 mg, 75% yield). The analytical data are consistent with published ones. [12]

Mechanistic studies

ON/OFF experiment: A 10 mL glass vial was charged with alkyl NHP-ester (**1a**, 0.3 mmol), 2-isocyano biphenyl (**2a**, 0.2 mmol), PPh_3(20 mol%), NaI (100 mol%), trimethoxybenzene (0.2 mmol, internal standard) and a PTFE-coated stirring bar. The glass vial was sealed with a PTFE septum and dry degassed CH_3CN solvent (3 mL) was added to the reaction vial. The reactions were placed in a pre-programed temperature (25°C) controlled blue LED reactor (as shown in **Figure 1**) and the reaction mixture was irradiated with a 455 nm blue LED. After the selected time has expired, a small aliquot was removed and concentrated under reduced pressure, analyzed by ^{1}H NMR to determine the yield.

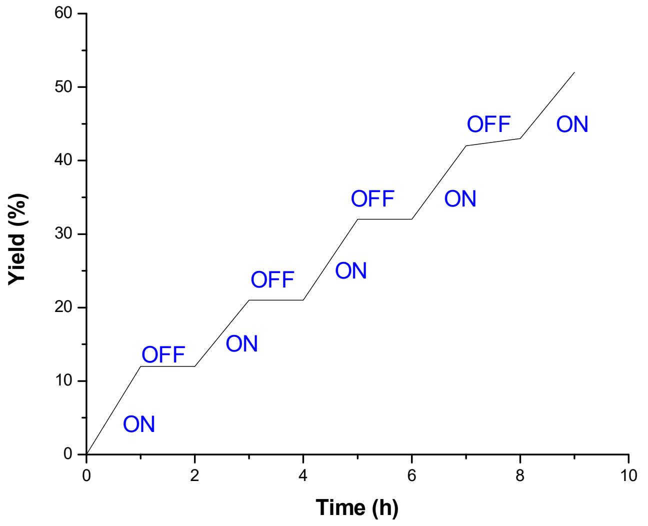

Figure 3. *The above reaction profile upon the alternating irradiation shows that the reaction can only proceed in presence of light, whereas the catalytic activity is inhibited under darkness, thus confirming the previous results from the conditions screening.*

Radical clock experiment

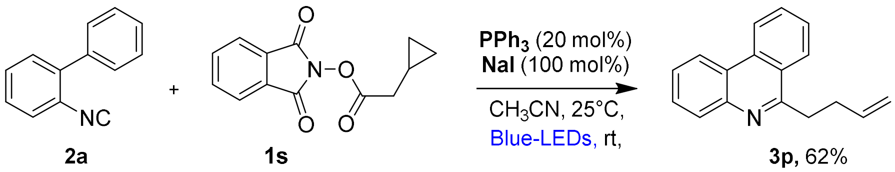

6-(but-3-en-1-yl)phenanthridine (3p): Following the general procedure **GP1**, two reactions of **2a** (0.2 mmol, 35.8 mg each one) with **1s** (0.3 mmol, 73.5 mg each one) afforded **3p** as yellow solid in 62% yield (57.8 mg). ^{1}H NMR: (400 MHz, $CDCl_3$) δ 8.65 (d, J = 8.2 Hz , 1H), 8.55 (dd, J = 8.2, 1.3 Hz, 1H), 8.25 (d, J = 8.1 Hz, 1H), 8.13 (dd, J = 8.1 Hz, 1.1 Hz, 1H), 7.83(td, J = 8.3, 1.3 Hz, 1H), 7.73-7.68 (m, 2H), 7.64-7.60 (m, 1H), 6.10 – 6.0 (m, 1H), 5.20-5.14 (m, 1H), 5.06-5.02 (m, 1H), 3.49 – 3.45 (m, 2H), 2.74 – 2.69 (m, 2H).^{13}C NMR: (100 MHz, $CDCl_3$) δ 161.4, 143.8, 138.1, 133.0, 130.4, 129.7, 128.7, 127.4, 126.5, 126.2, 125.3, 123.7, 122.6, 122.0, 115.2, 35.5, 33.3. The analytical data are consistent with published ones.[22]

Racemization Experiment

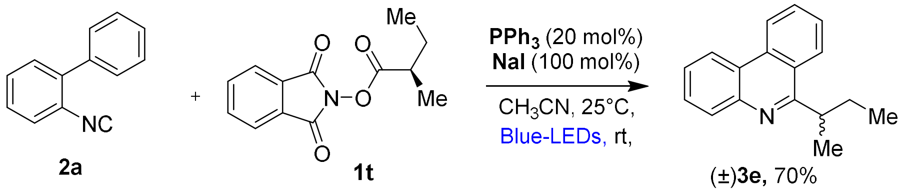

6-(sec-butyl)phenanthridine (3p): Following the general procedure **GP1**, two reactions of **2a** (0.2 mmol, 35.8 mg each one) with **1t** (0.3 mmol, 74 mg each one) afforded **3e** as a colorless oil in 70% yield (65.8 mg). ^{1}H NMR (400 MHz, $CDCl_3$) δ 8.66 (d, J = 8.2 Hz, 1H), 8.55 (d, J = 8.1 Hz, 1H), 8.34 (d, J = 8.3 Hz, 1H), 8.14 (d, J = 8.1 Hz, 1H), 7.84 – 7.80 (m, 1H), 7.72 – 7.68 (m, 2H), 7.63 – 7.60 (m, 1H), 3.80 – 3.73 (m, 1H), 2.22 – 2.13 (m, 1H), 1.87 – 1.78 (m, 1H), 1.49 (d, J = 6.8 Hz, 3H), 0.99 (t, J = 7.4 Hz, 3H). ^{13}C NMR (100 MHz, $CDCl_3$) δ 165.48, 143.89, 133.03, 129.96, 128.41, 127.11, 126.16, 125.68, 125.29, 123.35, 122.59, 121.85, 38.36, 29.21, 19.82, 12.50. The analytical data are consistent with published ones.[15]

Optical rotation of an authentic racemic sample (**3e**) which is obtained from the reaction of **2a** with **1e** according to general procedure (GP1):

$[\alpha]_{589}$ (293 K, $CHCl_3$) = +0.2 degrees·dm^{-1} (10 mg/ 1 ml)

Optical rotation of the reaction sample (±**3e**)

$[\alpha]_{589}$ (293 K, $CHCl_3$) = 0.10 degrees·dm^{-1} (10 mg/ 1 ml)

Within the experimental error, no significant difference in the optical rotation of an independently synthesized sample and the reaction product could be detected. Therefore, it must be concluded that racemization occurs during the reaction.

UV-Vis experiments:

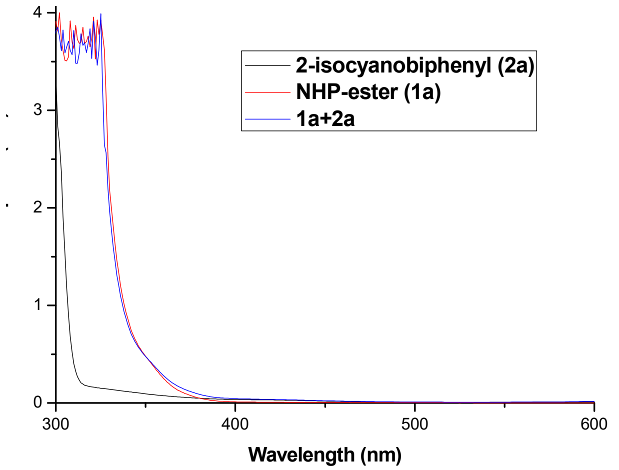

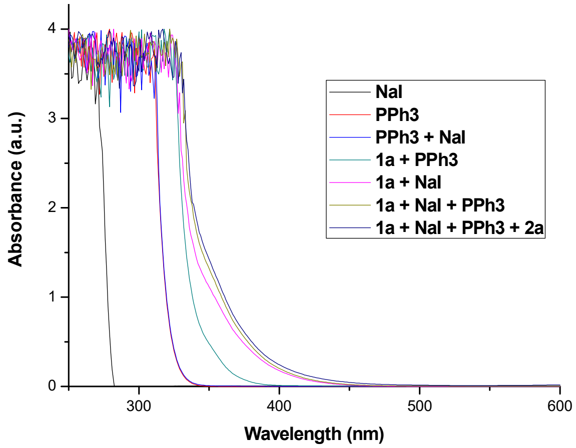

Figure 4: UV/vis absorption spectra of the combination between different starting materials recorded in CH_3CN as solvent.

Further understanding of this PPh_3/NaI photoredox system in our catalysis, we measured UV-visible absorption spectra (Figure 4) using a solution of the same concentration as the reaction mixture, from which it indicated that PPh_3, NaI or the combination of PPh_3 and NaI did not absorb in the visible light region (Figure 4). 2-isocyano biphenyl (**2a**) showed absorption only in the UV (<320 nm). In case of NHP-ester (**1a**) the absorption starts around 390 nm. Compared with the absorption spectra of NHP-ester (**1a**), the absorption spectra of the mixture of NHP-ester (**1a**) with 2-isocyano biphenyl (**2a**) did not show any significant change, which suggests that no intermolecular charge transfer took place. However, a clear red shift of absorption onset, tailing into the wavelength range of blue LED irradiation, was observed when the PPh_3/NaI component was mixed with NHP-ester (**1a**). This red shift supports the formation of a charge-transfer complex between PPh_3/NaI and NHP-ester (**1a**) in the reaction mixture (Figure 4).

NMR spectra

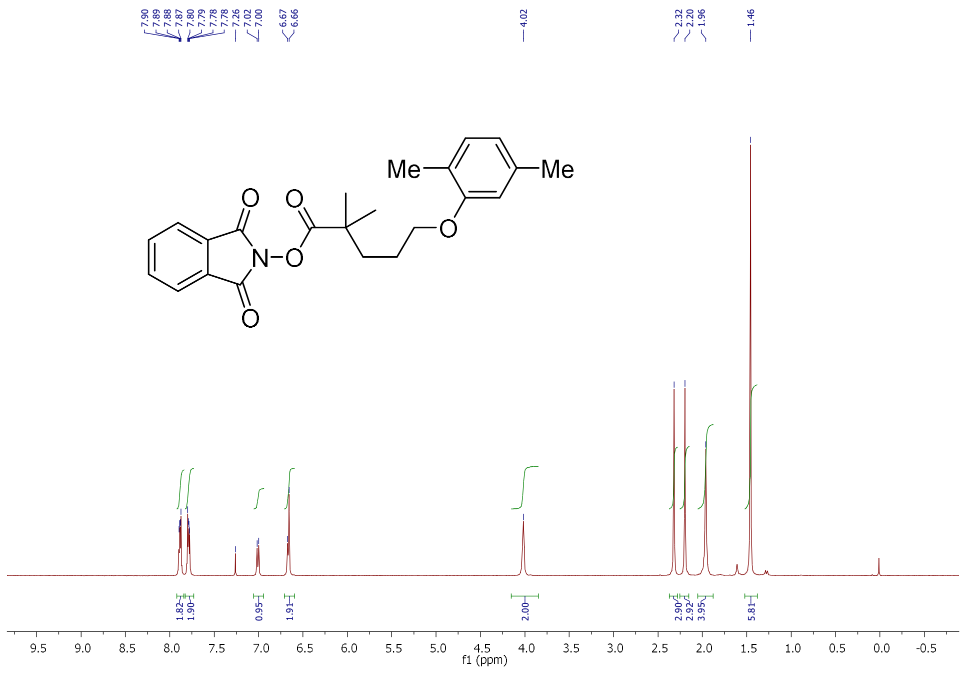
7.90
7.89
7.88
7.87
7.80
7.79
7.78
7.78
7.26
7.02
7.00
6.67
6.66
4.02
2.32
2.20
1.96
1.46
Me
Me
O
O
O
N
O
O
O
1.82
1.90
0.95
1.91
2.00
2.90
2.92
3.95
5.81
f1 (ppm)

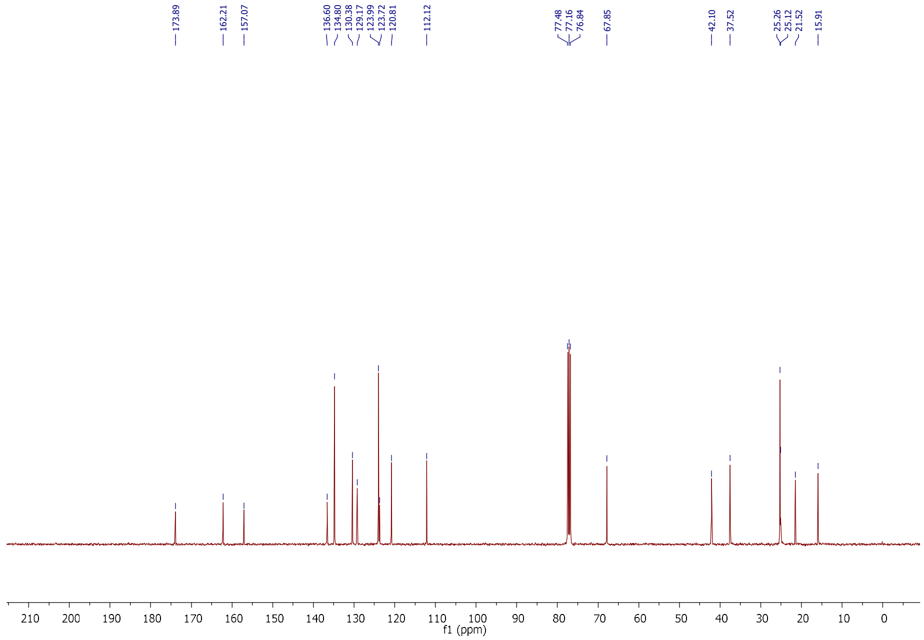
173.89
162.21
157.07
136.60
134.80
130.38
129.17
123.99
123.72
120.81
112.12
77.48
77.16
76.84
67.85
42.10
37.52
25.26
25.12
21.52
15.91
f1 (ppm)

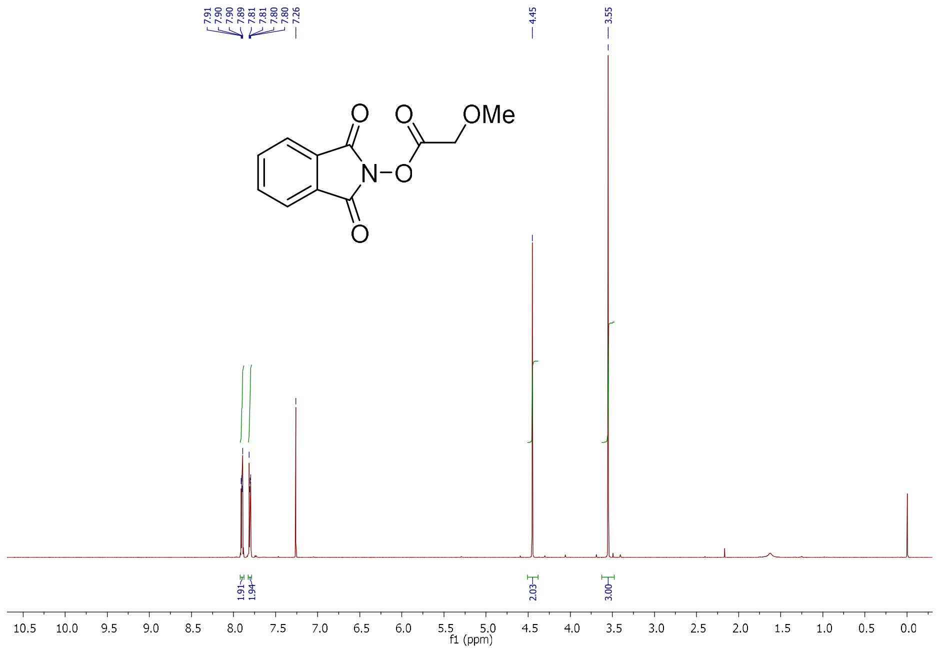
7.91
7.90
7.90
7.89
7.81
7.81
7.80
7.80
7.26
4.45
3.55
OMe
N
O
1.91
1.94
2.03
3.00
10.5 10.0 9.5 9.0 8.5 8.0 7.5 7.0 6.5 6.0 5.5 5.0 4.5 4.0 3.5 3.0 2.5 2.0 1.5 1.0 0.5 0.0
f1 (ppm)

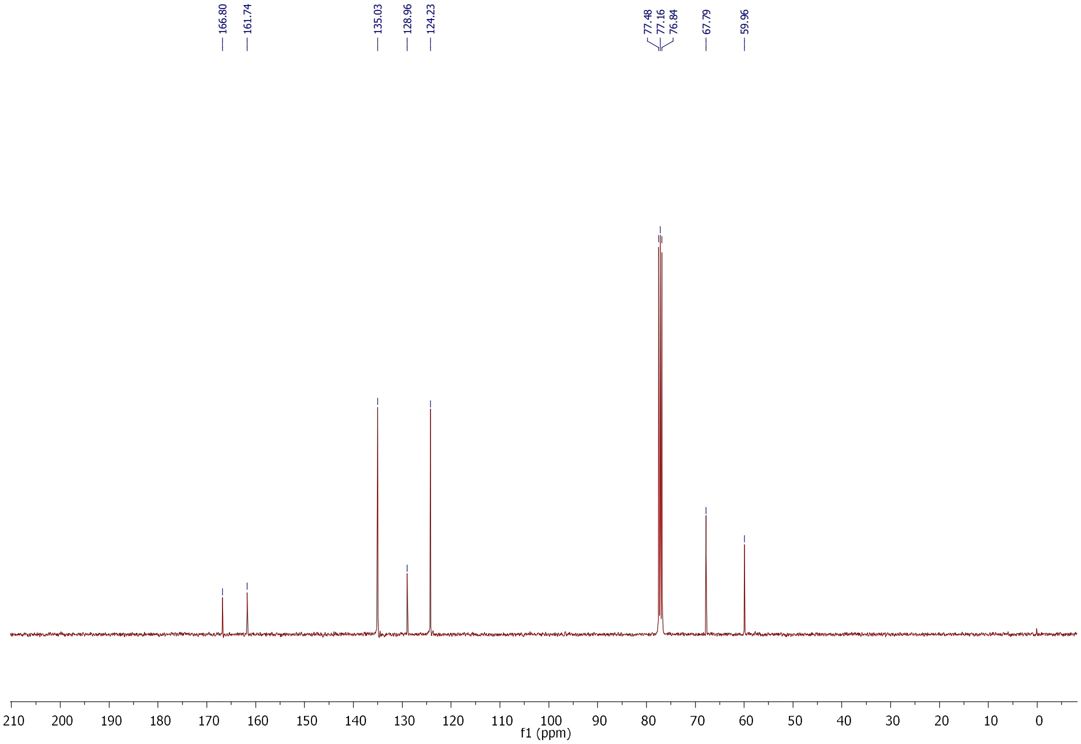
166.80
161.74
135.03
128.96
124.23
77.48
77.16
76.84
67.79
59.96
210 200 190 180 170 160 150 140 130 120 110 100 90 80 70 60 50 40 30 20 10 0
f1 (ppm)

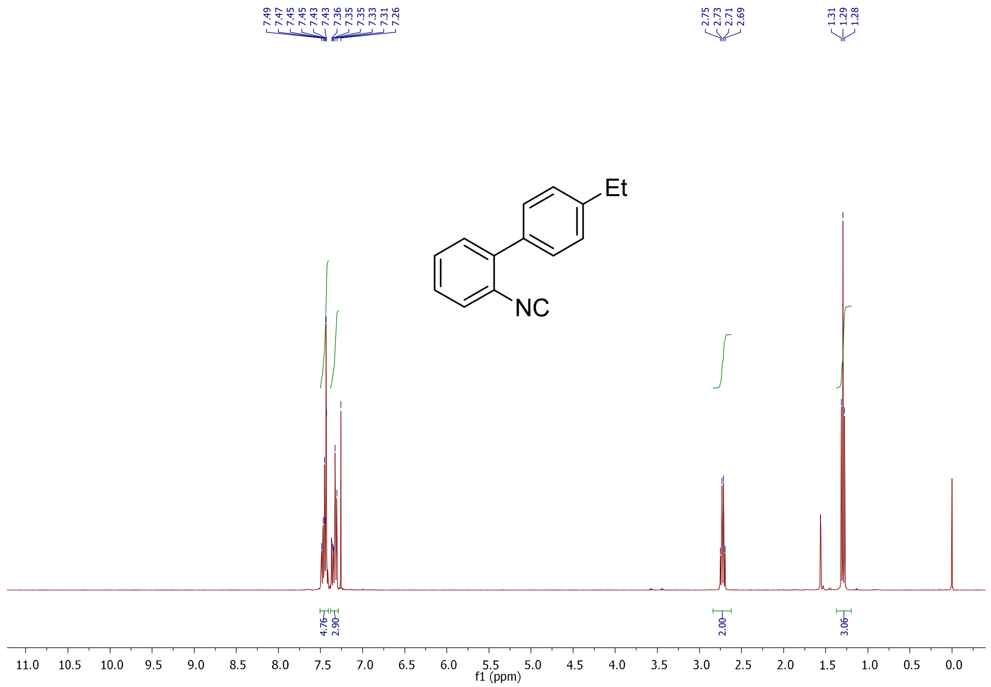

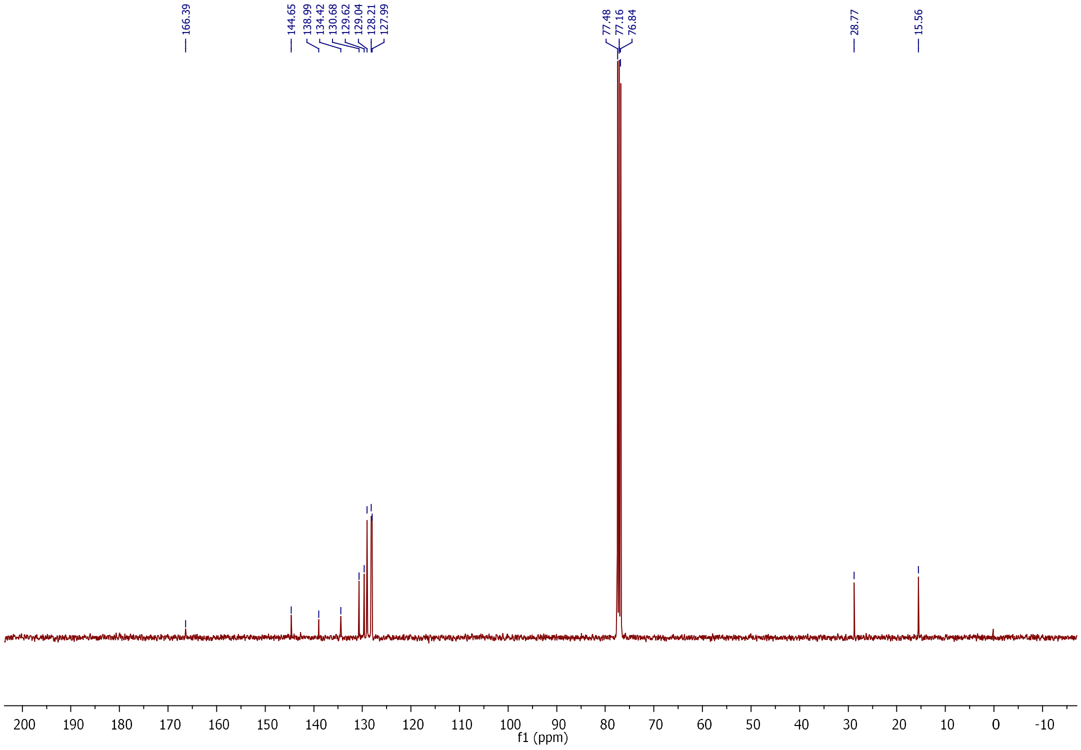

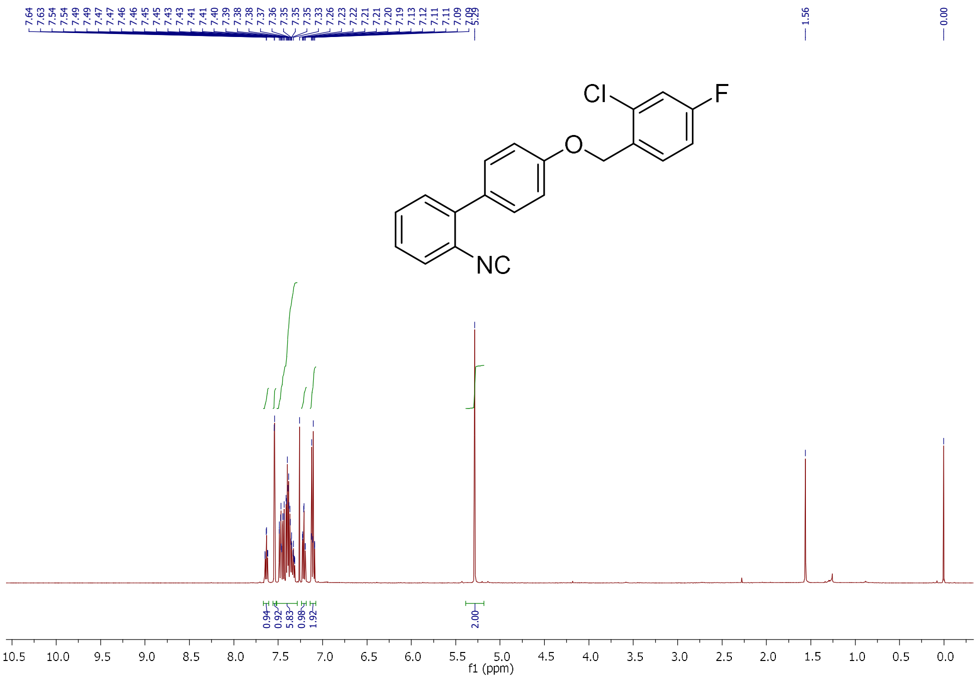

5.29
1.56
0.00
Cl
F
O
NC
0.94
0.92
5.83
0.98
1.92
2.00
f1 (ppm)

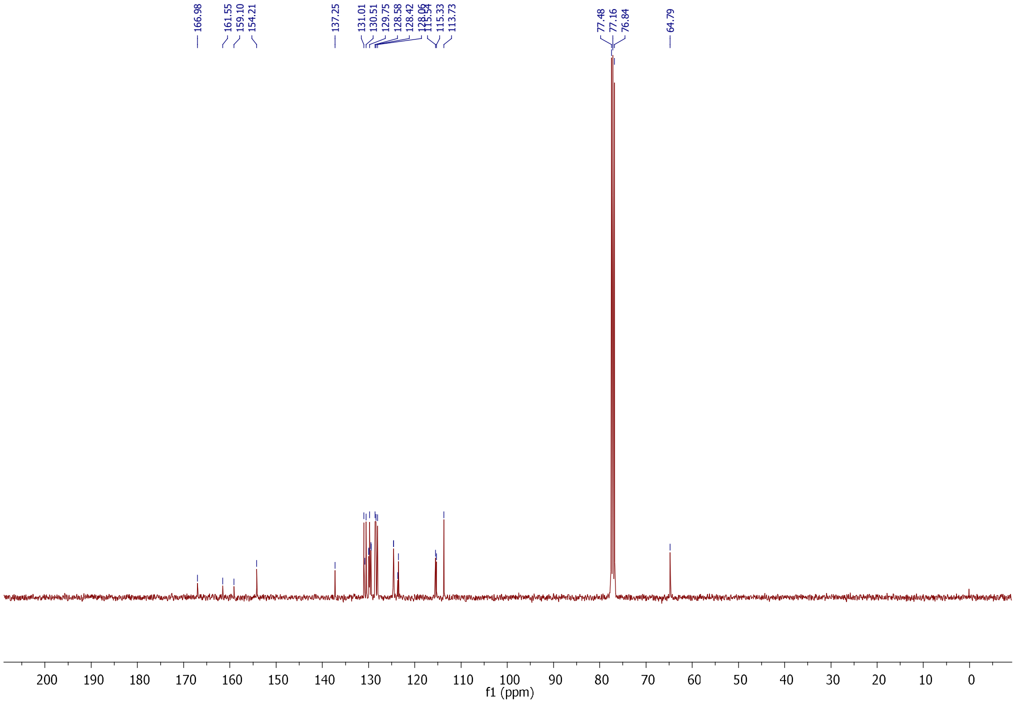

166.98
161.55
159.10
154.21
137.25
131.01
130.51
129.75
128.58
128.42
115.33
113.73
77.48
77.16
76.84
64.79
f1 (ppm)

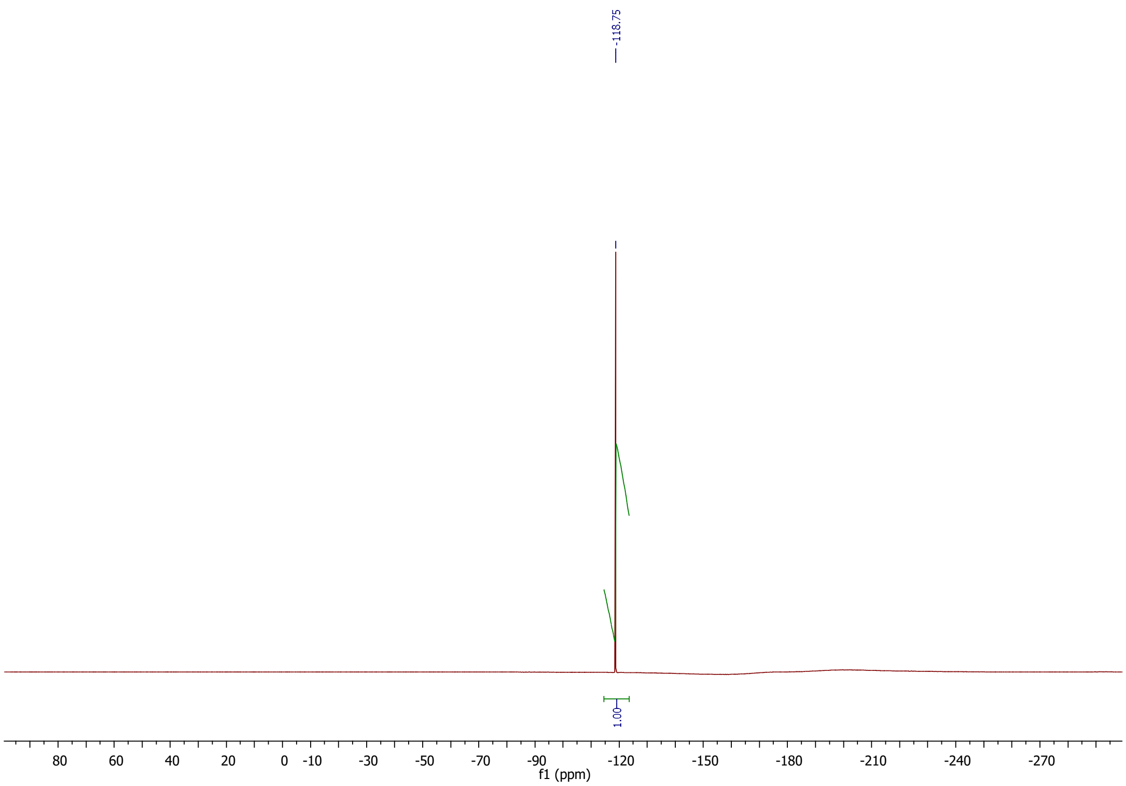
-118.75
1.00
80
60
40
20
0
-10
-30
-50
-70
-90
-120
-150
-180
-210
-240
-270
f1 (ppm)

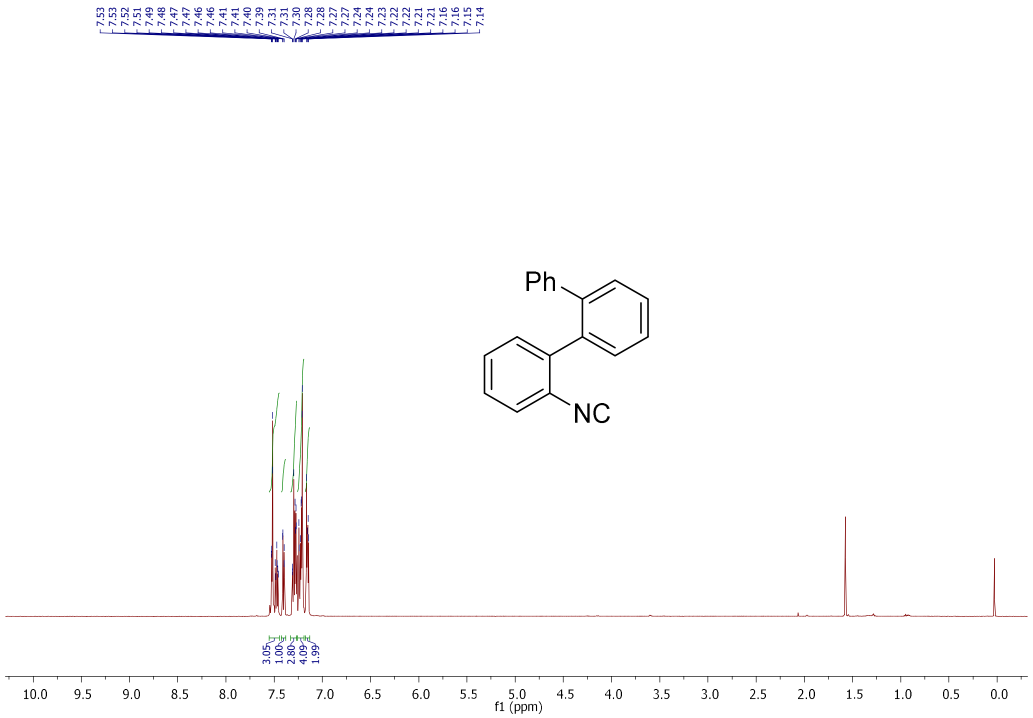
7.53
7.53
7.52
7.51
7.49
7.48
7.47
7.47
7.46
7.46
7.41
7.41
7.40
7.39
7.31
7.31
7.30
7.28
7.27
7.27
7.24
7.24
7.23
7.22
7.22
7.21
7.16
7.15
7.14
Ph
NC
3.05
1.00
2.80
4.09
1.99
10.0 9.5 9.0 8.5 8.0 7.5 7.0 6.5 6.0 5.5 5.0 4.5 4.0 3.5 3.0 2.5 2.0 1.5 1.0 0.5 0.0
f1 (ppm)

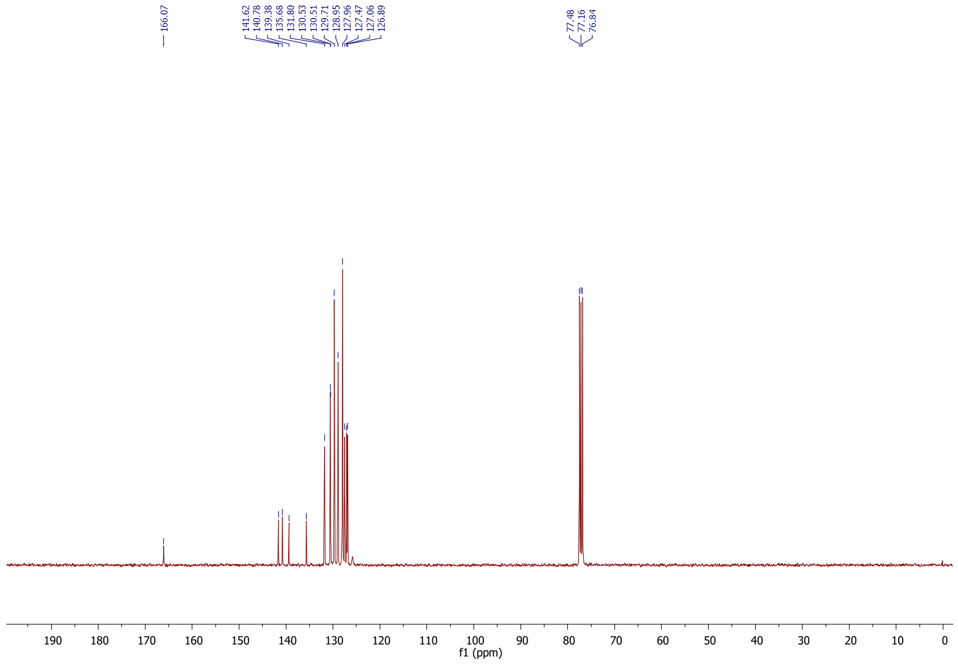
166.07
141.62
140.78
139.38
135.68
131.80
130.53
130.51
129.71
128.95
127.96
127.47
127.06
126.89
77.48
77.16
76.84
190 180 170 160 150 140 130 120 110 100 90 80 70 60 50 40 30 20 10 0
f1 (ppm)

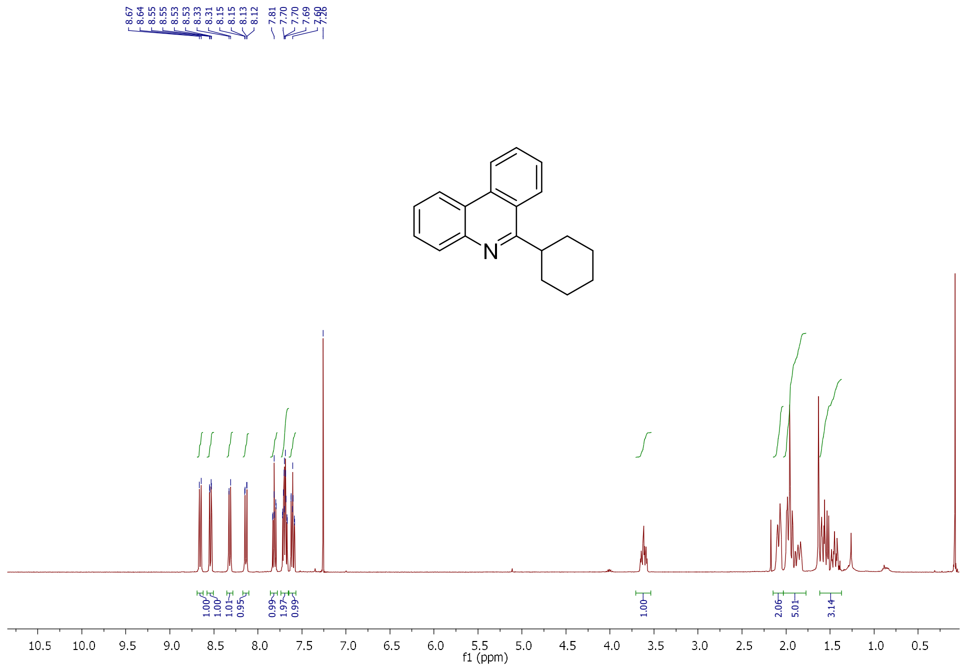
8.67
8.64
8.55
8.55
8.53
8.53
8.33
8.31
8.15
8.15
8.13
8.12
7.81
7.70
7.70
7.69
7.60
7.58
N
1.00
1.00
1.01
0.95
0.99
1.97
0.99
1.00
2.06
5.01
3.14
f1 (ppm)

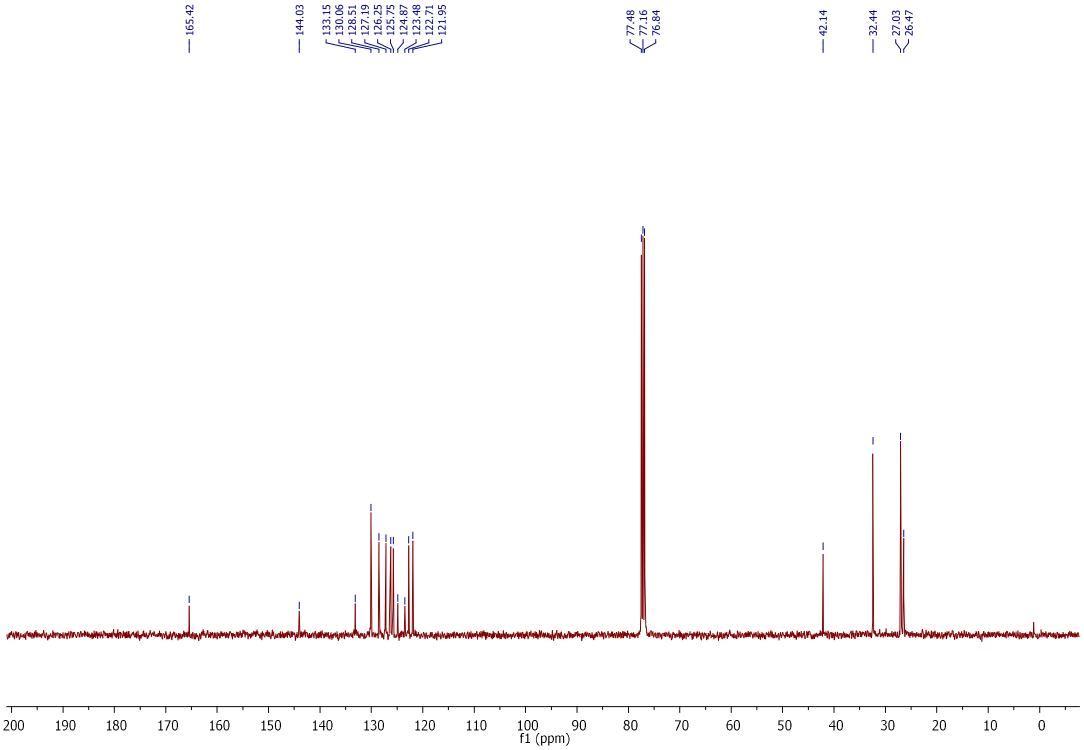
165.42
144.03
133.15
130.06
128.51
127.19
126.25
125.75
124.87
123.48
122.71
121.95
77.48
77.16
76.84
42.14
32.44
27.03
26.47
f1 (ppm)

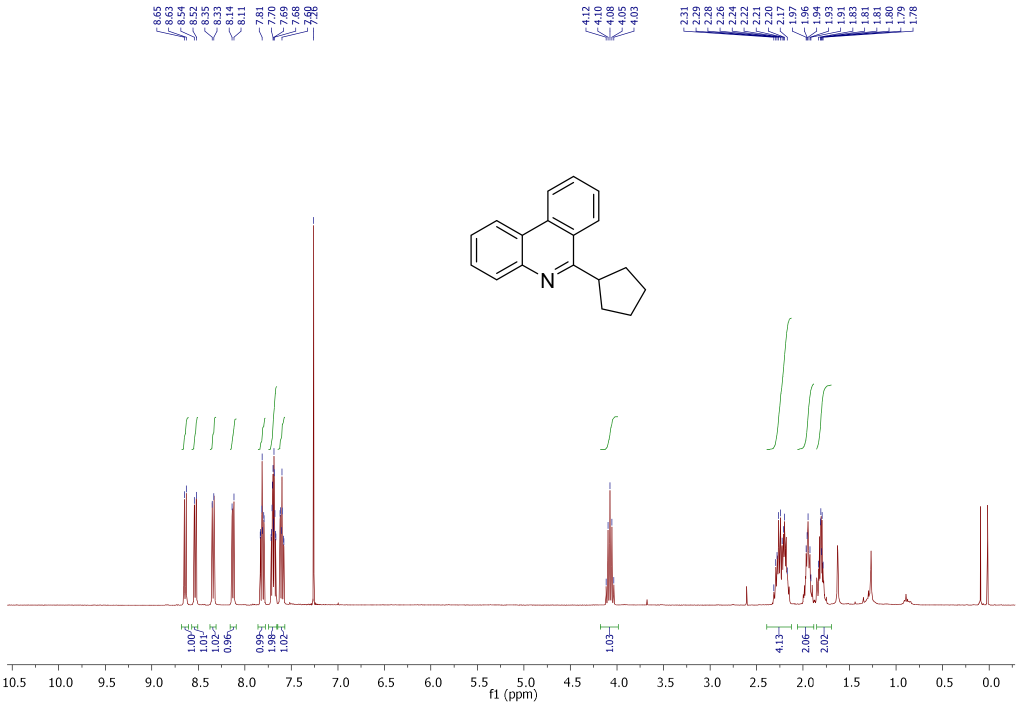

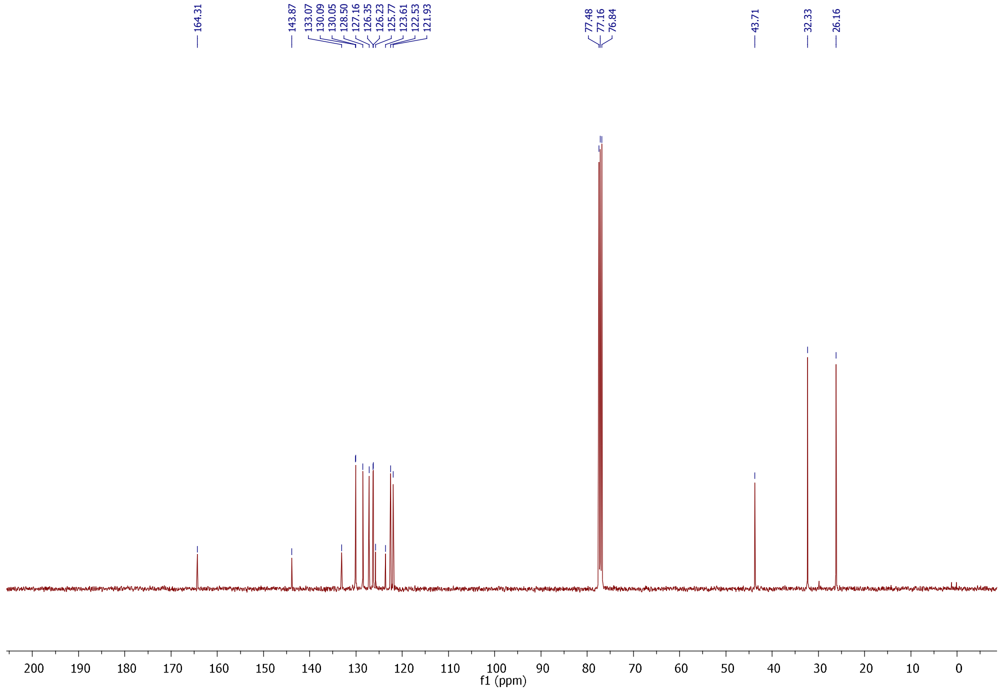

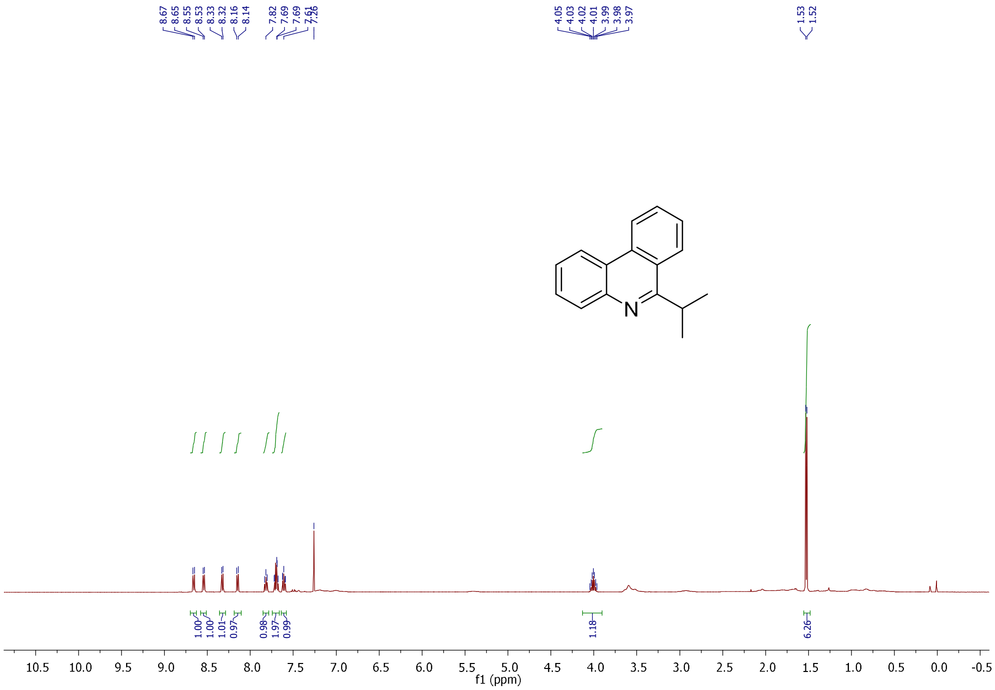
8.67
8.65
8.55
8.53
8.33
8.32
8.16
8.14
7.82
7.69
7.69
7.61
7.26
4.05
4.03
4.02
4.01
3.99
3.98
3.97
1.53
1.52
N
1.00
1.00
1.01
0.97
0.98
1.97
0.99
1.18
6.26
10.5 10.0 9.5 9.0 8.5 8.0 7.5 7.0 6.5 6.0 5.5 5.0 4.5 4.0 3.5 3.0 2.5 2.0 1.5 1.0 0.5 0.0 -0.5
f1 (ppm)

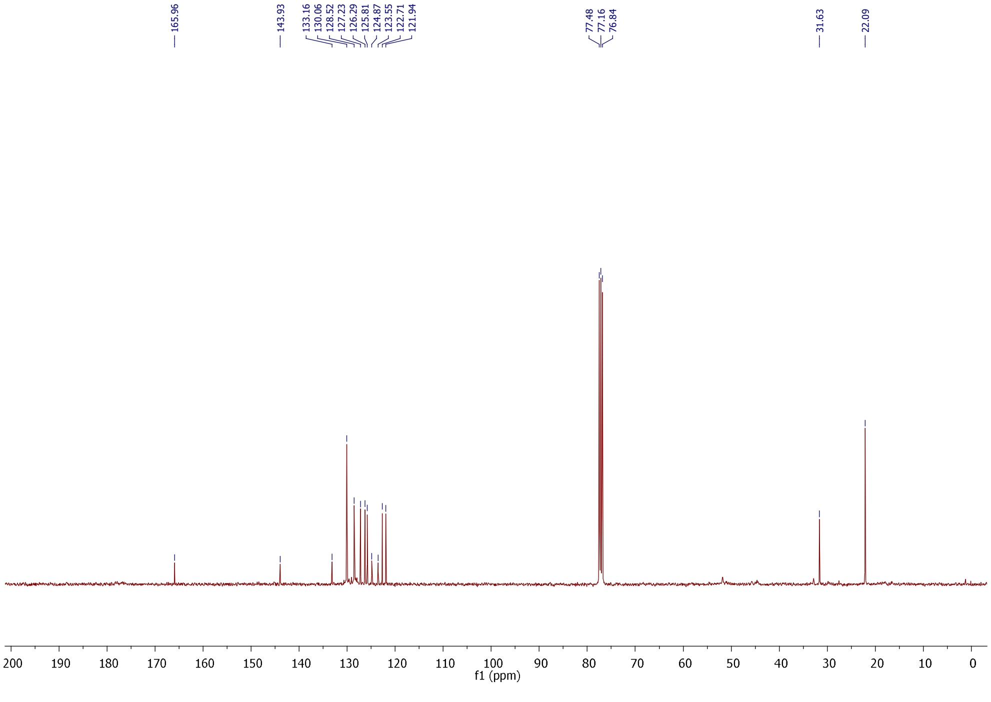
165.96
143.93
133.16
130.06
128.52
127.23
126.29
125.81
124.87
123.55
122.71
121.94
77.48
77.16
76.84
31.63
22.09
200 190 180 170 160 150 140 130 120 110 100 90 80 70 60 50 40 30 20 10 0
f1 (ppm)

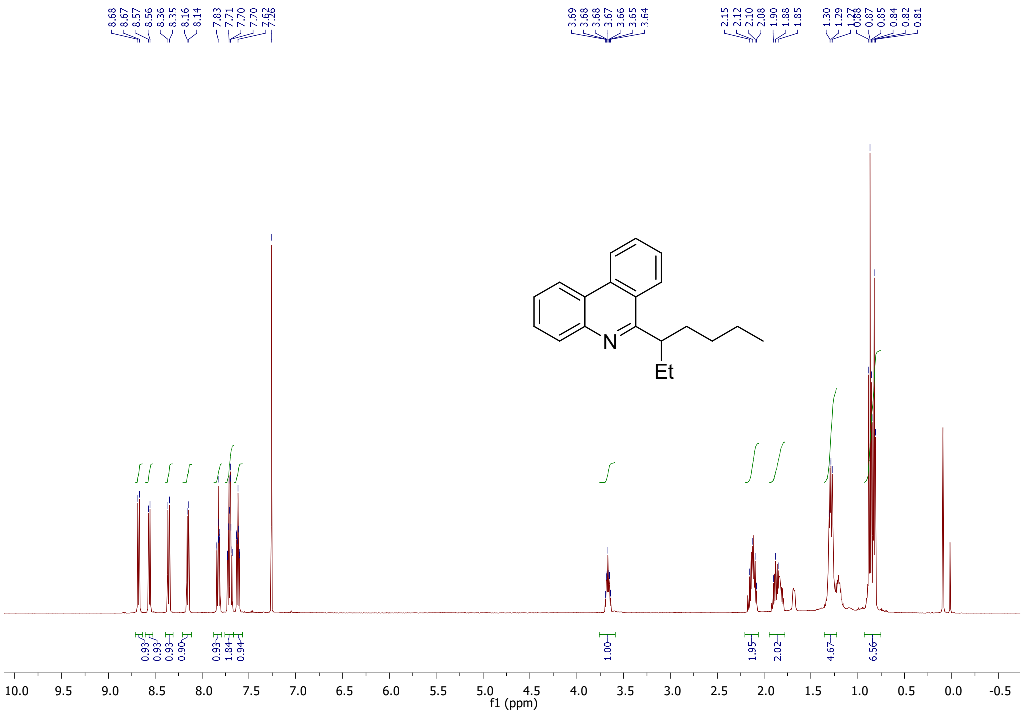

8.68
8.67
8.57
8.56
8.36
8.35
8.16
8.14
7.83
7.71
7.70
7.70
7.62
7.26
3.69
3.68
3.68
3.67
3.66
3.65
3.64
2.15
2.12
2.10
2.08
1.90
1.88
1.85
1.30
1.29
1.27
0.88
0.87
0.85
0.84
0.82
0.81
N
Et
0.93
0.93
0.93
0.90
0.93
1.84
0.94
1.00
1.95
2.02
4.67
6.56
10.0 9.5 9.0 8.5 8.0 7.5 7.0 6.5 6.0 5.5 5.0 4.5 4.0 3.5 3.0 2.5 2.0 1.5 1.0 0.5 0.0 -0.5
f1 (ppm)

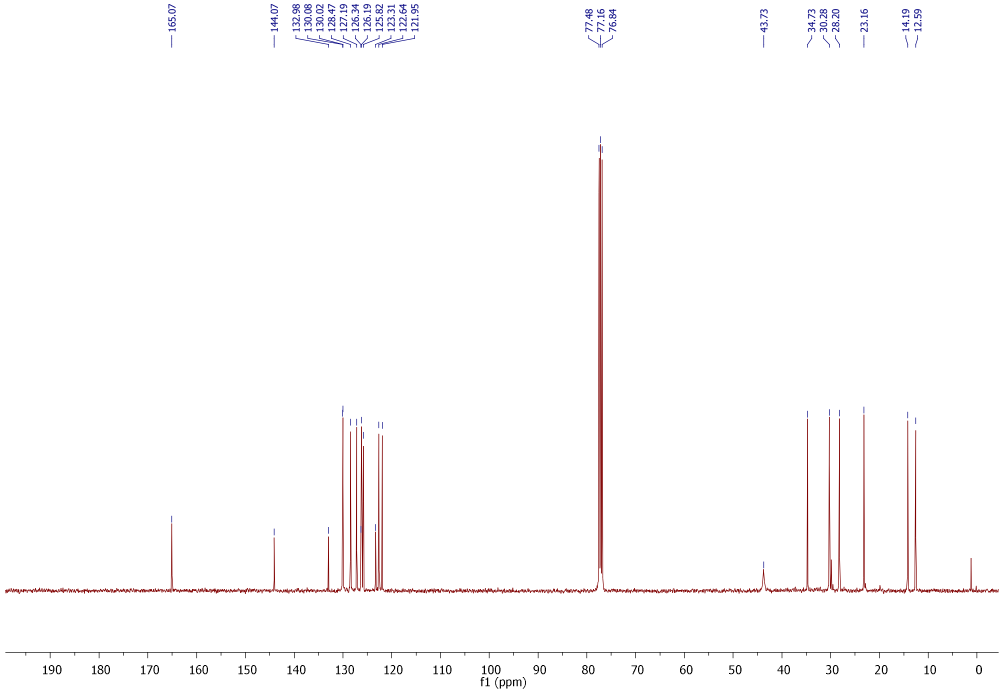

165.07
144.07
132.98
130.08
130.02
128.47
127.19
126.34
126.19
125.82
123.31
122.64
121.95
77.48
77.16
76.84
43.73
34.73
30.28
28.20
23.16
14.19
12.59
190 180 170 160 150 140 130 120 110 100 90 80 70 60 50 40 30 20 10 0
f1 (ppm)

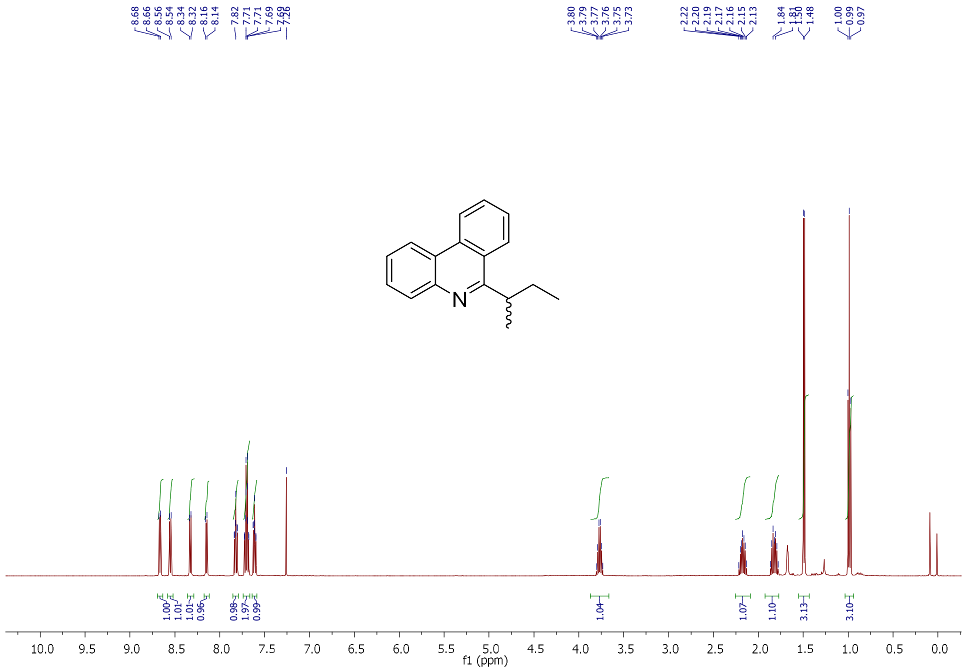
8.68
8.66
8.56
8.54
8.34
8.32
8.16
8.14
7.82
7.71
7.71
7.69
3.80
3.79
3.77
3.76
3.75
3.73
2.22
2.20
2.19
2.17
2.16
2.15
2.13
1.84
1.48
1.00
0.99
0.97
N
1.00
1.01
1.01
0.96
0.98
1.97
0.99
1.04
1.07
1.10
3.13
3.10
10.0 9.5 9.0 8.5 8.0 7.5 7.0 6.5 6.0 5.5 5.0 4.5 4.0 3.5 3.0 2.5 2.0 1.5 1.0 0.5 0.0
f1 (ppm)

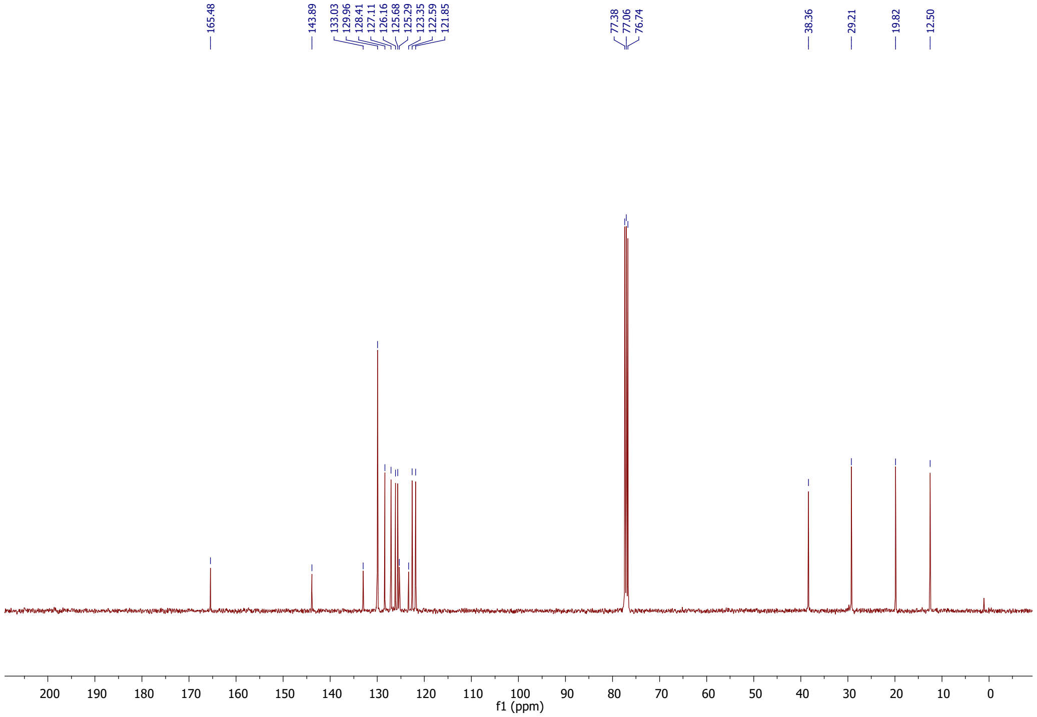
165.48
143.89
133.03
129.96
128.41
127.11
126.16
125.68
125.29
123.35
122.59
121.85
77.38
77.06
76.74
38.36
29.21
19.82
12.50
200 190 180 170 160 150 140 130 120 110 100 90 80 70 60 50 40 30 20 10 0
f1 (ppm)

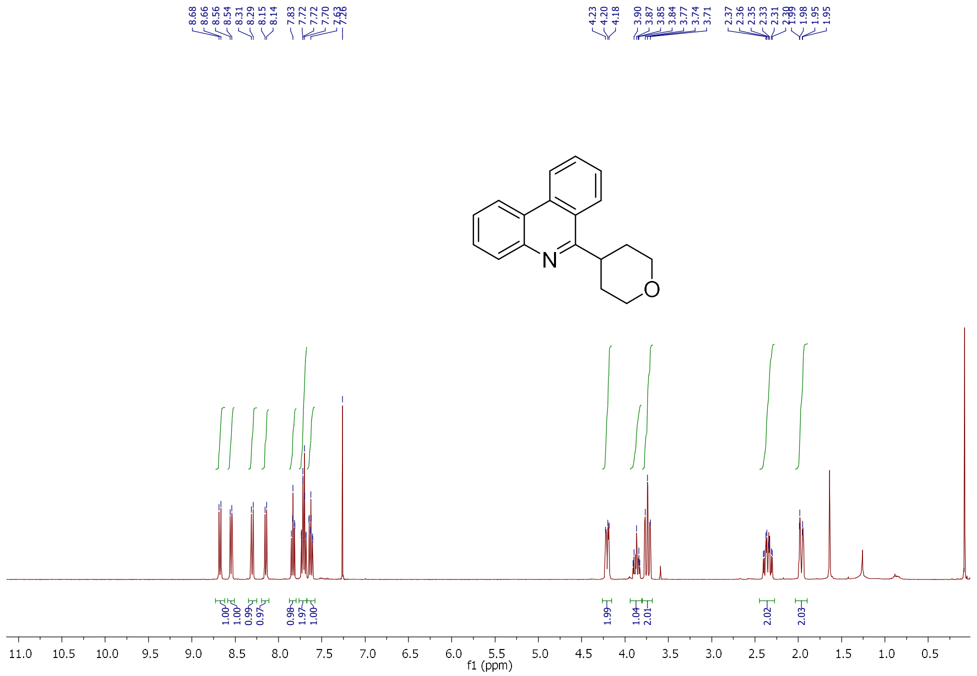
8.68
8.66
8.56
8.54
8.31
8.29
8.15
8.14
7.83
7.72
7.72
7.70
7.26
4.23
4.20
4.18
3.90
3.87
3.85
3.84
3.77
3.74
3.71
2.37
2.36
2.35
2.33
2.31
1.98
1.95
1.95
N
O
1.00
1.00
0.99
0.97
0.98
1.97
1.00
1.99
1.04
2.01
2.02
2.03
11.0 10.5 10.0 9.5 9.0 8.5 8.0 7.5 7.0 6.5 6.0 5.5 5.0 4.5 4.0 3.5 3.0 2.5 2.0 1.5 1.0 0.5
f1 (ppm)

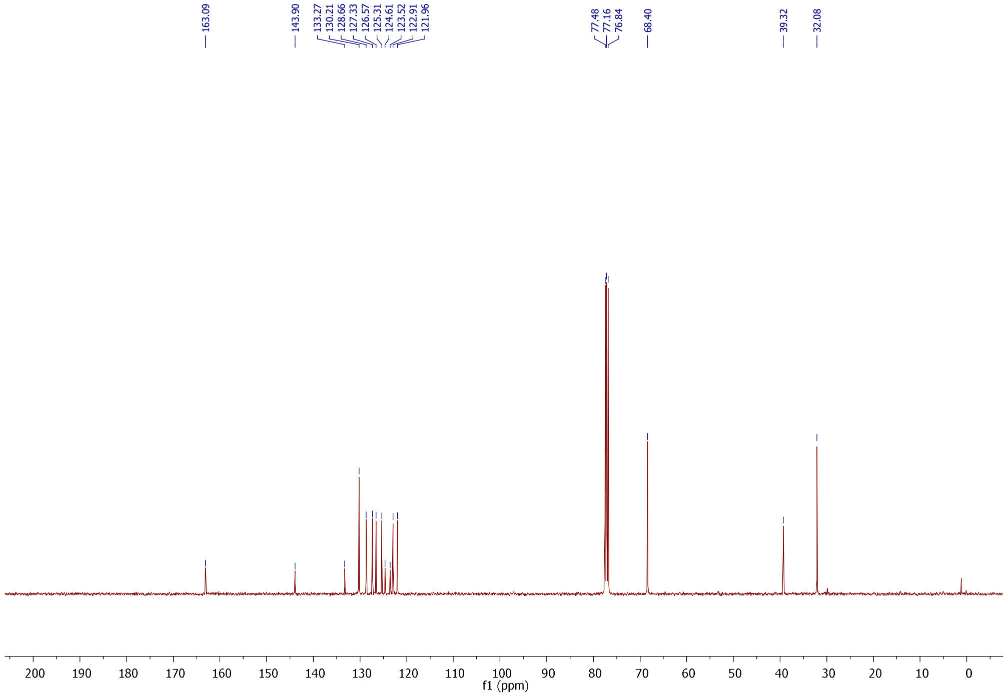
163.09
143.90
133.27
130.21
128.66
127.33
126.57
125.31
124.61
123.52
122.91
121.96
77.48
77.16
76.84
68.40
39.32
32.08
200 190 180 170 160 150 140 130 120 110 100 90 80 70 60 50 40 30 20 10 0
f1 (ppm)

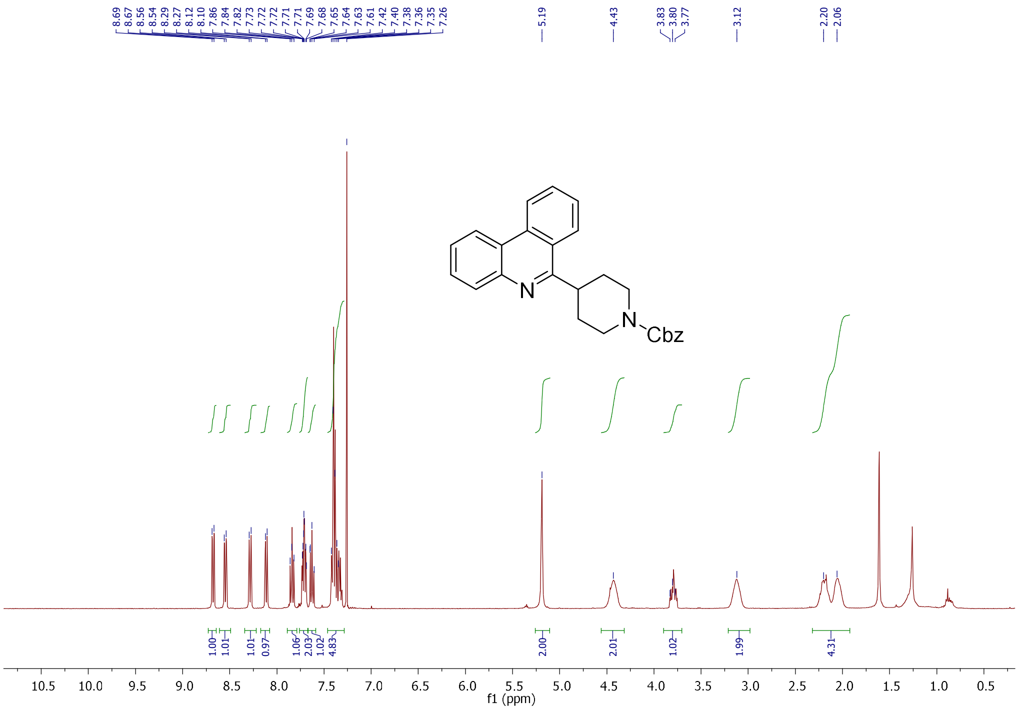
8.69 8.67 8.56 8.54 8.29 8.27 8.12 8.10 7.86 7.84 7.82 7.73 7.72 7.72 7.71 7.71 7.69 7.68 7.65 7.64 7.63 7.61 7.42 7.40 7.38 7.36 7.35 7.26
5.19
4.43
3.83 3.80 3.77
3.12
2.20 2.06
N
Cbz
1.00 1.01 1.01 0.97 1.06 2.03 1.02 4.83
2.00
2.01
1.02
1.99
4.31
10.5 10.0 9.5 9.0 8.5 8.0 7.5 7.0 6.5 6.0 5.5 5.0 4.5 4.0 3.5 3.0 2.5 2.0 1.5 1.0 0.5
f1 (ppm)

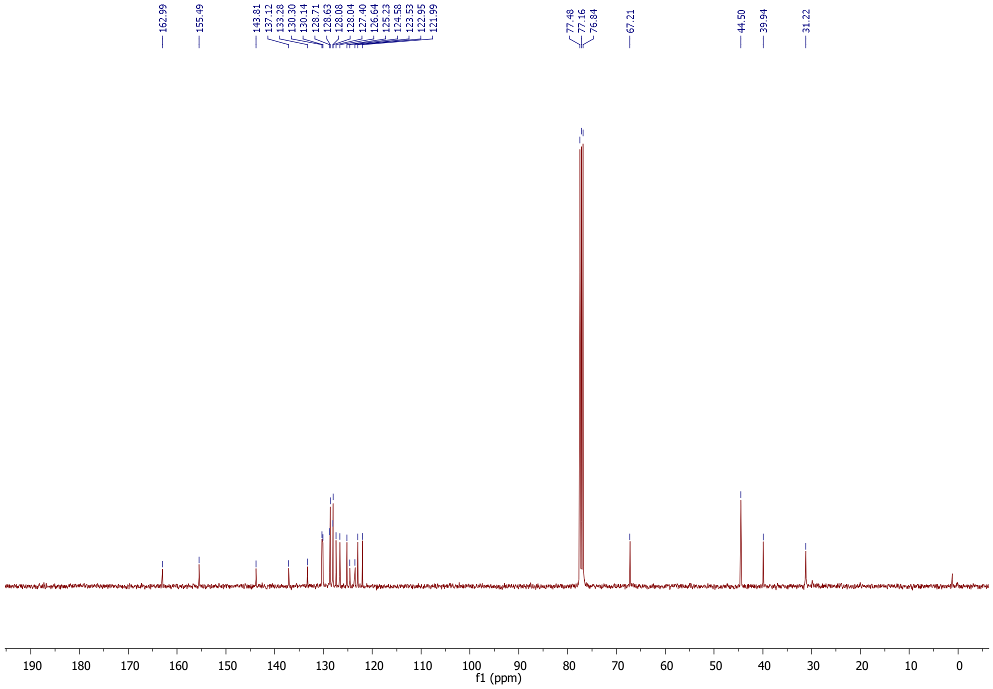
162.99
155.49
143.81 137.12 133.28 130.30 130.14 128.71 128.63 128.08 128.04 127.40 126.64 125.23 124.58 123.53 122.95 121.99
77.48 77.16 76.84
67.21
44.50
39.94
31.22
190 180 170 160 150 140 130 120 110 100 90 80 70 60 50 40 30 20 10 0
f1 (ppm)

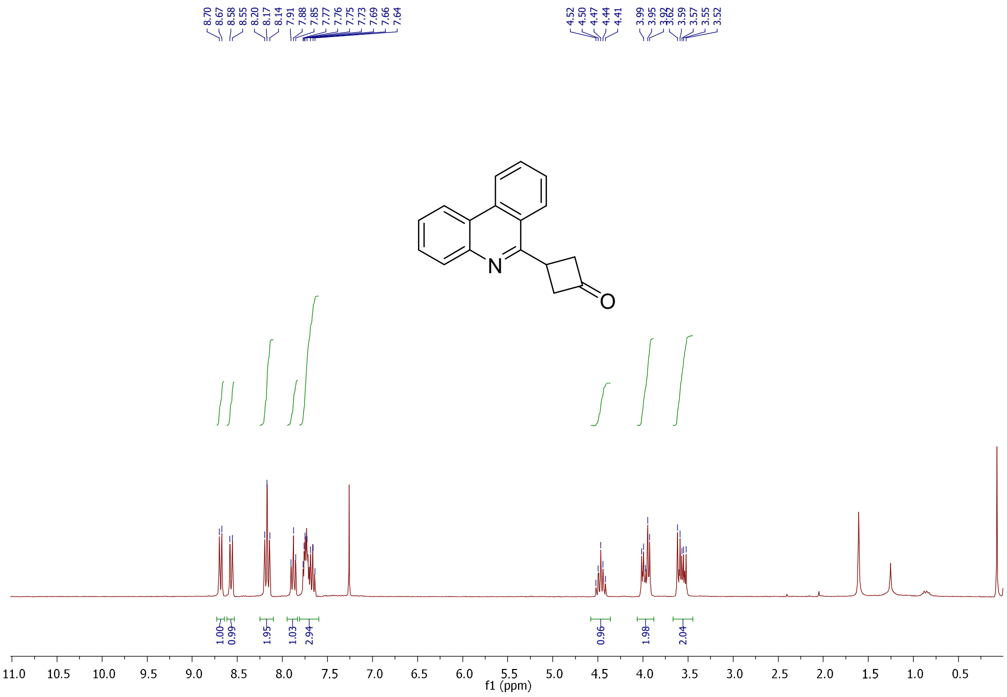
8.70
8.67
8.58
8.55
8.20
8.17
8.14
7.91
7.88
7.85
7.77
7.76
7.75
7.73
7.69
7.66
7.64
4.52
4.50
4.47
4.44
4.41
3.99
3.95
3.92
3.62
3.59
3.57
3.55
3.52
N
O
1.00
0.99
1.95
1.03
2.94
0.96
1.98
2.04
f1 (ppm)

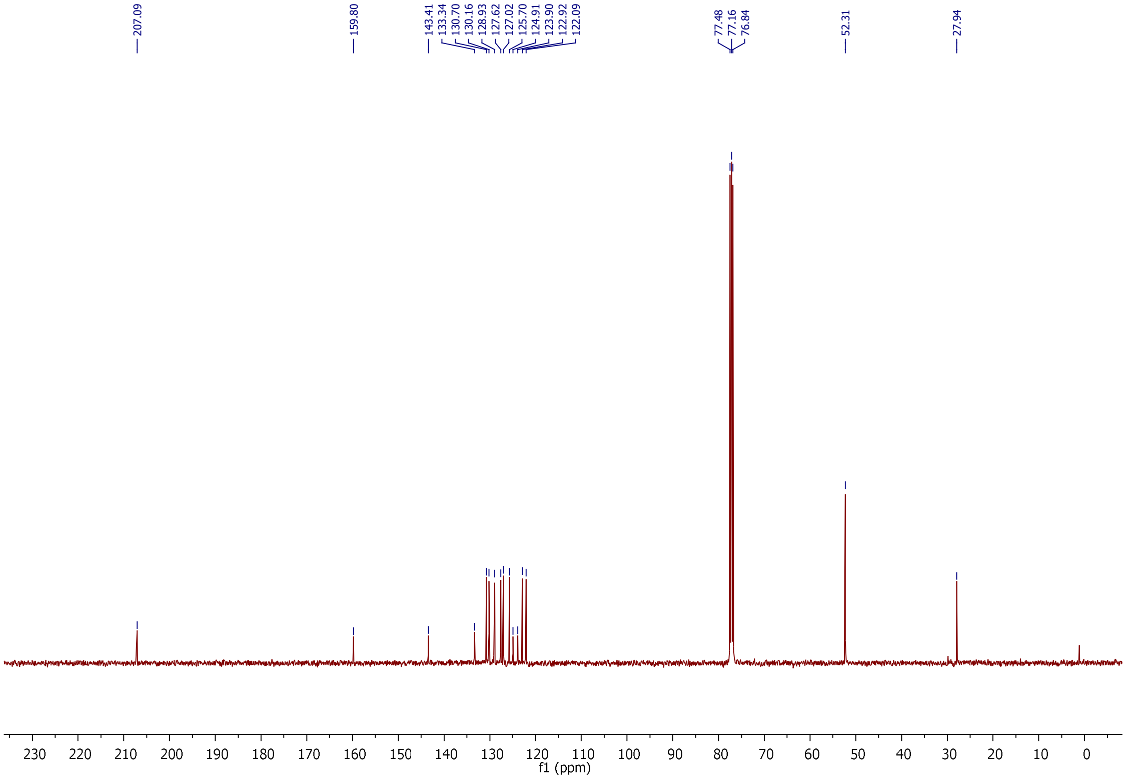
207.09
159.80
143.41
133.34
130.70
130.16
128.93
127.62
127.02
125.70
124.91
123.90
122.92
122.09
77.48
77.16
76.84
52.31
27.94
f1 (ppm)

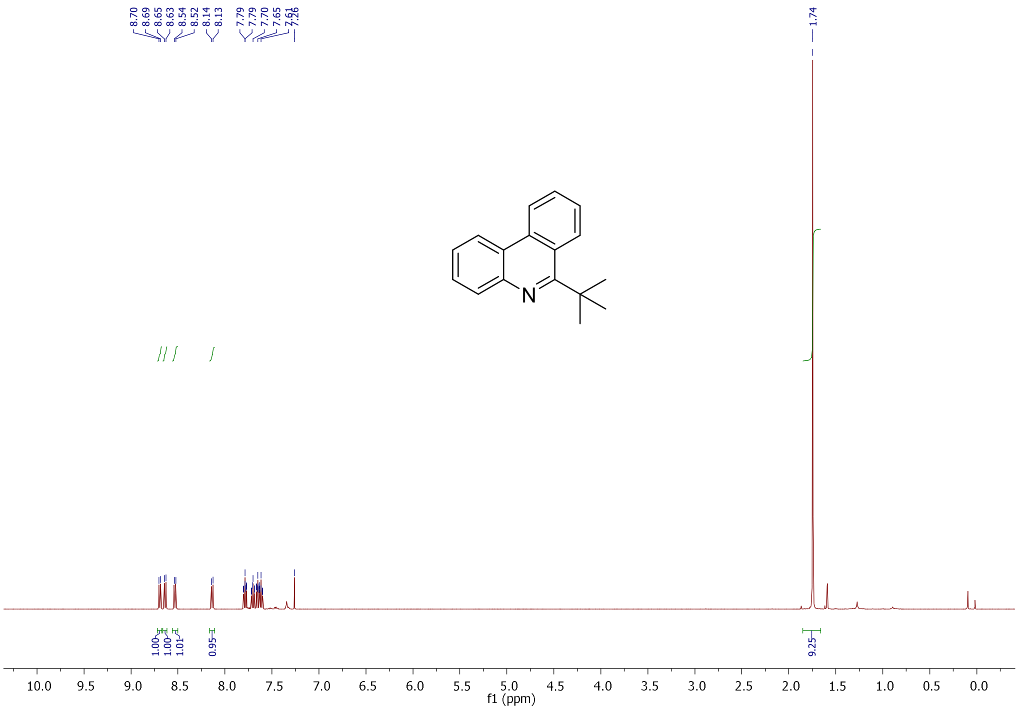
8.70
8.69
8.65
8.63
8.54
8.52
8.14
8.13
7.79
7.79
7.70
7.65
7.61
7.26
1.74
N
1.00
1.00
1.01
0.95
9.25
10.0 9.5 9.0 8.5 8.0 7.5 7.0 6.5 6.0 5.5 5.0 4.5 4.0 3.5 3.0 2.5 2.0 1.5 1.0 0.5 0.0
f1 (ppm)

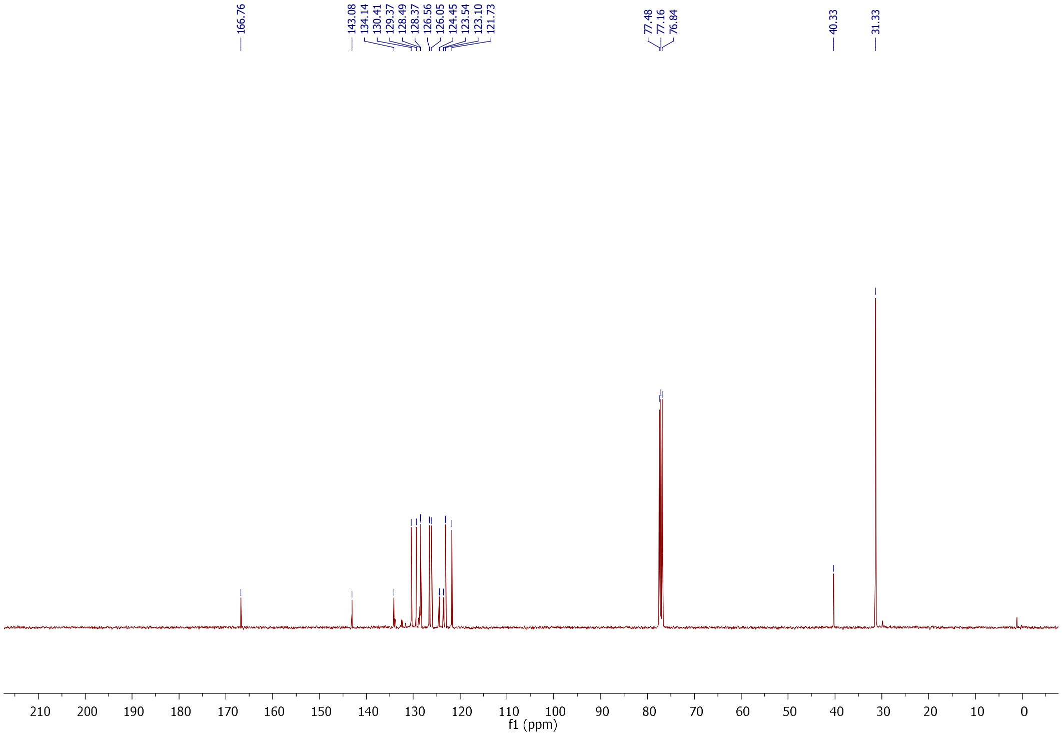
166.76
143.08
134.14
130.41
129.37
128.49
128.37
126.56
126.05
124.45
123.54
123.10
121.73
77.48
77.16
76.84
40.33
31.33
210 200 190 180 170 160 150 140 130 120 110 100 90 80 70 60 50 40 30 20 10 0
f1 (ppm)

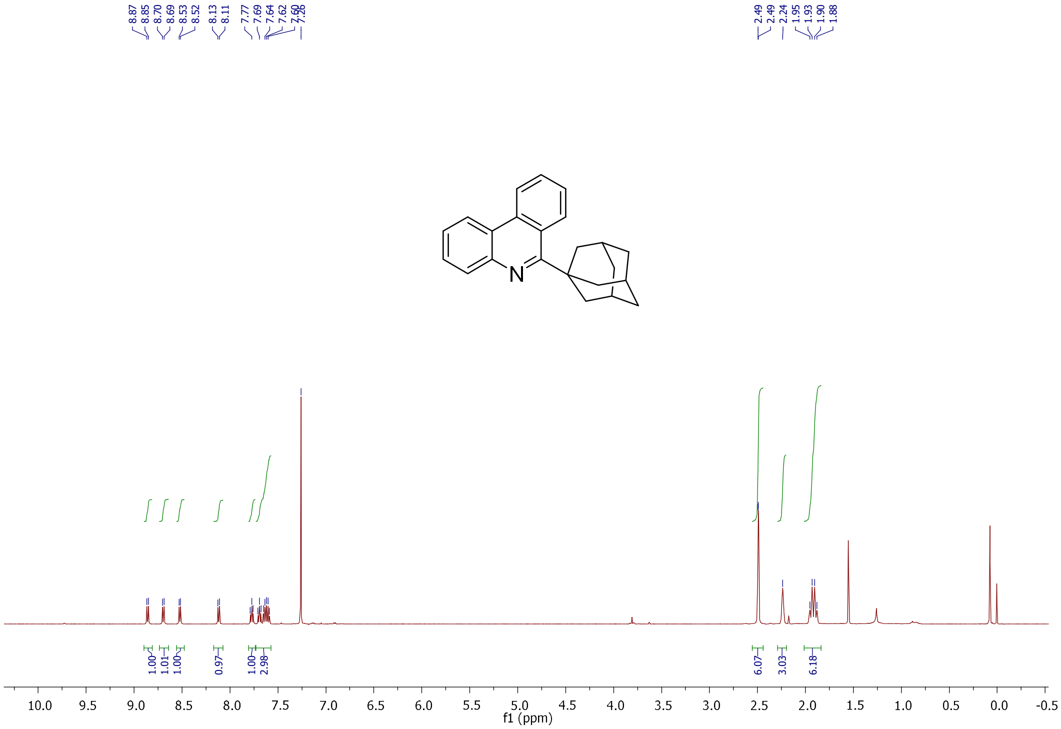
8.87
8.85
8.70
8.69
8.53
8.52
8.13
8.11
7.77
7.69
7.64
7.62
7.60
7.58
2.49
2.49
2.24
1.95
1.93
1.90
1.88
N
1.00
1.01
1.00
0.97
1.00
2.98
6.07
3.03
6.18
f1 (ppm)

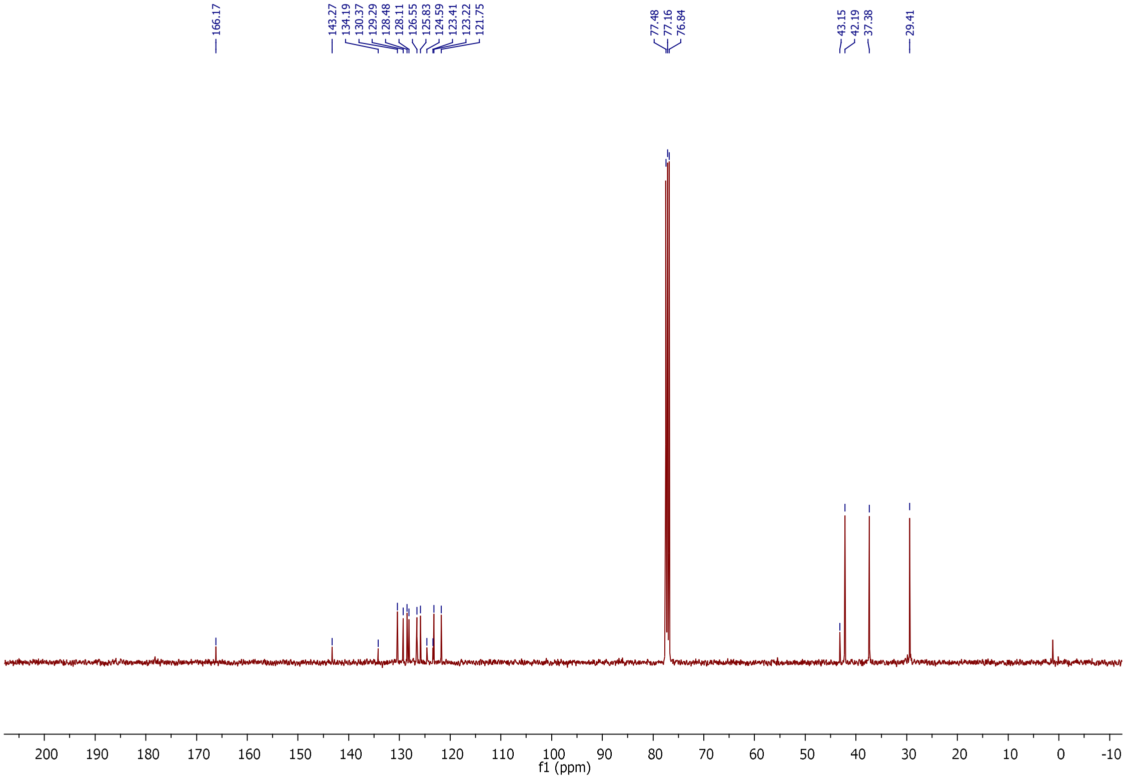
166.17
143.27
134.19
130.37
129.29
128.48
128.11
126.55
125.83
124.59
123.41
123.22
121.75
77.48
77.16
76.84
43.15
42.19
37.38
29.41
f1 (ppm)

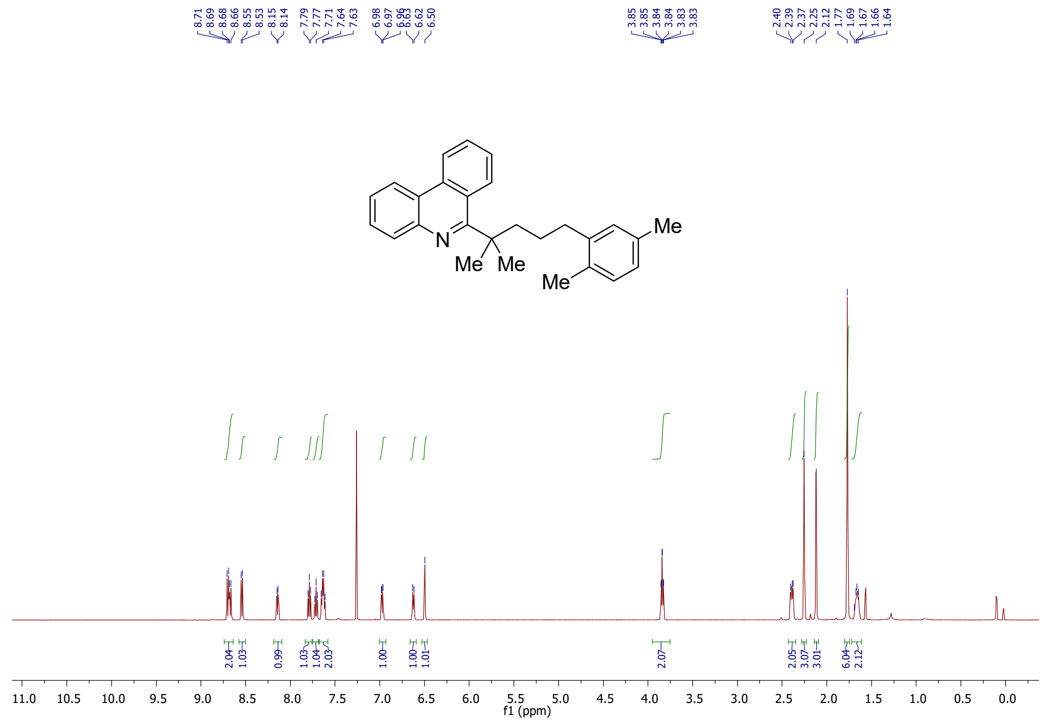

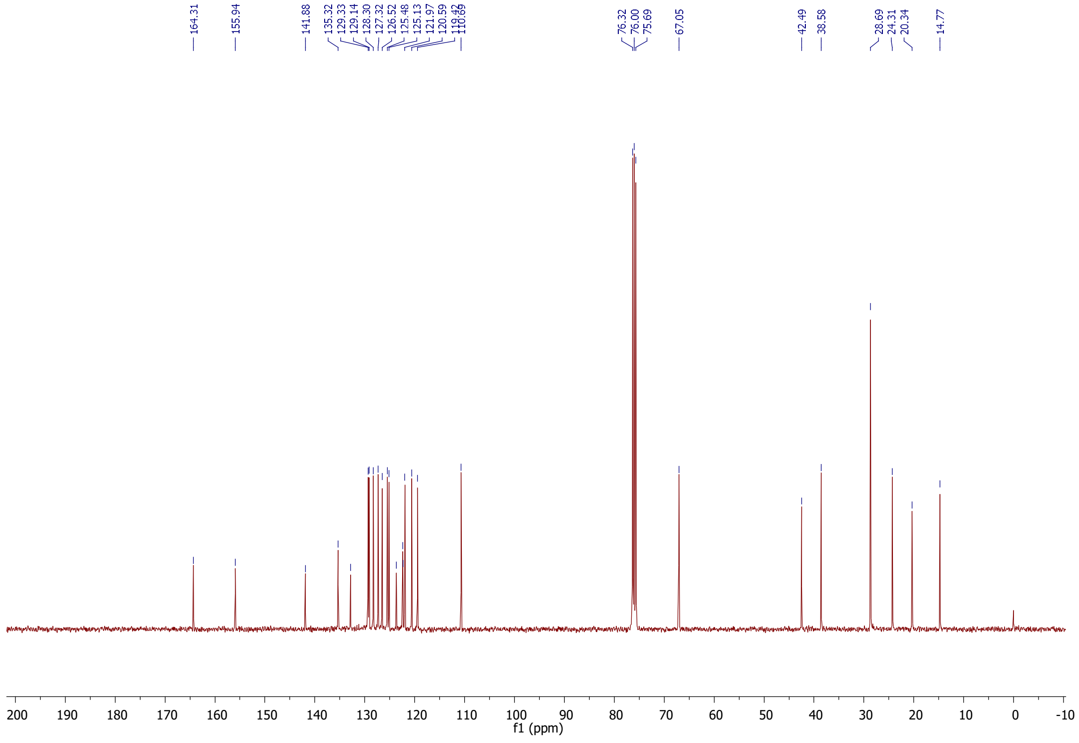

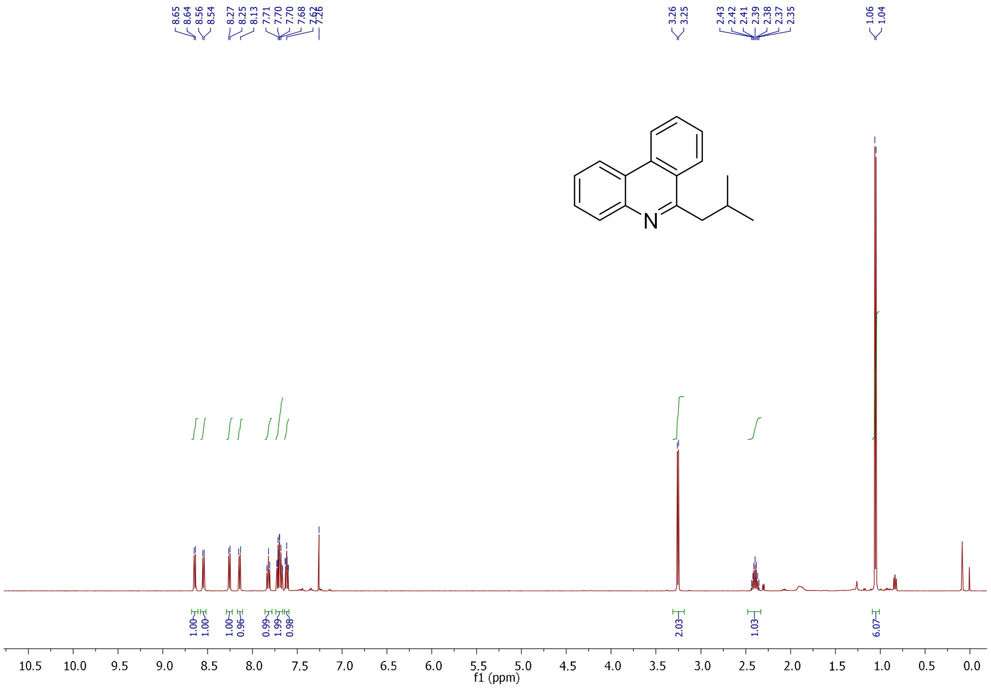
8.65
8.64
8.56
8.54
8.27
8.25
8.13
7.71
7.70
7.70
7.68
7.62
7.26
3.26
3.25
2.43
2.42
2.41
2.39
2.38
2.37
2.35
1.06
1.04
N
1.00
1.00
1.00
0.96
0.99
1.99
0.98
2.03
1.03
6.07
10.5 10.0 9.5 9.0 8.5 8.0 7.5 7.0 6.5 6.0 5.5 5.0 4.5 4.0 3.5 3.0 2.5 2.0 1.5 1.0 0.5 0.0
f1 (ppm)

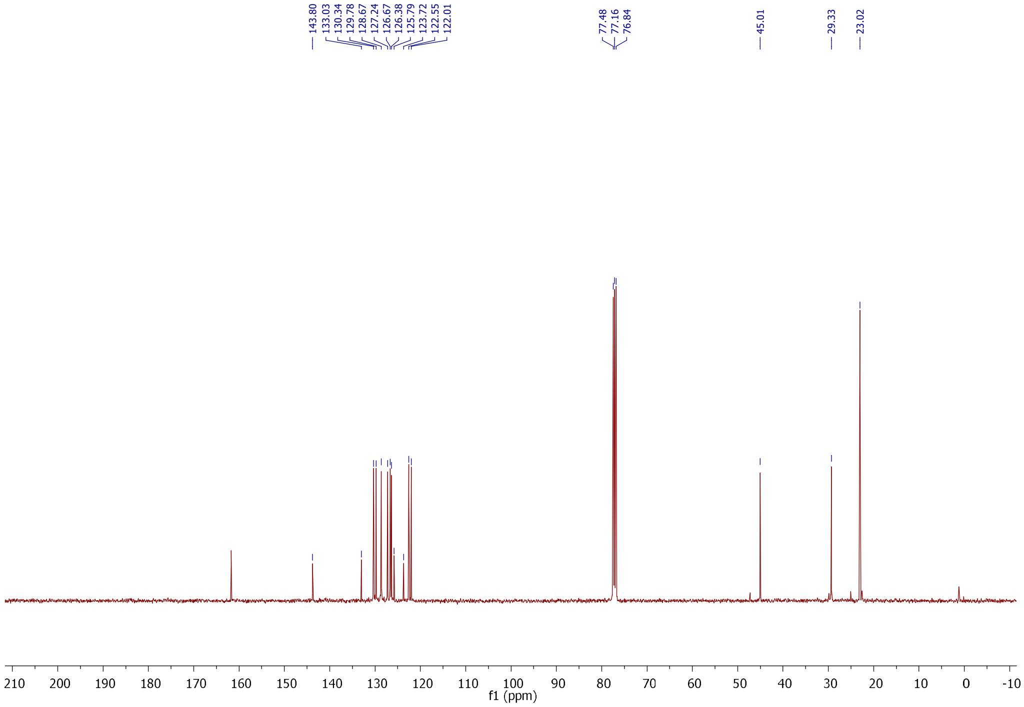
143.80
133.03
130.34
129.78
128.67
127.24
126.67
126.38
125.79
123.72
122.55
122.01
77.48
77.16
76.84
45.01
29.33
23.02
210 200 190 180 170 160 150 140 130 120 110 100 90 80 70 60 50 40 30 20 10 0 -10
f1 (ppm)

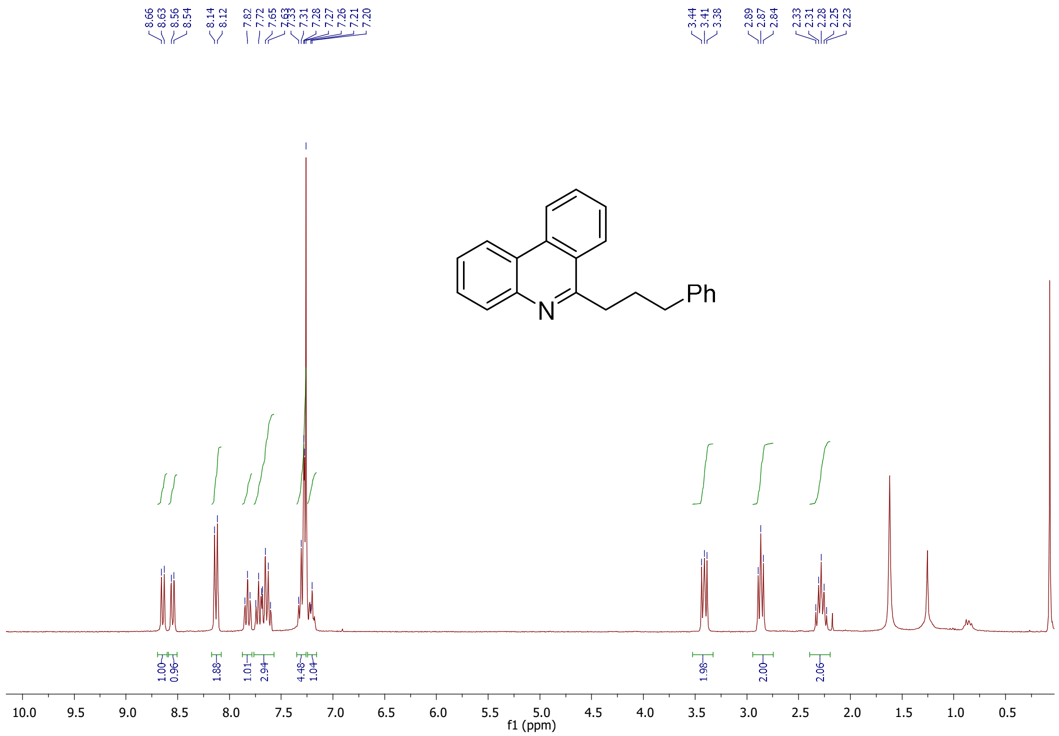
8.66
8.63
8.56
8.54
8.14
8.12
7.82
7.72
7.65
7.63
7.33
7.31
7.28
7.27
7.26
7.21
7.20
3.44
3.41
3.38
2.89
2.87
2.84
2.33
2.31
2.28
2.25
2.23
N
Ph
1.00
0.96
1.88
1.01
2.94
4.48
1.04
1.98
2.00
2.06
f1 (ppm)

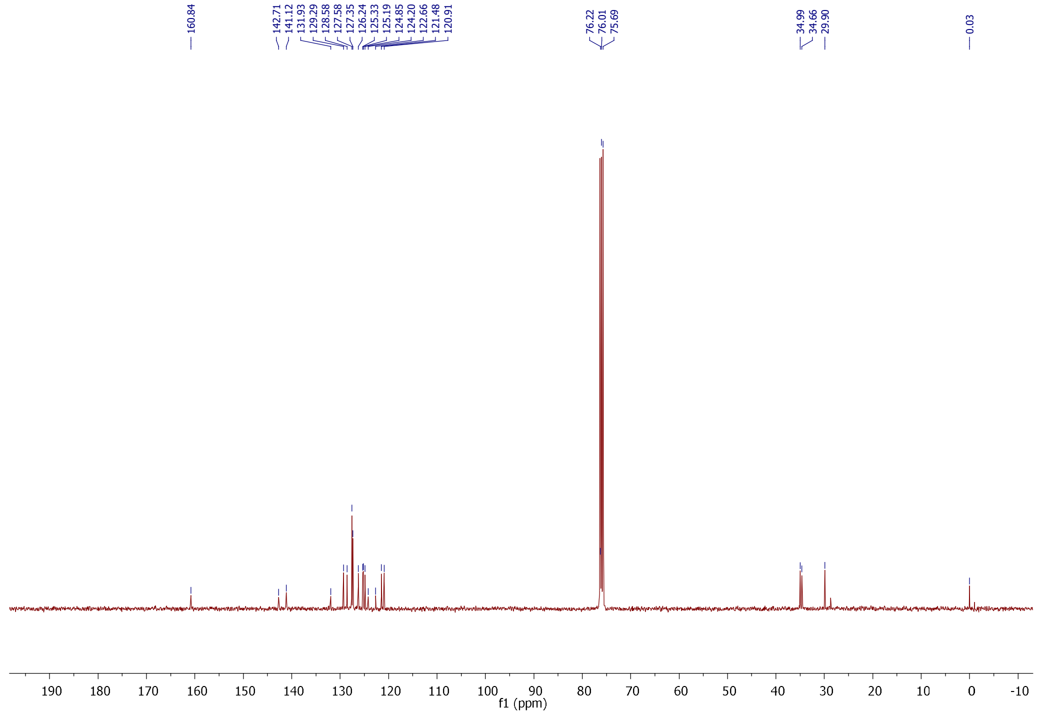
160.84
142.71
141.12
131.93
129.29
128.58
127.58
127.35
126.24
125.33
125.19
124.85
124.20
122.66
121.48
120.91
76.22
76.01
75.69
34.99
34.66
29.90
0.03
f1 (ppm)

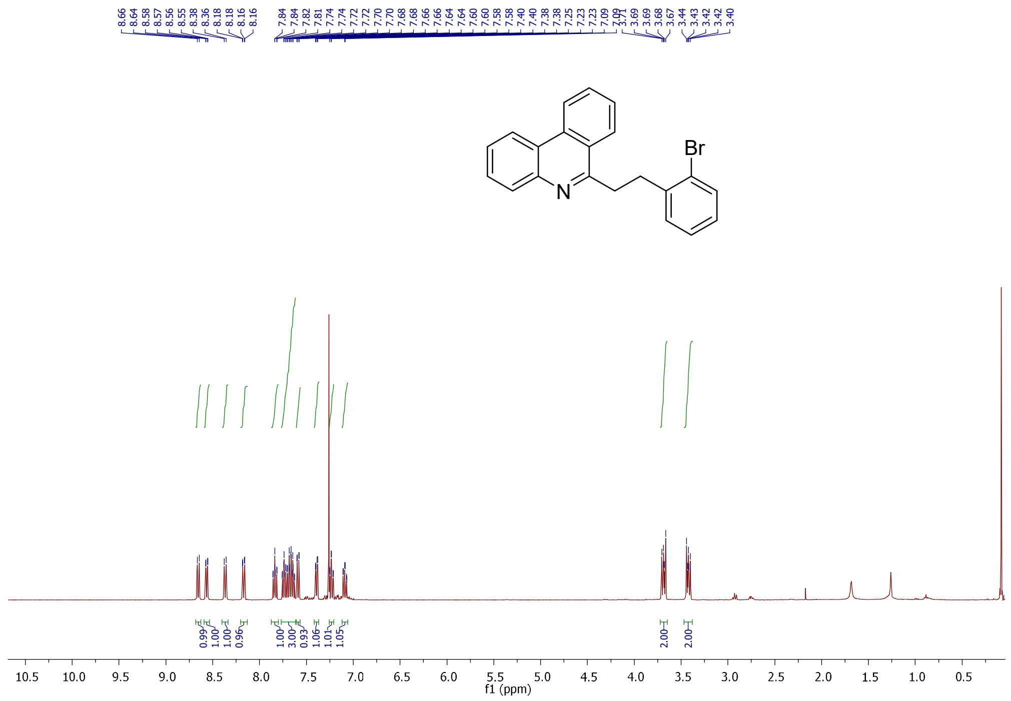
8.66 8.64 8.58 8.57 8.56 8.55 8.38 8.36 8.18 8.18 8.16 8.16 7.84 7.84 7.82 7.81 7.74 7.74 7.72 7.72 7.70 7.70 7.68 7.68 7.66 7.66 7.64 7.60 7.60 7.58 7.58 7.40 7.40 7.38 7.38 7.25 7.23 7.23 7.09 7.09 3.71 3.69 3.69 3.68 3.67 3.44 3.43 3.42 3.42 3.40
Br
N
0.99 1.00 1.00 0.96 1.00 3.00 0.93 1.06 1.01 1.05 2.00 2.00
10.5 10.0 9.5 9.0 8.5 8.0 7.5 7.0 6.5 6.0 5.5 5.0 4.5 4.0 3.5 3.0 2.5 2.0 1.5 1.0 0.5
f1 (ppm)

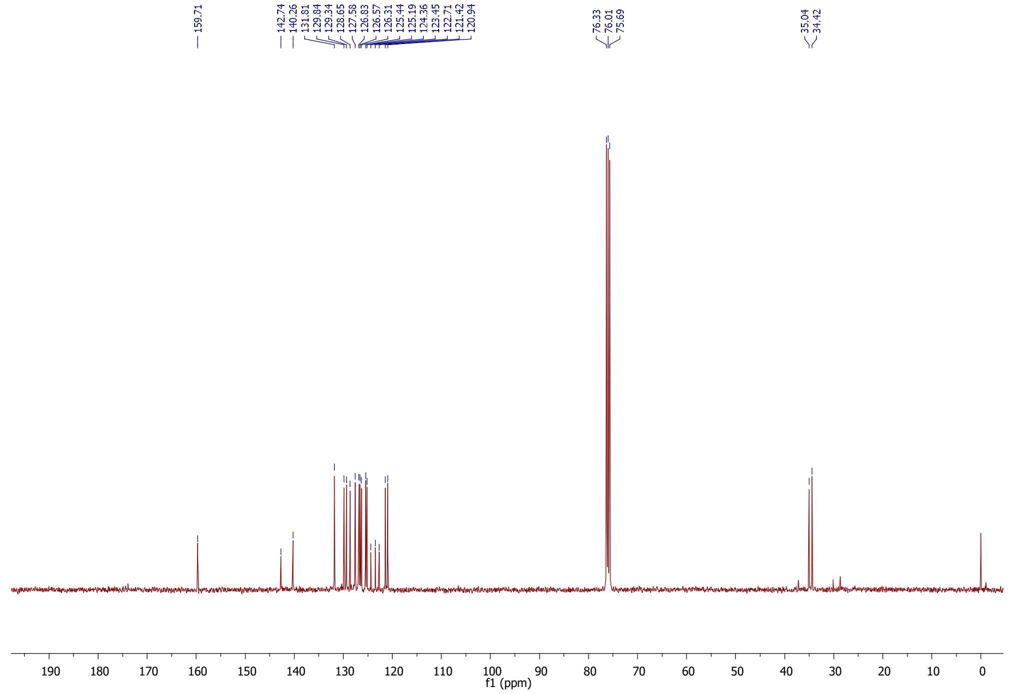
159.71
142.74 140.26 131.81 129.84 129.34 128.65 127.58 126.83 126.57 126.31 125.44 125.19 124.36 123.45 122.71 121.42 120.94
76.33 76.01 75.69
35.04 34.42
190 180 170 160 150 140 130 120 110 100 90 80 70 60 50 40 30 20 10 0
f1 (ppm)

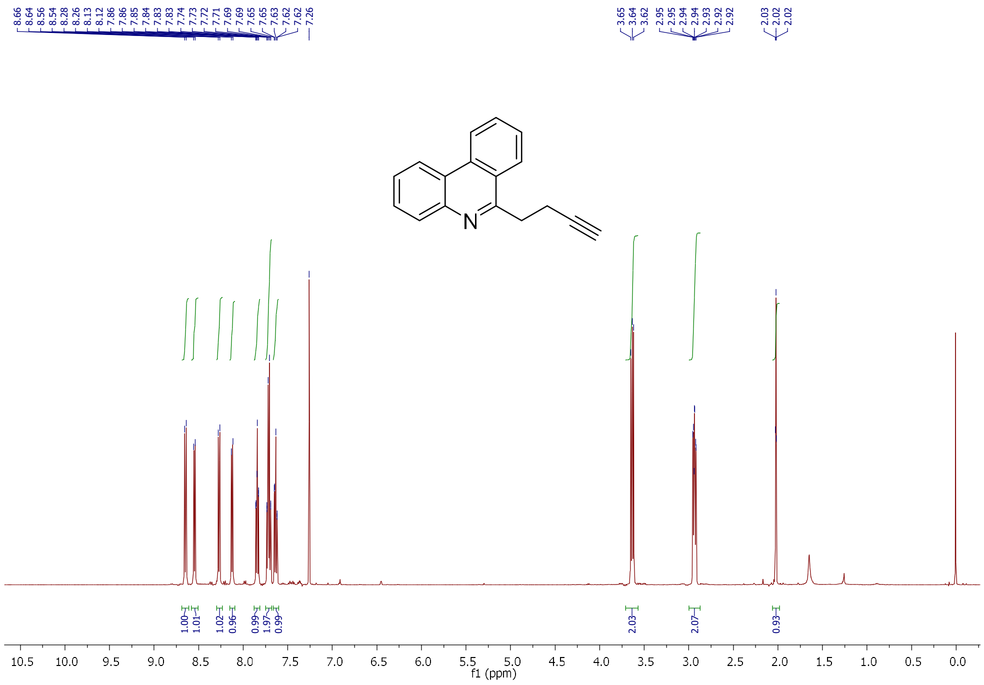
8.66 8.64 8.56 8.54 8.28 8.26 8.13 8.12 7.86 7.86 7.85 7.84 7.83 7.83 7.74 7.73 7.72 7.71 7.69 7.69 7.65 7.63 7.62 7.62 7.26
3.65 3.64 3.62
2.95 2.95 2.94 2.94 2.93 2.92 2.92
2.03 2.02 2.02
N
1.00 1.01 1.02 0.96 0.99 1.97 0.99
2.03
2.07
0.93
10.5 10.0 9.5 9.0 8.5 8.0 7.5 7.0 6.5 6.0 5.5 5.0 4.5 4.0 3.5 3.0 2.5 2.0 1.5 1.0 0.5 0.0
f1 (ppm)

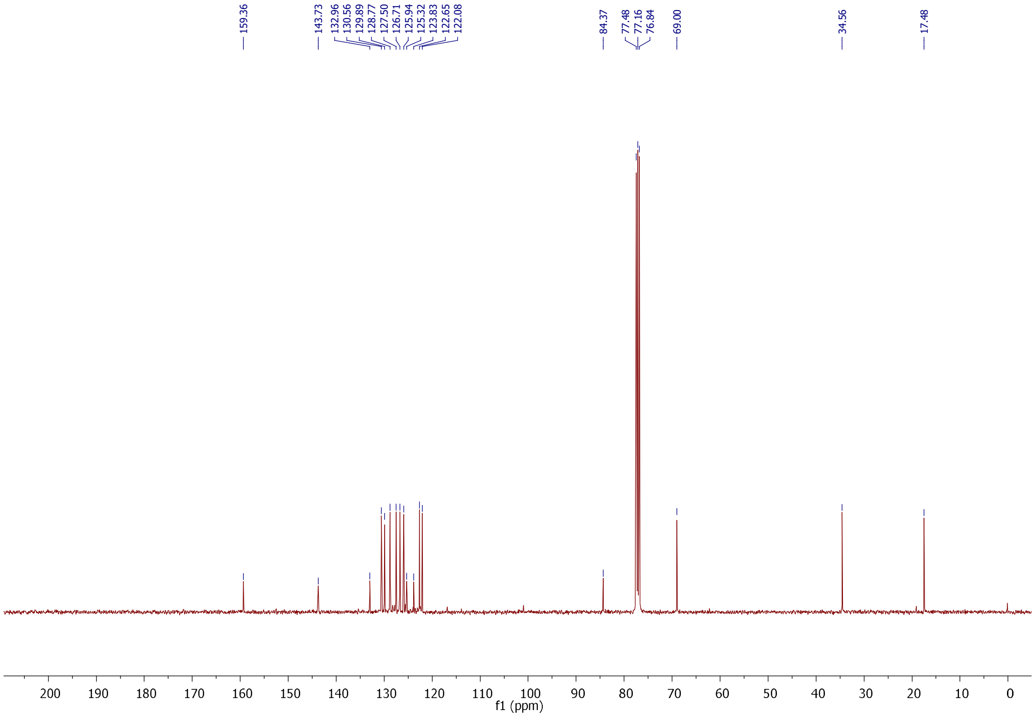
159.36
143.73
132.96 130.56 129.89 128.77 127.50 126.71 125.94 125.32 123.83 122.65 122.08
84.37
77.48 77.16 76.84
69.00
34.56
17.48
200 190 180 170 160 150 140 130 120 110 100 90 80 70 60 50 40 30 20 10 0
f1 (ppm)

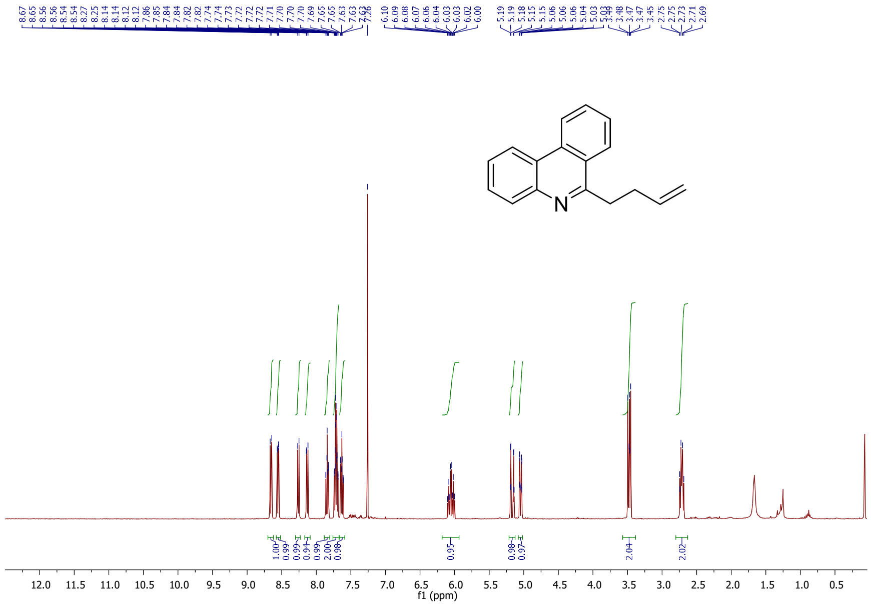
N
f1 (ppm)

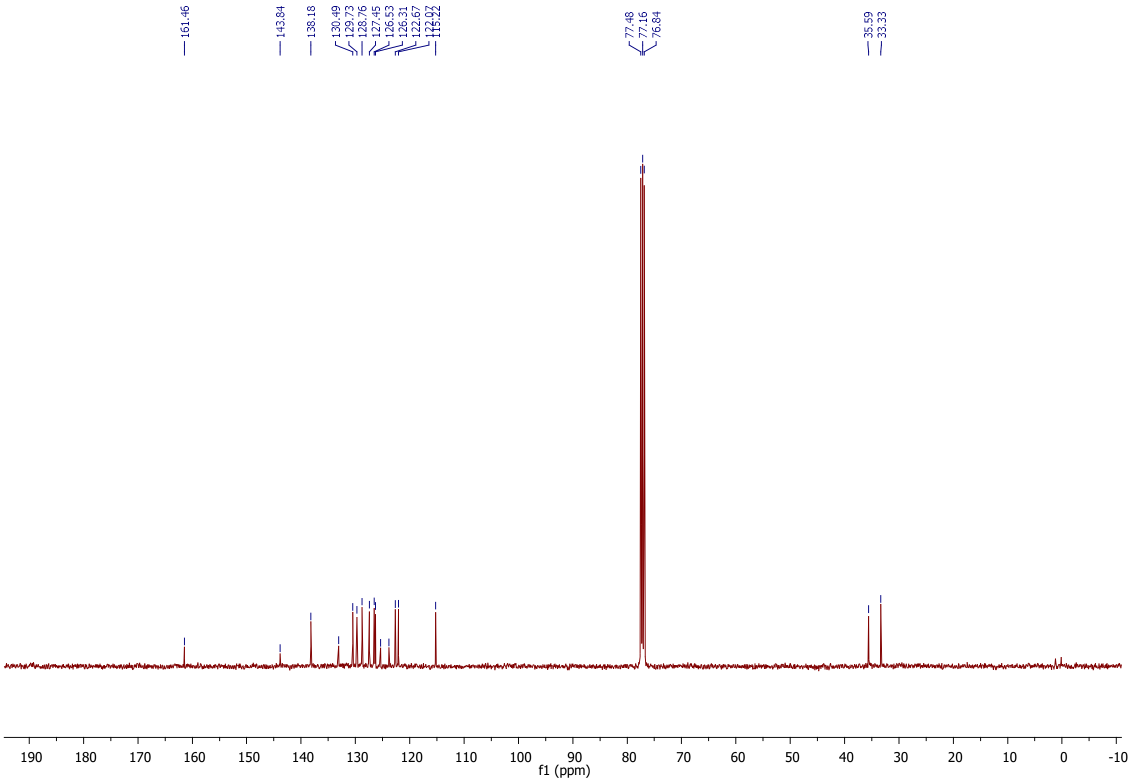
161.46
143.84
138.18
130.49
129.73
128.76
127.45
126.53
126.31
122.97
122.67
115.22
77.48
77.16
76.84
35.59
33.33
f1 (ppm)

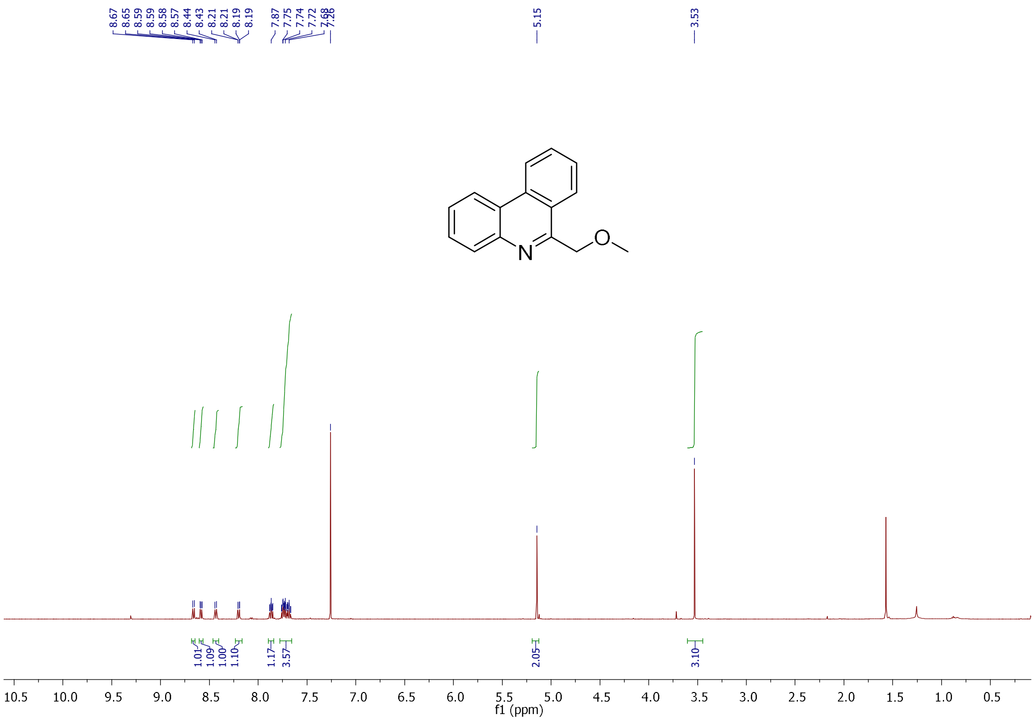
8.67 8.65 8.59 8.59 8.58 8.57 8.44 8.43 8.21 8.19 8.19 7.87 7.75 7.74 7.72 7.68 7.26
5.15
3.53
N
O
1.01 1.09 1.00 1.10 1.17 3.57
2.05
3.10
10.5 10.0 9.5 9.0 8.5 8.0 7.5 7.0 6.5 6.0 5.5 5.0 4.5 4.0 3.5 3.0 2.5 2.0 1.5 1.0 0.5
f1 (ppm)

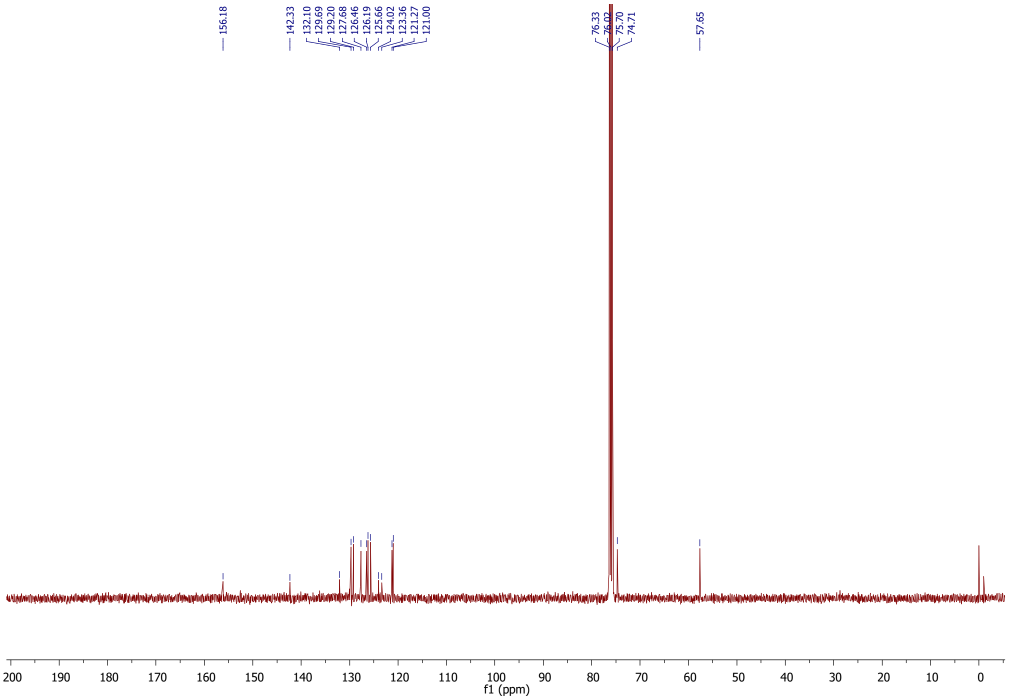
156.18
142.33
132.10 129.69 129.20 127.68 126.46 126.19 125.66 124.02 123.36 121.27 121.00
76.33 76.02 75.70 74.71
57.65
200 190 180 170 160 150 140 130 120 110 100 90 80 70 60 50 40 30 20 10 0
f1 (ppm)

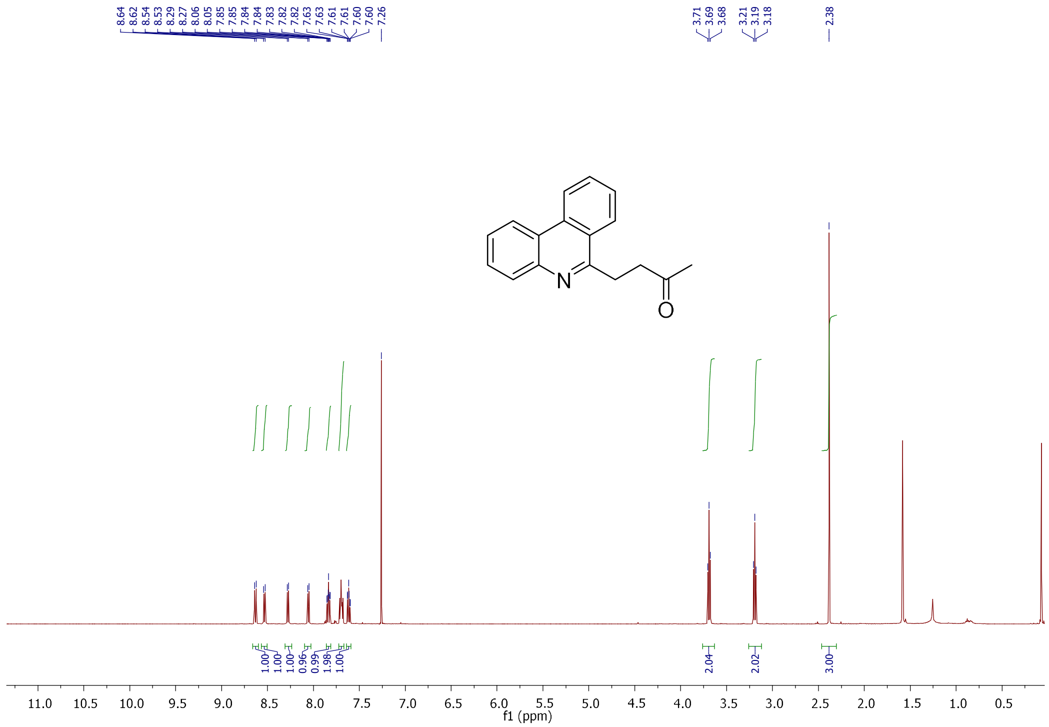

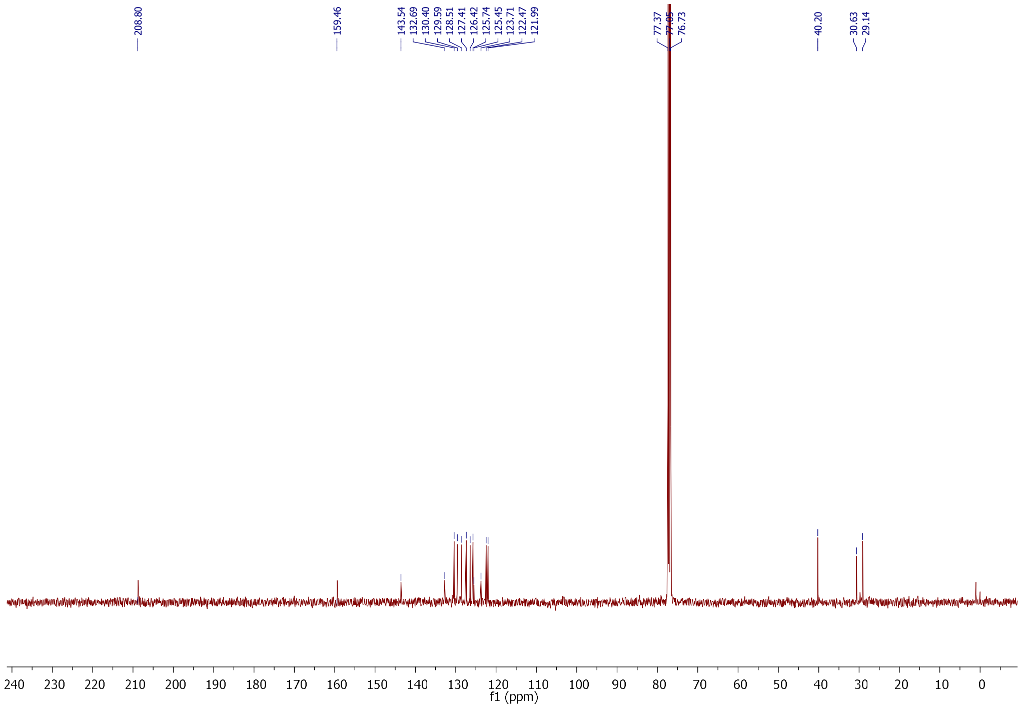

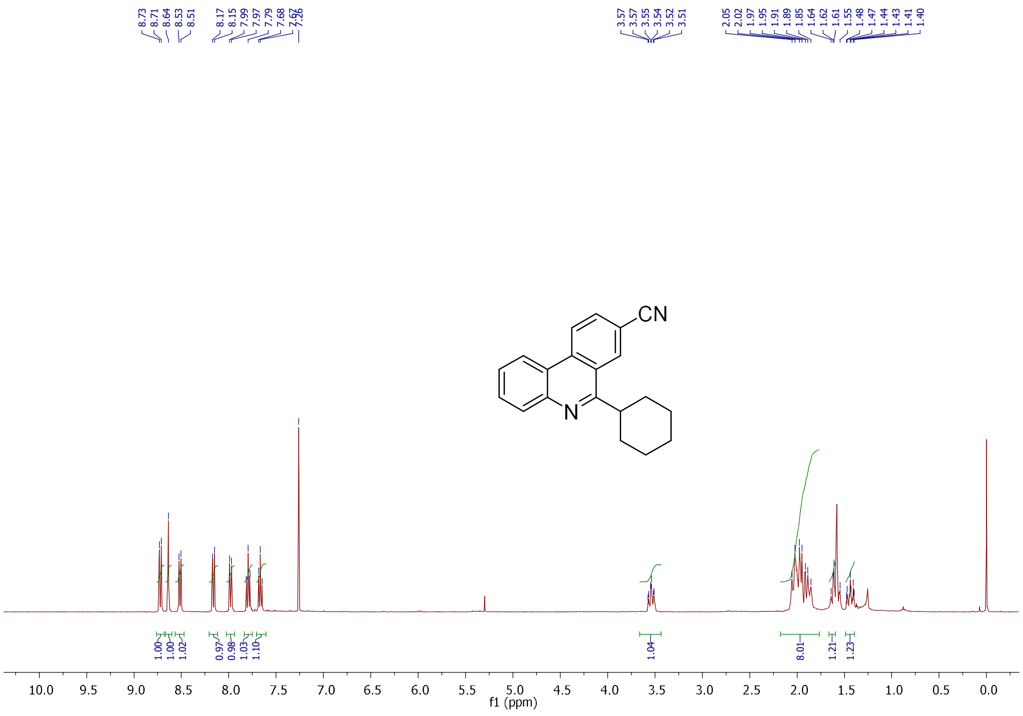

8.73
8.71
8.64
8.53
8.51
8.17
8.15
7.99
7.97
7.79
7.68
7.67
7.26
3.57
3.57
3.55
3.54
3.52
3.51
2.05
2.02
1.97
1.95
1.91
1.89
1.85
1.64
1.62
1.61
1.55
1.48
1.47
1.44
1.43
1.41
1.40
CN
N
1.00
1.00
1.02
0.97
0.98
1.03
1.10
1.04
8.01
1.21
1.23
f1 (ppm)

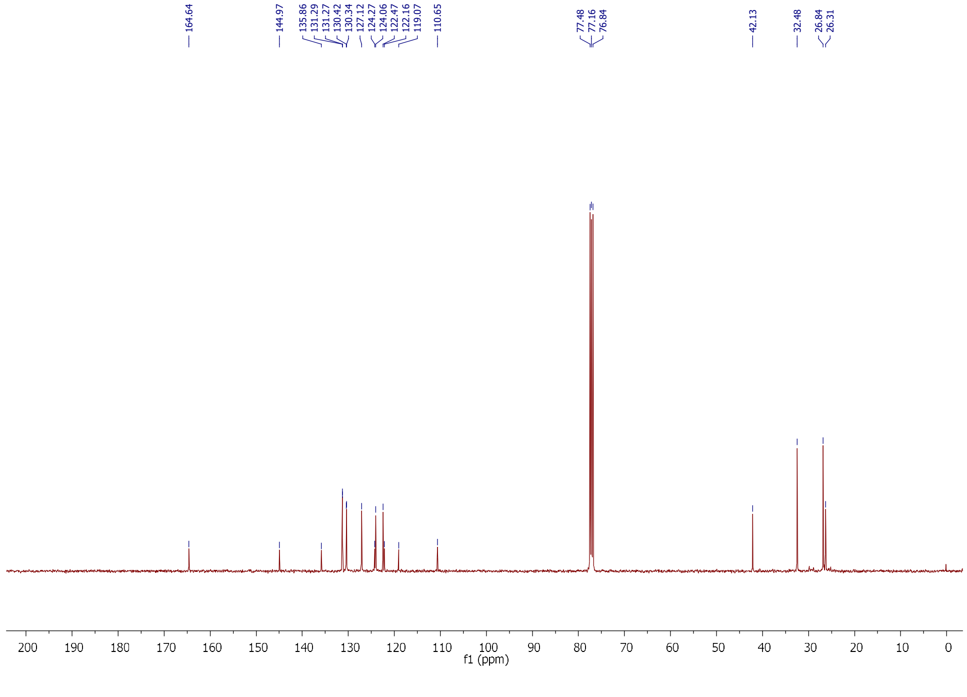

164.64
144.97
135.86
131.29
131.27
130.42
130.34
127.12
124.27
124.06
122.47
122.16
119.07
110.65
77.48
77.16
76.84
42.13
32.48
26.84
26.31
f1 (ppm)

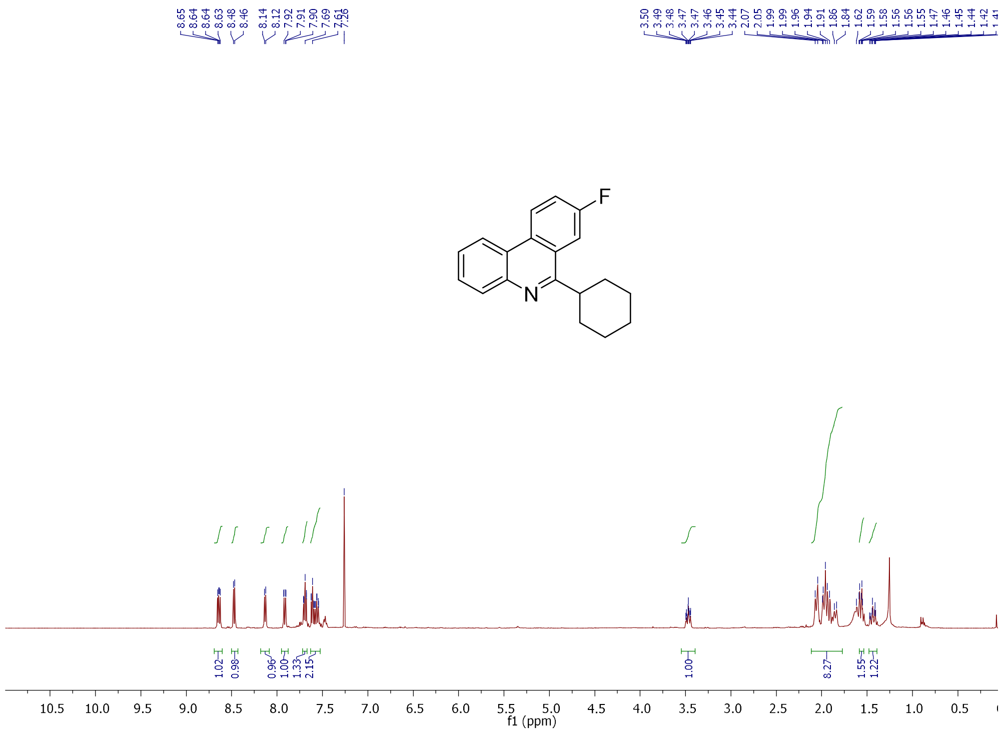

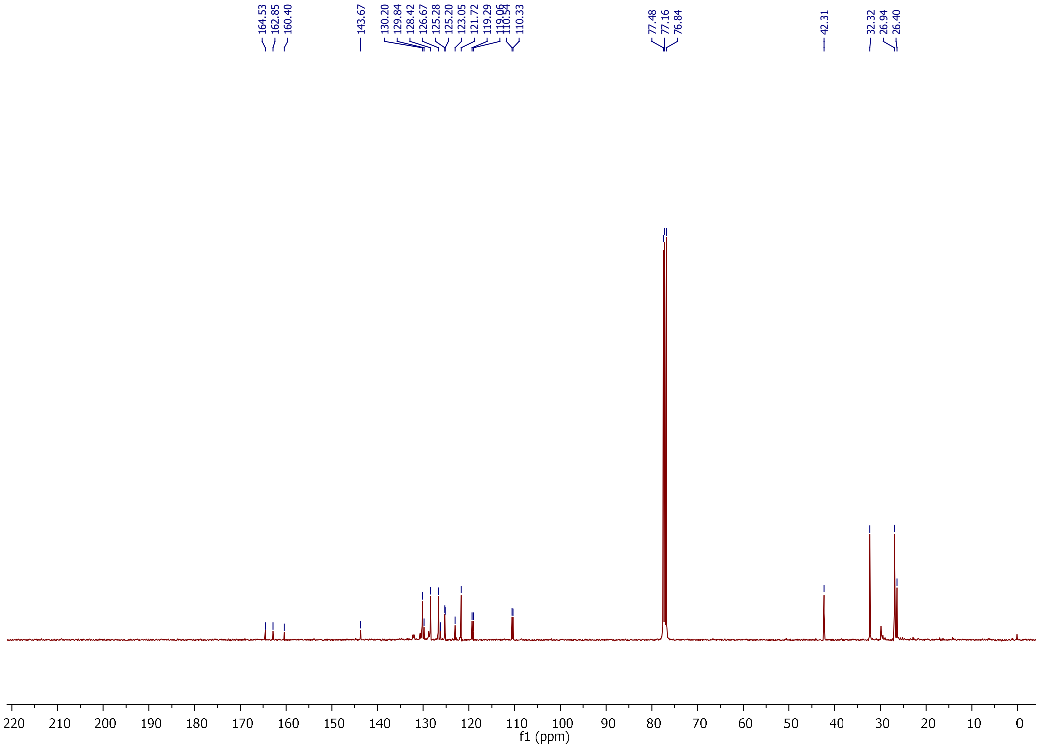

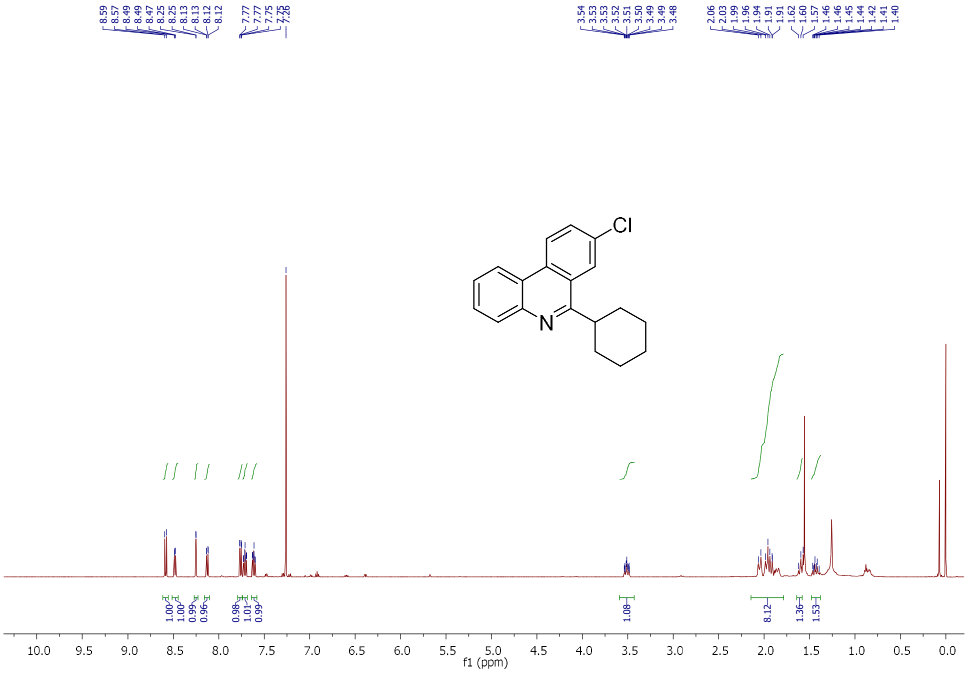
8.59 8.57 8.49 8.47 8.25 8.25 8.13 8.13 8.12 8.12 7.77 7.77 7.75 7.26
3.54 3.53 3.53 3.52 3.51 3.50 3.49 3.49 3.48
2.06 2.03 1.99 1.96 1.94 1.91 1.91 1.62 1.60 1.57 1.46 1.46 1.45 1.44 1.42 1.41 1.40
Cl
N
1.00 1.00 0.99 0.96 0.98 1.01 0.99
1.08
8.12
1.36 1.53
10.0 9.5 9.0 8.5 8.0 7.5 7.0 6.5 6.0 5.5 5.0 4.5 4.0 3.5 3.0 2.5 2.0 1.5 1.0 0.5 0.0
f1 (ppm)

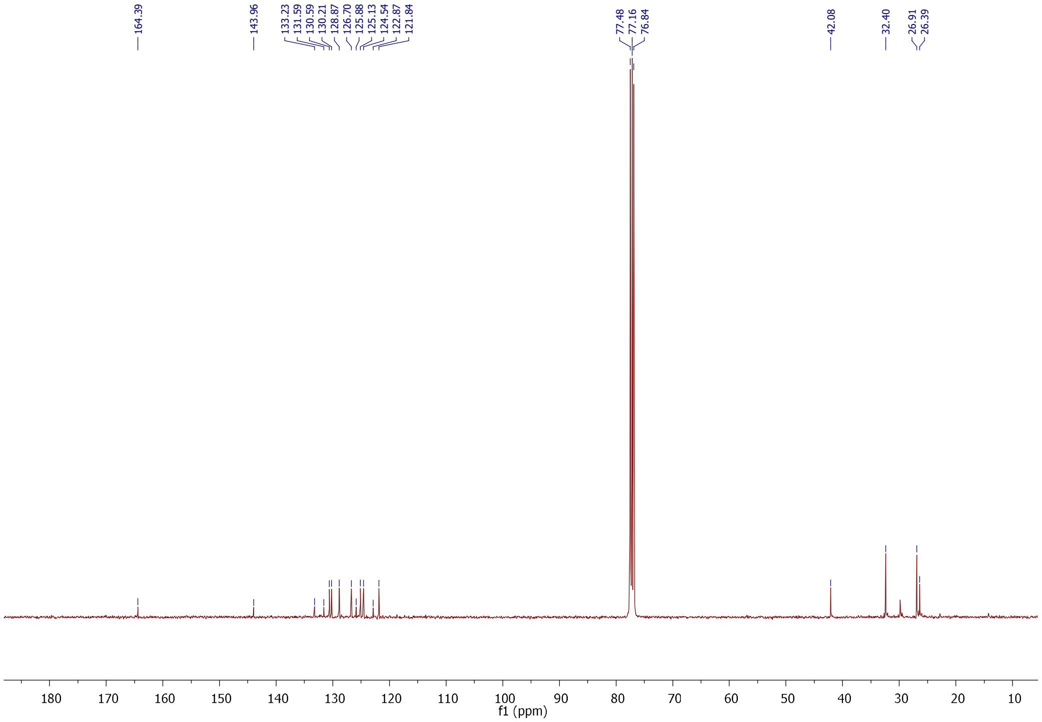
164.39
143.96
133.23 131.59 130.59 130.21 128.87 126.70 125.88 125.13 124.54 122.87 121.84
77.48 77.16 76.84
42.08
32.40
26.91 26.39
180 170 160 150 140 130 120 110 100 90 80 70 60 50 40 30 20 10
f1 (ppm)

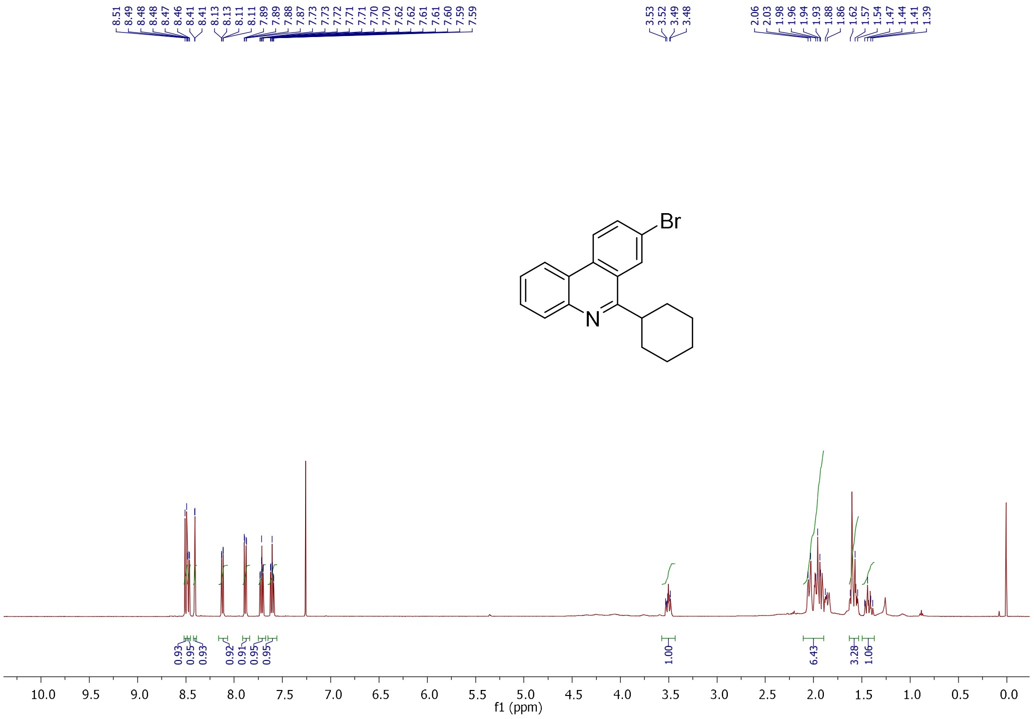
8.51 8.49 8.48 8.48 8.47 8.46 8.41 8.41 8.13 8.13 8.11 8.11 7.89 7.89 7.88 7.87 7.73 7.73 7.72 7.71 7.71 7.70 7.70 7.62 7.62 7.61 7.60 7.59 7.59
3.53 3.52 3.49 3.48
2.06 2.03 1.98 1.96 1.94 1.93 1.88 1.86 1.62 1.57 1.54 1.47 1.44 1.41 1.39
Br
N
0.93 0.95 0.93 0.92 0.91 0.95 0.95
1.00
6.43
3.28 1.06
10.0 9.5 9.0 8.5 8.0 7.5 7.0 6.5 6.0 5.5 5.0 4.5 4.0 3.5 3.0 2.5 2.0 1.5 1.0 0.5 0.0
f1 (ppm)

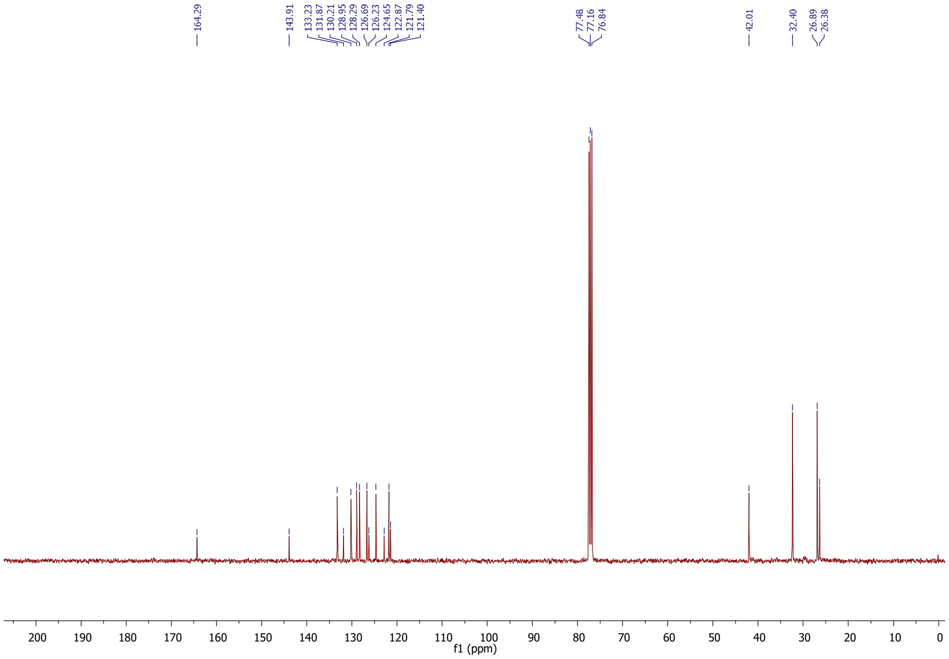
164.29
143.91
133.23 131.87 130.21 128.95 128.29 126.69 126.23 124.65 122.87 121.79 121.40
77.48 77.16 76.84
42.01
32.40
26.89 26.38
200 190 180 170 160 150 140 130 120 110 100 90 80 70 60 50 40 30 20 10 0
f1 (ppm)

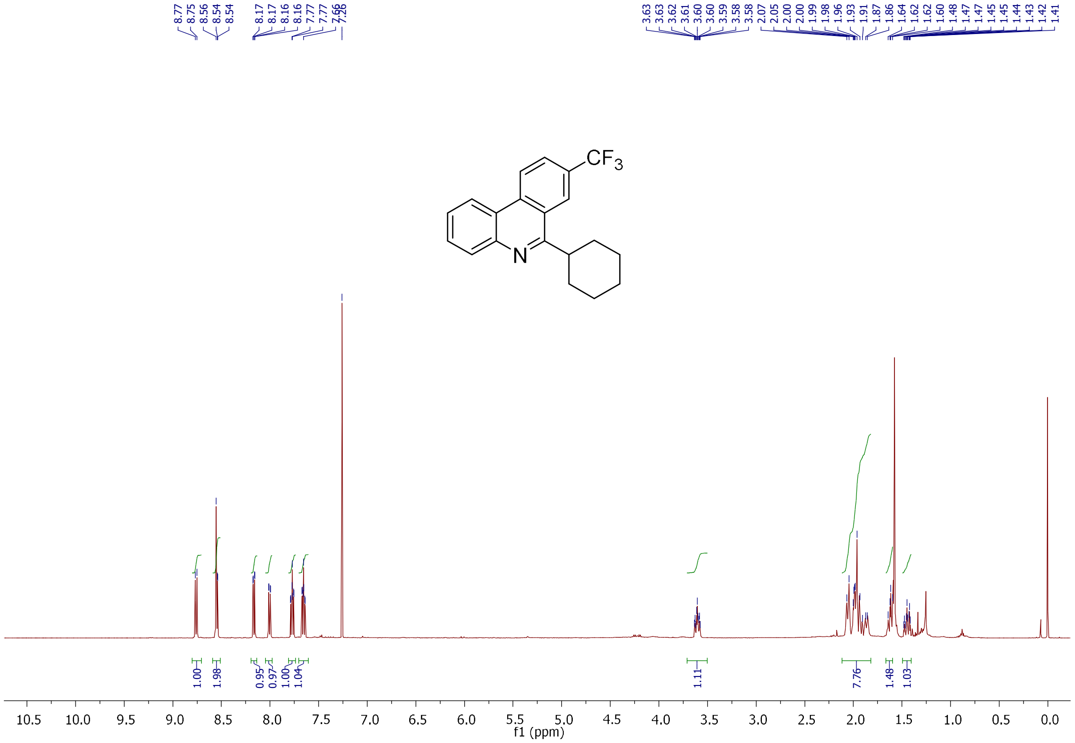
8.77 8.75 8.56 8.54 8.54 8.17 8.17 8.16 8.16 7.77 7.77
3.63 3.63 3.62 3.61 3.60 3.60 3.59 3.58 3.58 2.07 2.05 2.00 2.00 1.99 1.98 1.96 1.93 1.91 1.87 1.86 1.64 1.62 1.62 1.60 1.48 1.47 1.45 1.45 1.44 1.43 1.42 1.41
CF3
N
1.00 1.98 0.95 0.97 1.00 1.04 1.11 7.76 1.48 1.03
10.5 10.0 9.5 9.0 8.5 8.0 7.5 7.0 6.5 6.0 5.5 5.0 4.5 4.0 3.5 3.0 2.5 2.0 1.5 1.0 0.5 0.0
f1 (ppm)

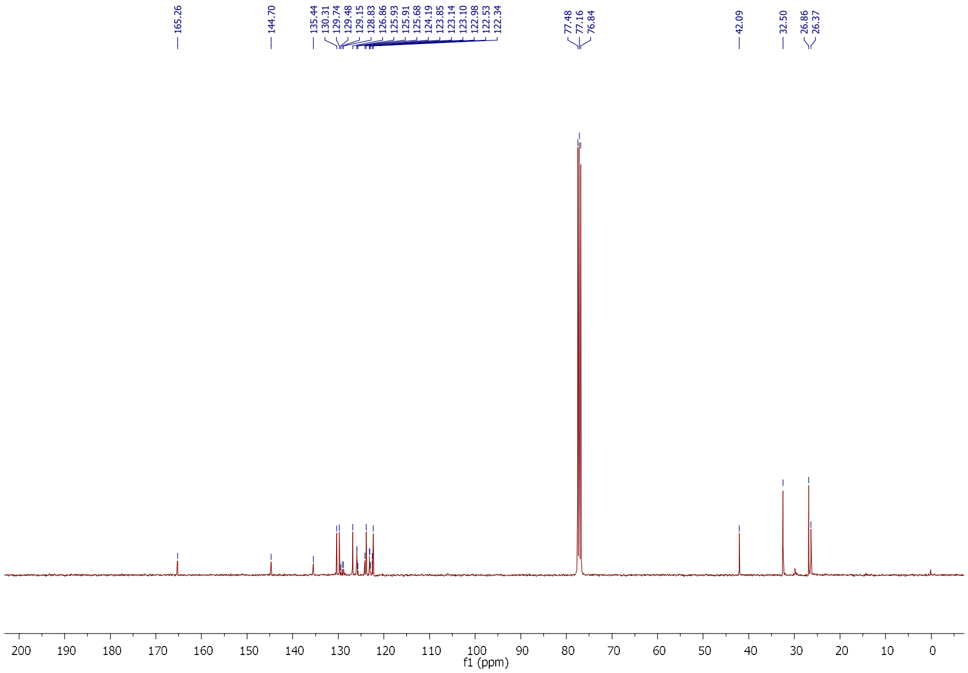
165.26
144.70
135.44 130.31 129.74 129.48 129.15 128.83 126.86 125.93 125.91 125.68 124.19 123.85 123.14 123.10 122.98 122.53 122.34
77.48 77.16 76.84
42.09
32.50 26.86 26.37
200 190 180 170 160 150 140 130 120 110 100 90 80 70 60 50 40 30 20 10 0
f1 (ppm)

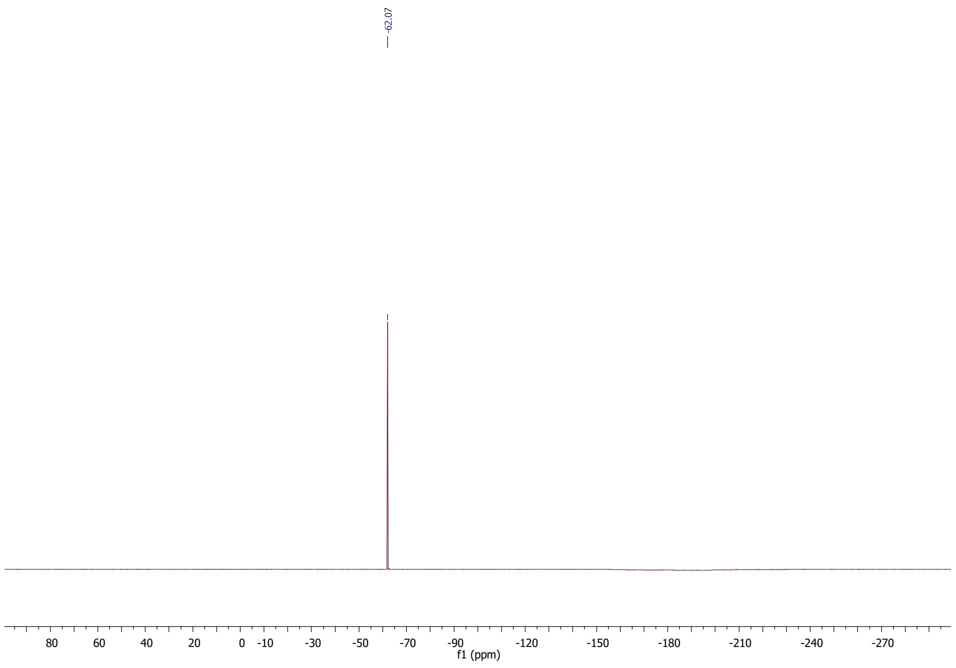
-62.07
80
60
40
20
0
-10
-30
-50
-70
-90
-120
-150
-180
-210
-240
-270
f1 (ppm)

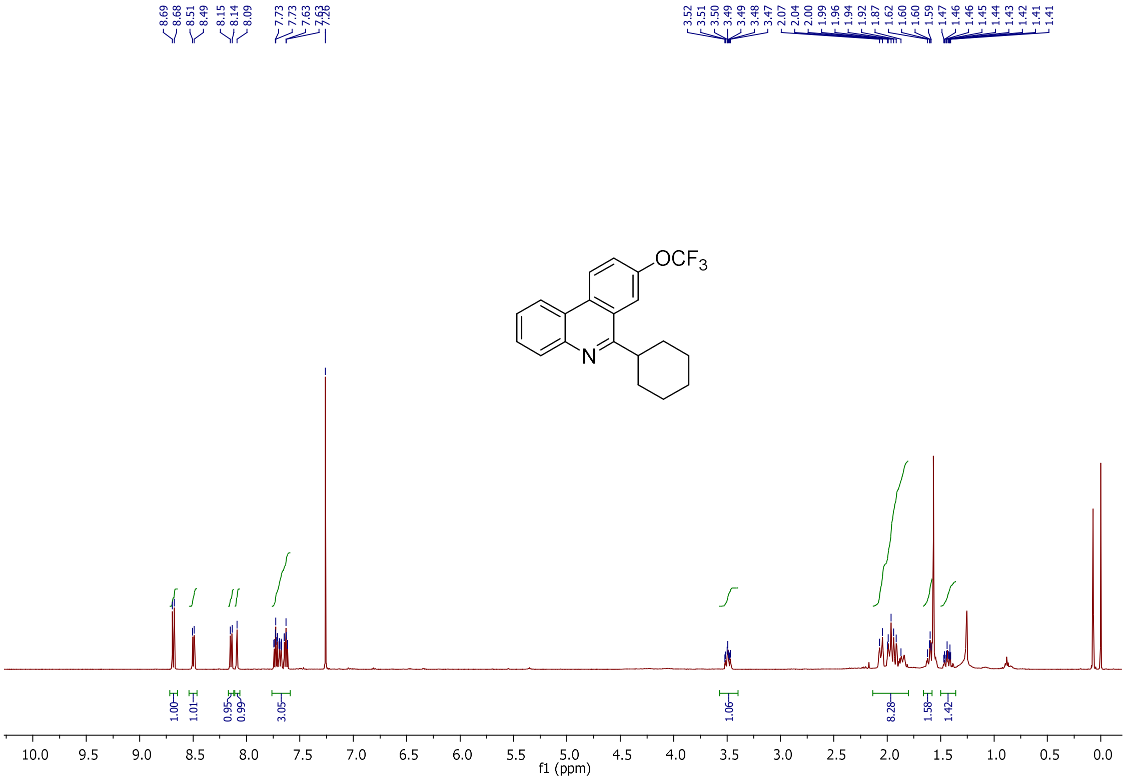
OCF3
N
f1 (ppm)

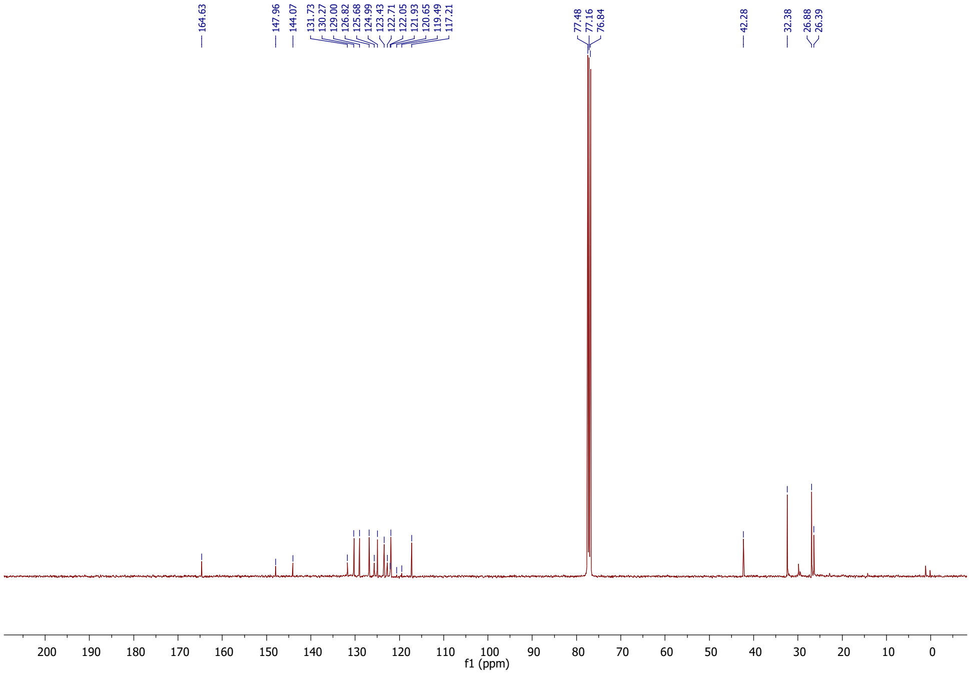
f1 (ppm)

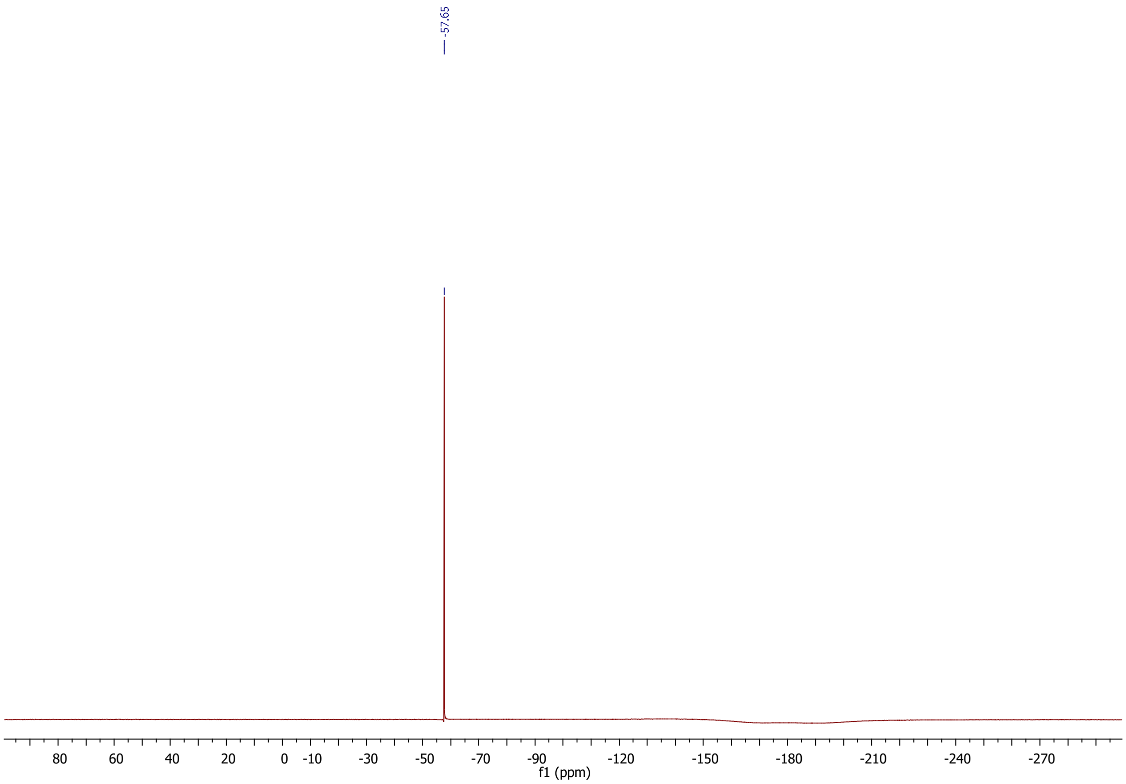
-57.65
80
60
40
20
0
-10
-30
-50
-70
-90
-120
-150
-180
-210
-240
-270
f1 (ppm)

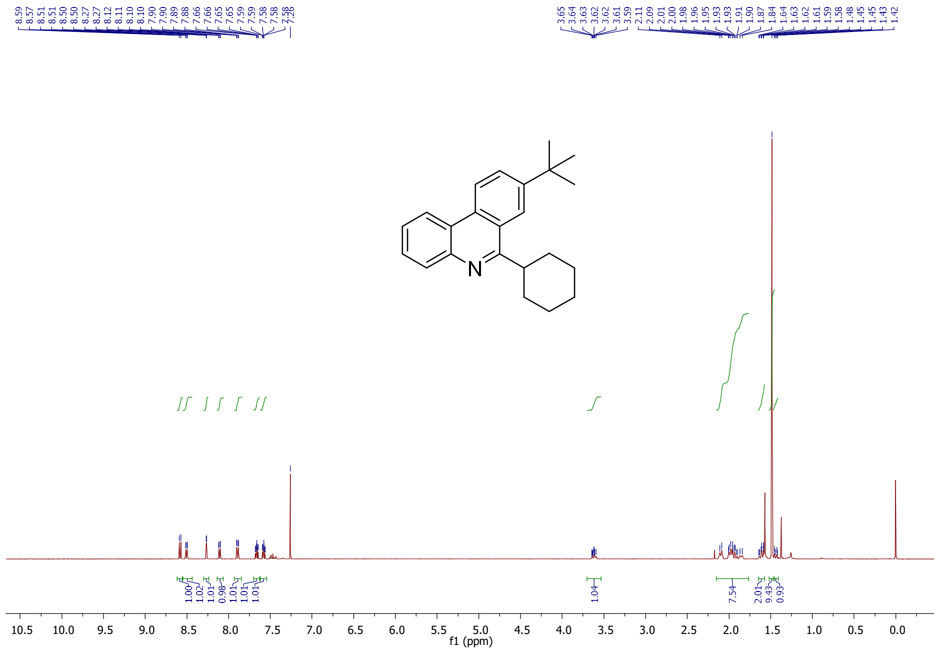
8.59 8.57 8.51 8.50 8.50 8.27 8.12 8.11 8.10 8.10 7.90 7.89 7.88 7.66 7.66 7.65 7.65 7.59 7.59 7.58 7.58 7.28
3.65 3.64 3.63 3.62 3.62 3.61 3.59 2.11 2.09 2.01 2.00 1.98 1.96 1.95 1.93 1.93 1.91 1.90 1.87 1.84 1.64 1.63 1.62 1.61 1.59 1.58 1.48 1.45 1.45 1.43 1.42
N
1.00 1.02 1.01 0.98 1.01 1.01 1.01
1.04
7.54 2.01 9.43 0.93
10.5 10.0 9.5 9.0 8.5 8.0 7.5 7.0 6.5 6.0 5.5 5.0 4.5 4.0 3.5 3.0 2.5 2.0 1.5 1.0 0.5 0.0
f1 (ppm)

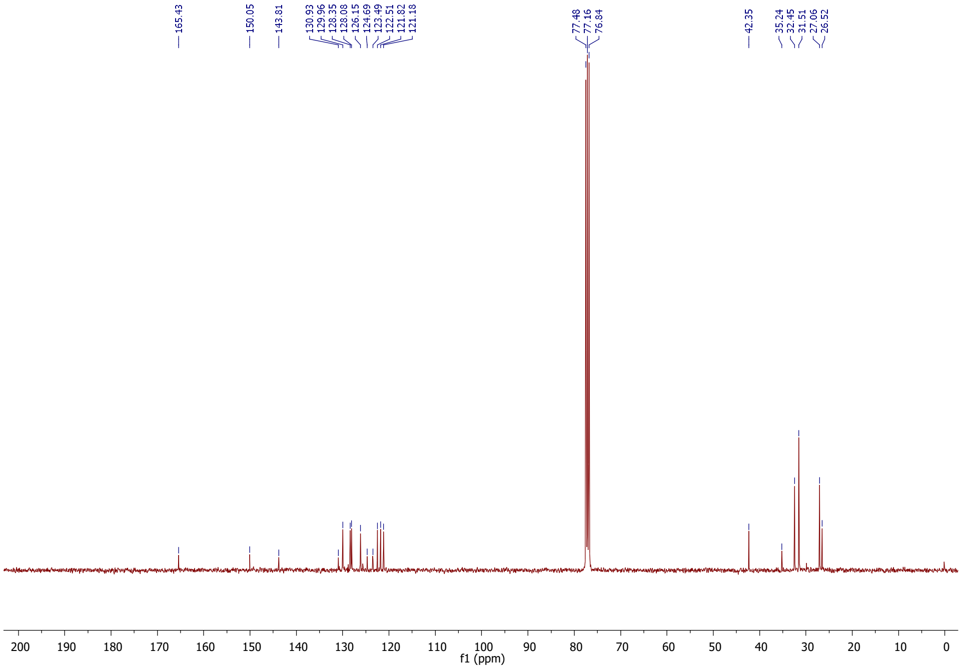
165.43
150.05
143.81
130.93 129.96 128.35 128.08 126.15 124.69 123.49 122.51 121.82 121.18
77.48 77.16 76.84
42.35
35.24 32.45 31.51 27.06 26.52
200 190 180 170 160 150 140 130 120 110 100 90 80 70 60 50 40 30 20 10 0
f1 (ppm)

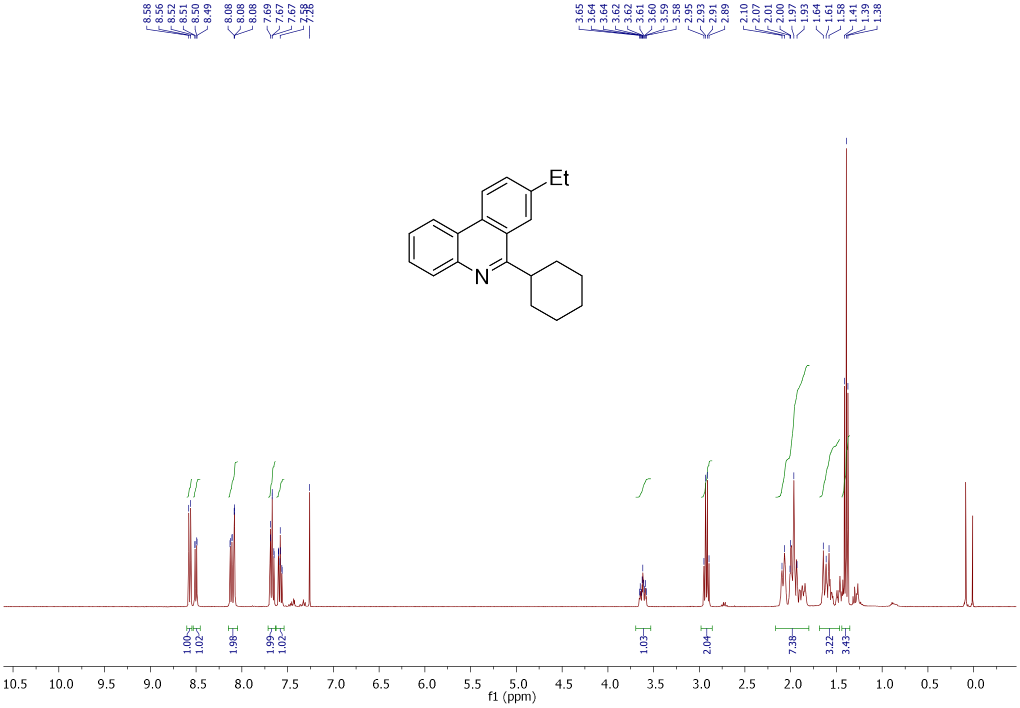
8.58
8.56
8.52
8.51
8.50
8.49
8.08
8.08
8.08
7.69
7.67
7.67
3.65
3.64
3.64
3.62
3.62
3.61
3.60
3.59
3.58
2.95
2.93
2.91
2.89
2.10
2.07
2.01
2.00
1.97
1.93
1.64
1.61
1.58
1.41
1.39
1.38
Et
N
1.00
1.02
1.98
1.99
1.02
1.03
2.04
7.38
3.22
3.43
f1 (ppm)

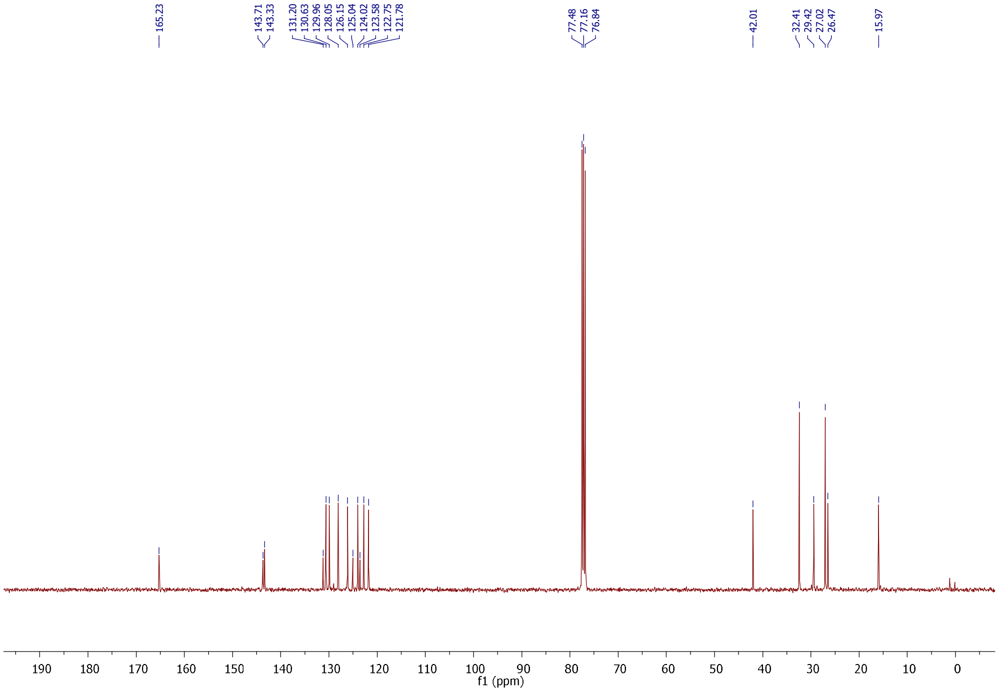
165.23
143.71
143.33
131.20
130.63
129.96
128.05
126.15
125.04
124.02
123.58
122.75
121.78
77.48
77.16
76.84
42.01
32.41
29.42
27.02
26.47
15.97
f1 (ppm)

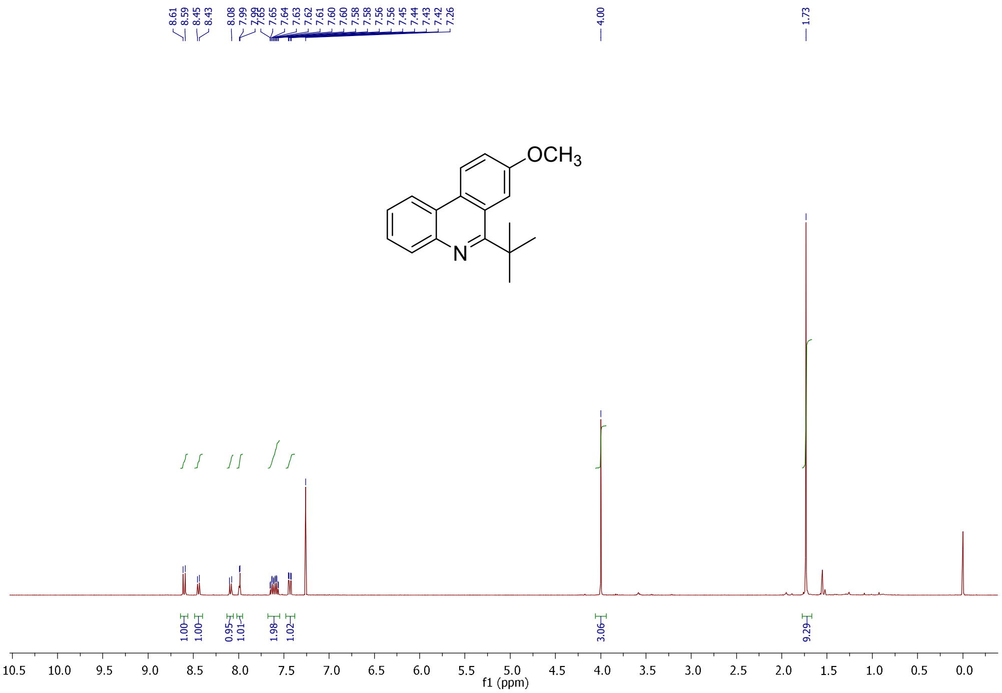
8.61
8.59
8.45
8.43
8.08
7.99
7.98
7.65
7.64
7.63
7.62
7.61
7.60
7.58
7.58
7.56
7.45
7.44
7.43
7.42
7.26
4.00
1.73
OCH3
N
1.00
1.00
0.95
1.01
1.98
1.02
3.06
9.29
10.5 10.0 9.5 9.0 8.5 8.0 7.5 7.0 6.5 6.0 5.5 5.0 4.5 4.0 3.5 3.0 2.5 2.0 1.5 1.0 0.5 0.0
f1 (ppm)

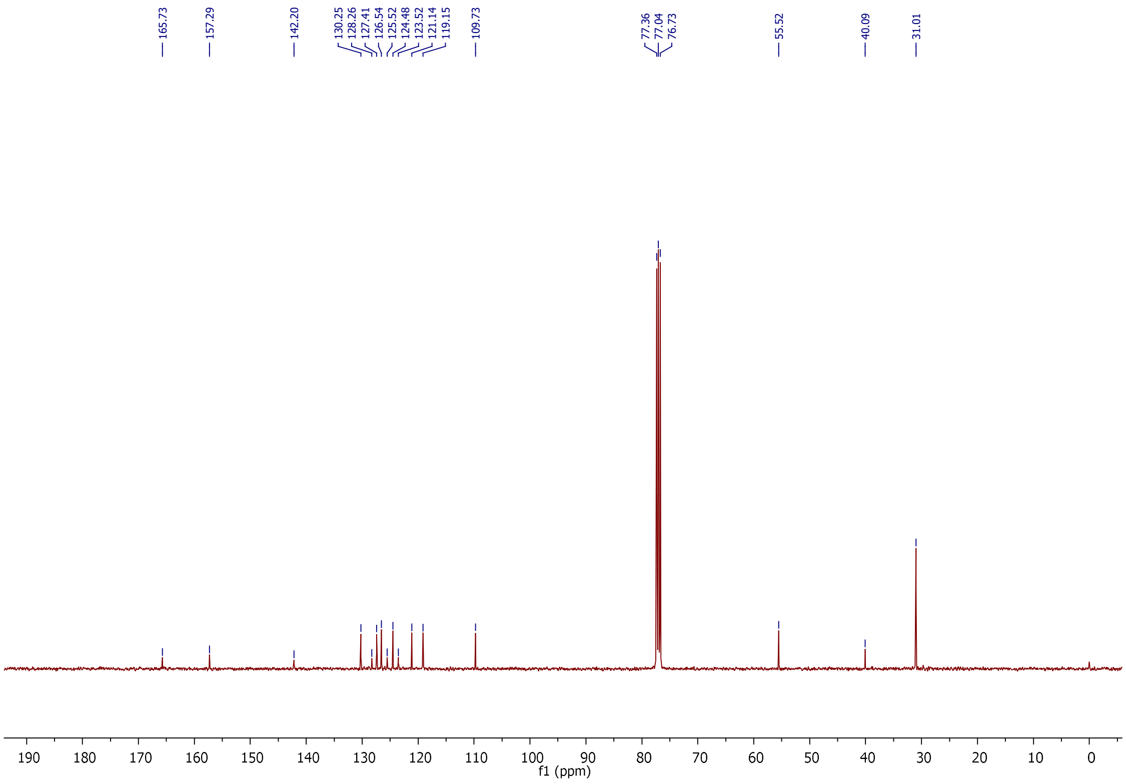
165.73
157.29
142.20
130.25
128.26
127.41
126.54
125.52
124.48
123.52
121.14
119.15
109.73
77.36
77.04
76.73
55.52
40.09
31.01
190 180 170 160 150 140 130 120 110 100 90 80 70 60 50 40 30 20 10 0
f1 (ppm)

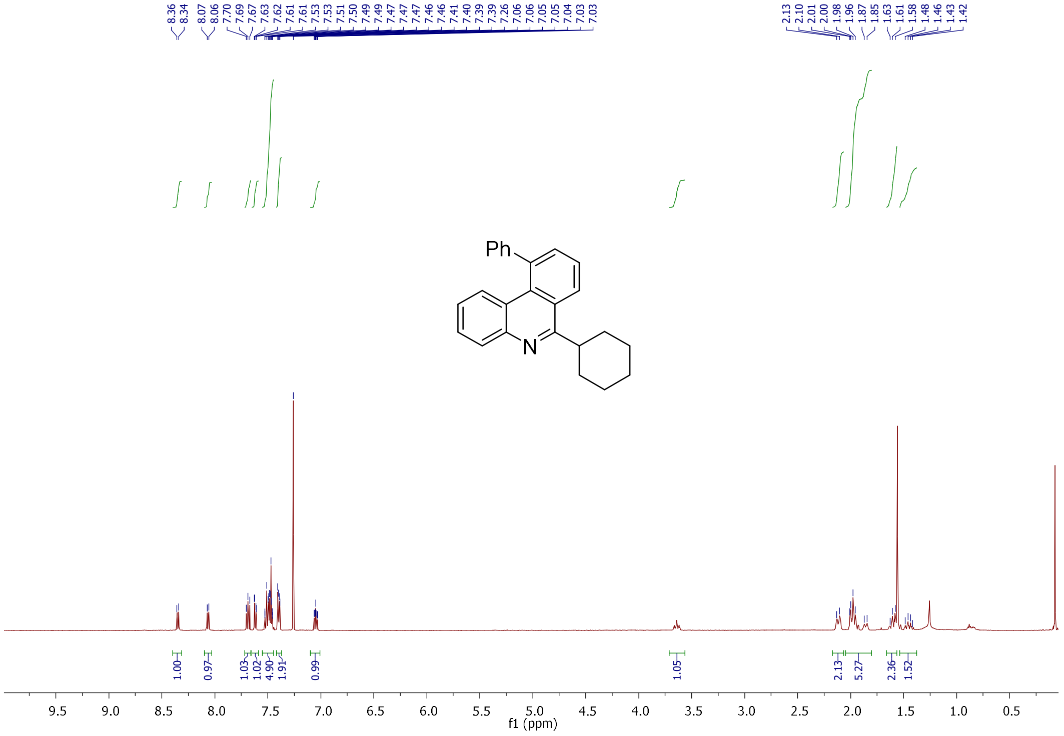
8.36
8.34
8.07
8.06
7.70
7.69
7.67
7.63
7.62
7.61
7.61
7.53
7.53
7.51
7.50
7.49
7.49
7.47
7.47
7.46
7.46
7.41
7.40
7.39
7.39
7.26
7.06
7.06
7.05
7.04
7.03
7.03
2.13
2.10
2.01
2.00
1.98
1.96
1.87
1.85
1.63
1.61
1.58
1.48
1.46
1.43
1.42
Ph
N
1.00
0.97
1.03
1.02
4.90
1.91
0.99
1.05
2.13
5.27
2.36
1.52
9.5 9.0 8.5 8.0 7.5 7.0 6.5 6.0 5.5 5.0 4.5 4.0 3.5 3.0 2.5 2.0 1.5 1.0 0.5
f1 (ppm)

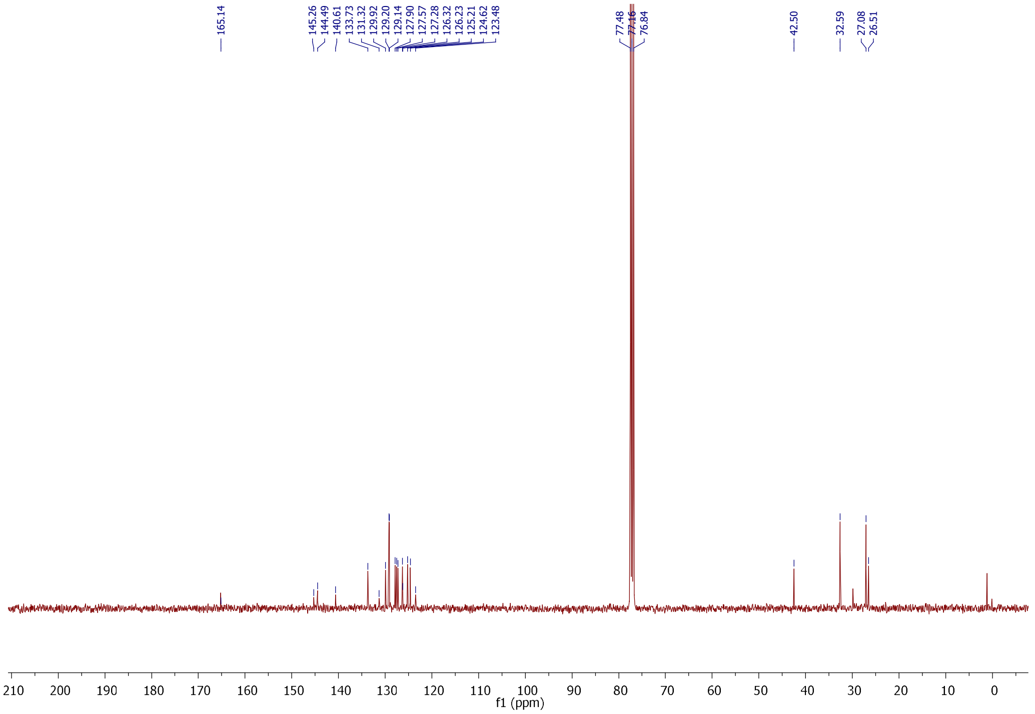
165.14
145.26
144.49
140.61
133.73
131.32
129.92
129.20
129.14
127.90
127.57
127.28
126.32
126.23
125.21
124.62
123.48
77.48
77.16
76.84
42.50
32.59
27.08
26.51
210 200 190 180 170 160 150 140 130 120 110 100 90 80 70 60 50 40 30 20 10 0
f1 (ppm)

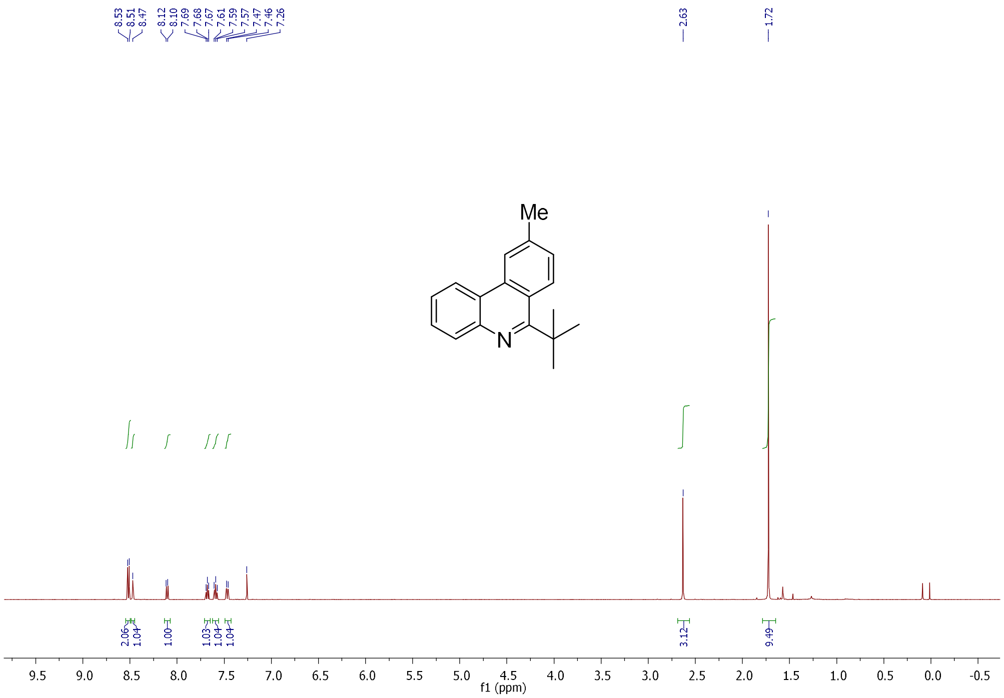

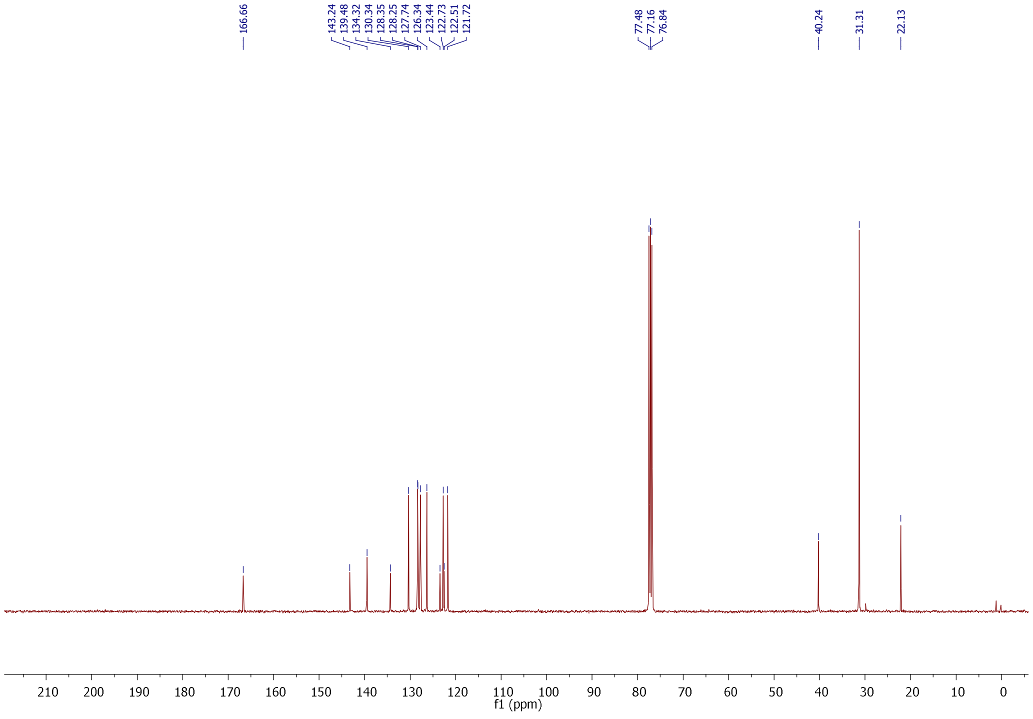

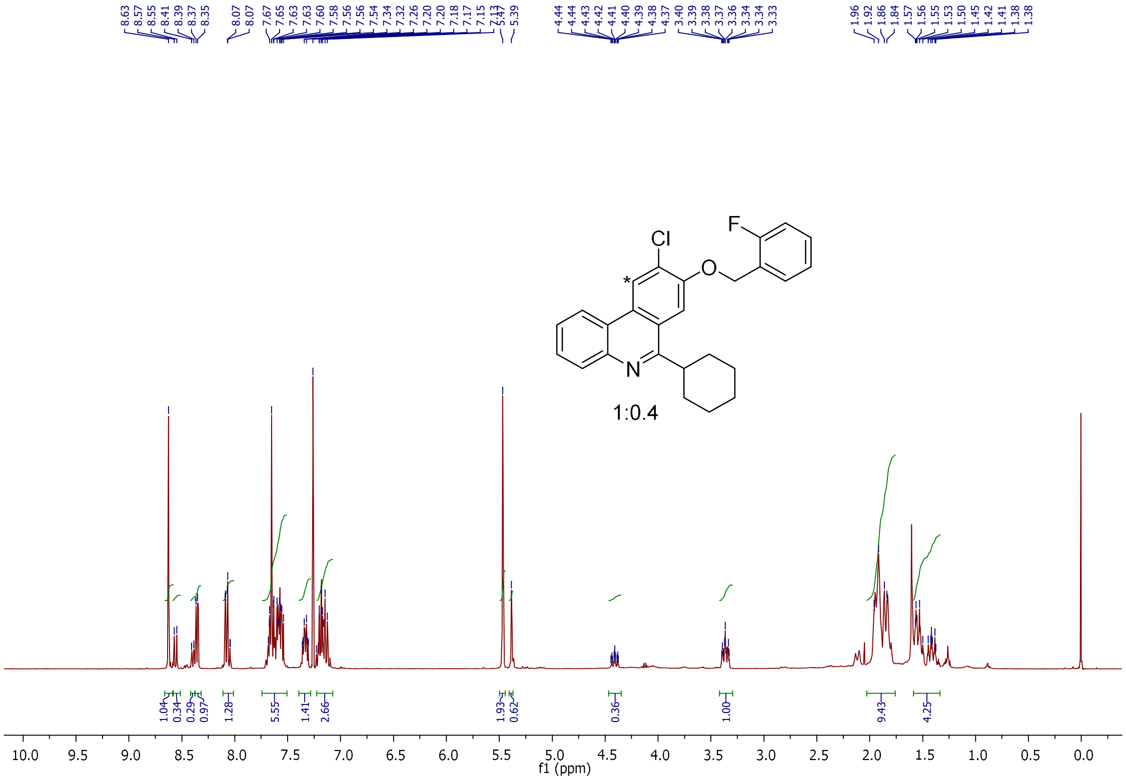

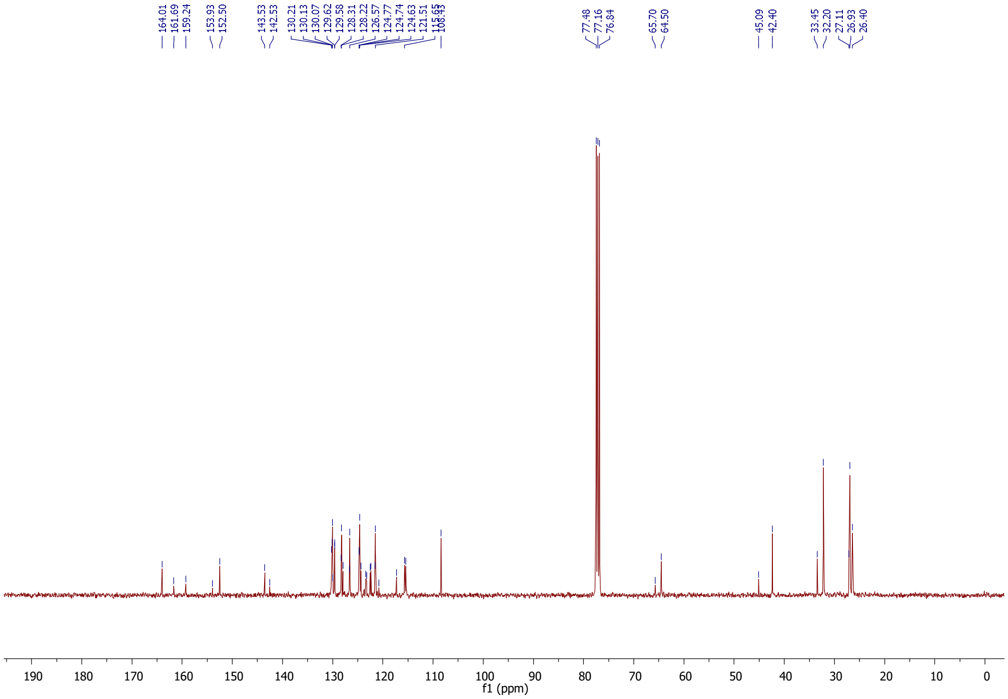

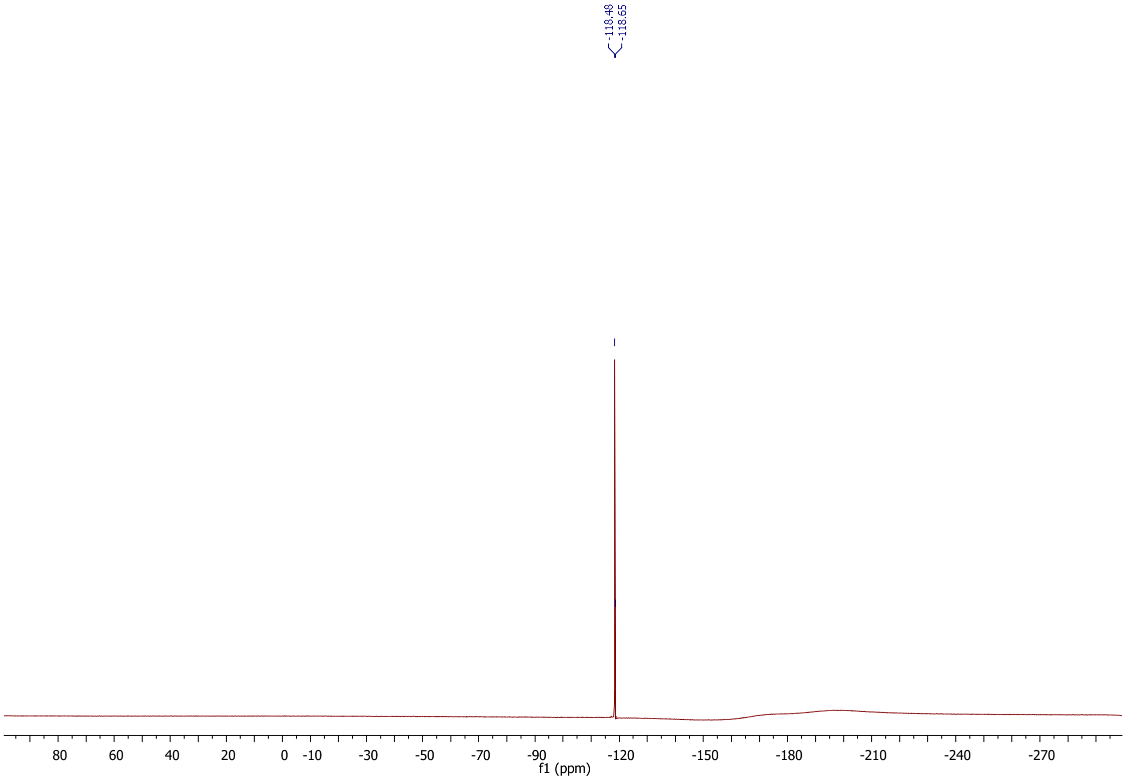
-118.48
-118.65
80
60
40
20
0
-10
-30
-50
-70
-90
-120
-150
-180
-210
-240
-270
f1 (ppm)

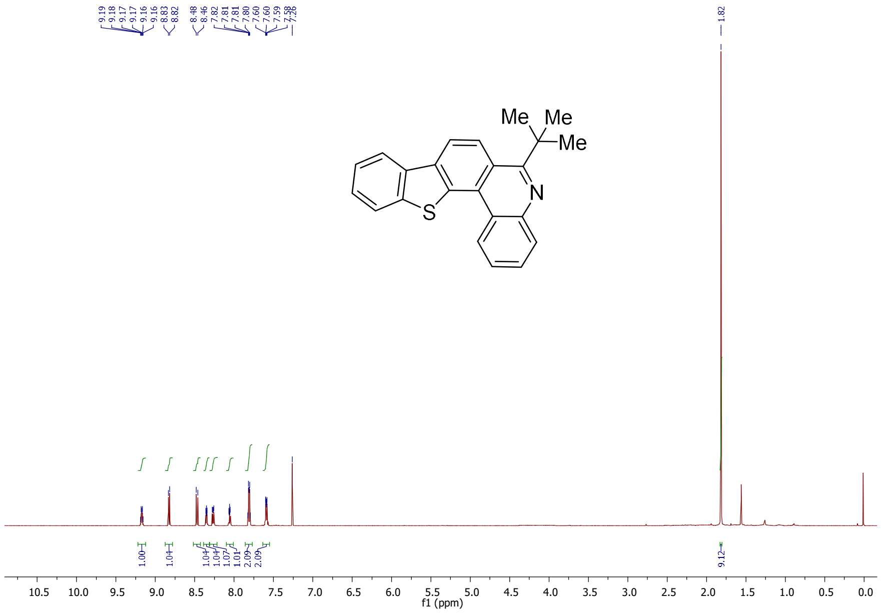

9.19
9.18
9.17
9.17
9.16
9.16
8.83
8.82
8.48
8.46
7.82
7.81
7.81
7.80
7.60
7.60
7.59
7.58
7.26
1.82
Me
Me
Me
N
S
1.00
1.04
1.04
1.04
1.07
1.01
2.09
2.09
9.12
10.5
10.0
9.5
9.0
8.5
8.0
7.5
7.0
6.5
6.0
5.5
5.0
4.5
4.0
3.5
3.0
2.5
2.0
1.5
1.0
0.5
0.0
f1 (ppm)

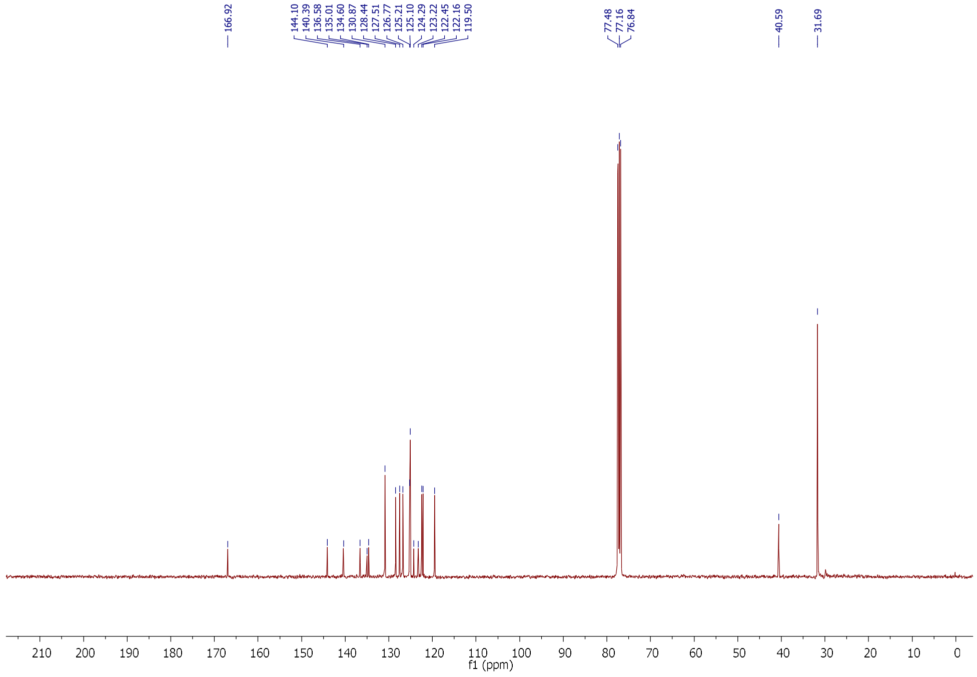

166.92
144.10
140.39
136.58
135.01
134.60
130.87
128.44
127.51
126.77
125.21
125.10
124.29
123.22
122.45
122.16
119.50
77.48
77.16
76.84
40.59
31.69
210
200
190
180
170
160
150
140
130
120
110
100
90
80
70
60
50
40
30
20
10
0
f1 (ppm)

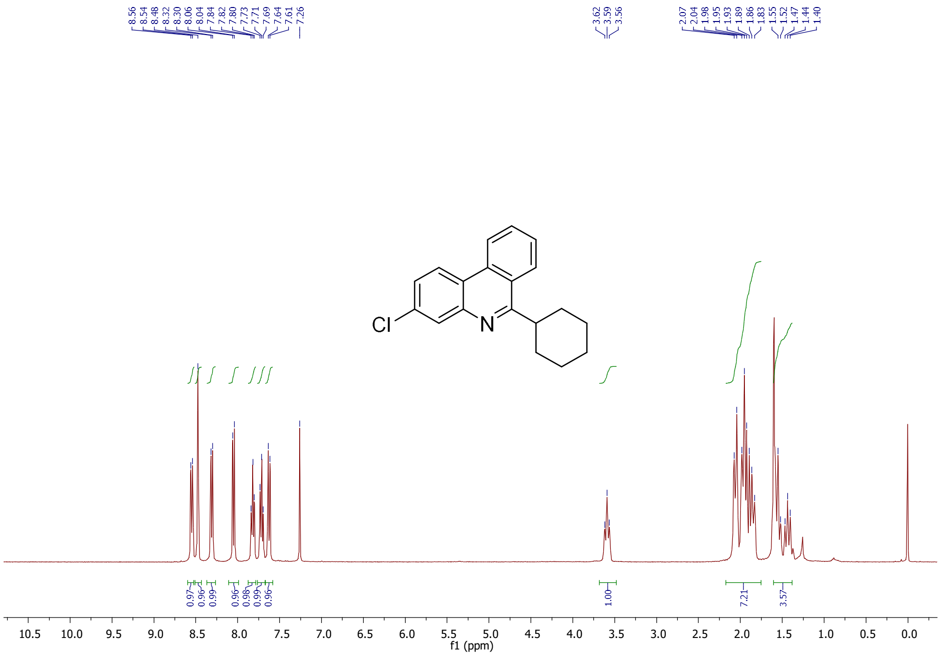

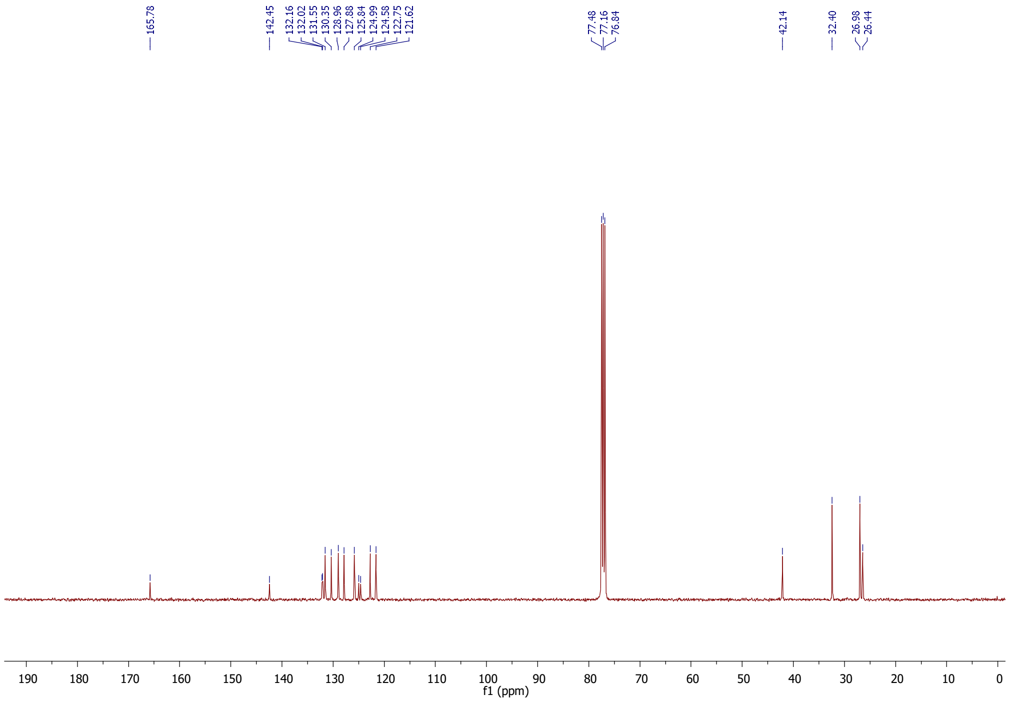

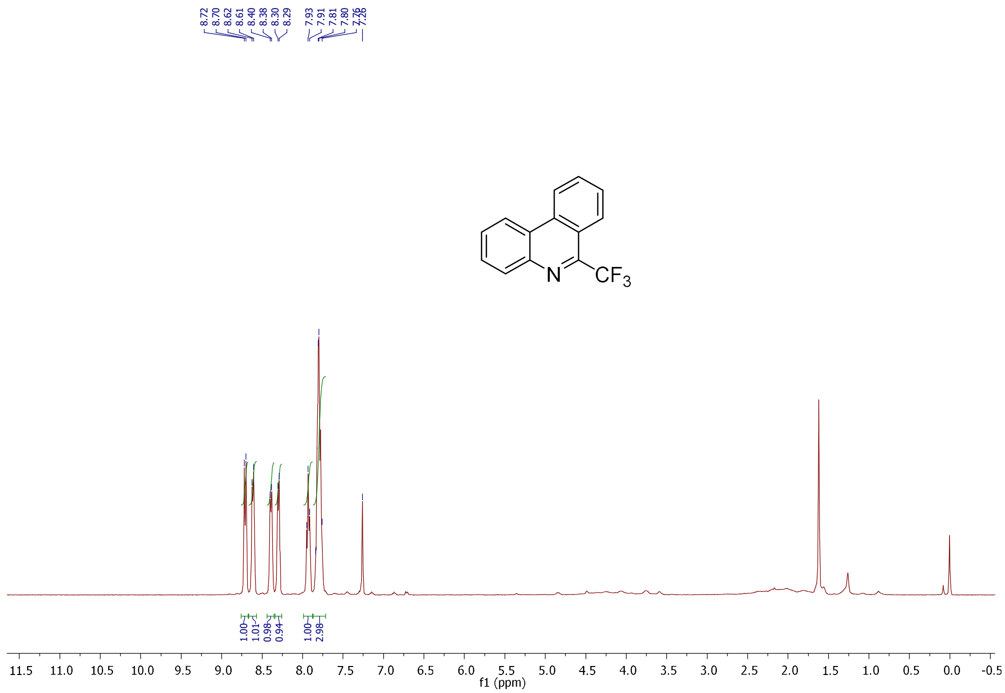

8.72
8.70
8.62
8.61
8.40
8.38
8.30
8.29
7.93
7.91
7.81
7.80
7.76
7.26
N
CF3
1.00
1.01
0.98
0.94
1.00
2.98
11.5 11.0 10.5 10.0 9.5 9.0 8.5 8.0 7.5 7.0 6.5 6.0 5.5 5.0 4.5 4.0 3.5 3.0 2.5 2.0 1.5 1.0 0.5 0.0 -0.5
f1 (ppm)

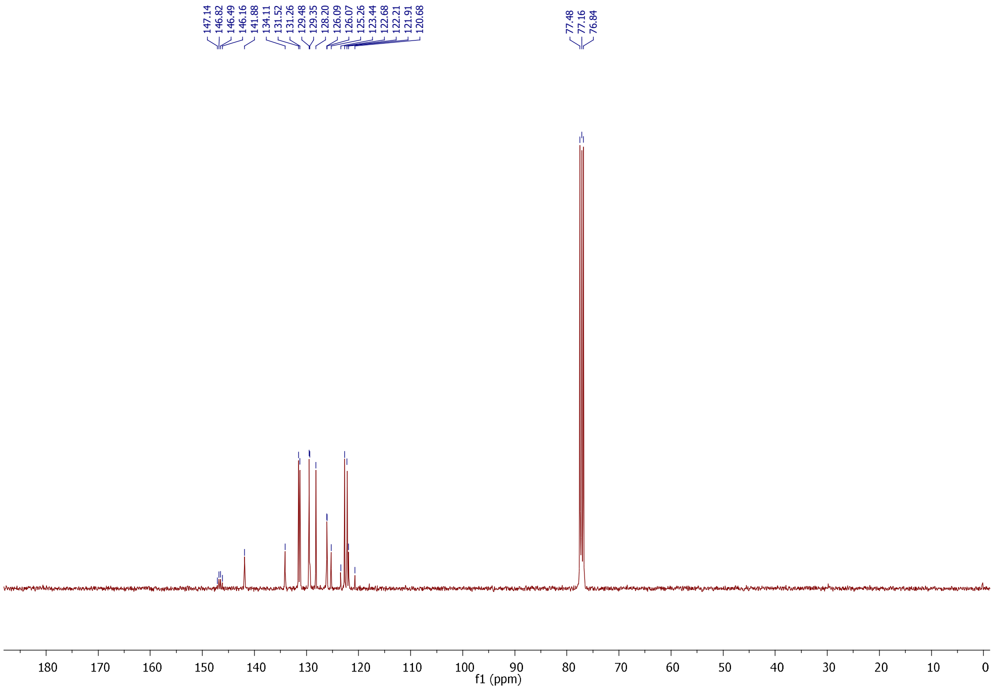

147.14
146.82
146.49
146.16
141.88
134.11
131.52
131.26
129.48
129.35
128.20
126.09
126.07
125.26
123.44
122.68
122.21
121.91
120.68
77.48
77.16
76.84
180 170 160 150 140 130 120 110 100 90 80 70 60 50 40 30 20 10 0
f1 (ppm)

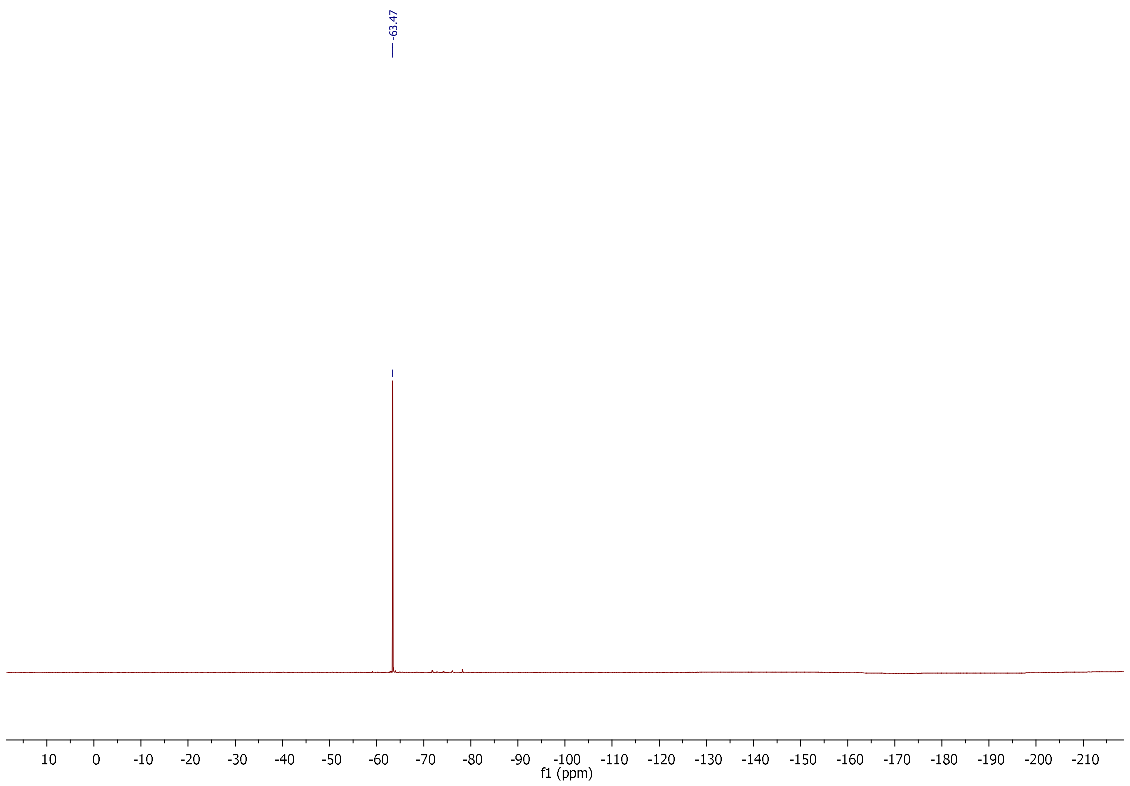
-63.47
10
0
-10
-20
-30
-40
-50
-60
-70
-80
-90
-100
-110
-120
-130
-140
-150
-160
-170
-180
-190
-200
-210
f1 (ppm)

References:

1. J. Cornella, J. T. Edwards, T. Qin, S. Kawamura, J. Wang, C.-M. Pan, R. Gianatassio, M. Schmidt, M. D. Eastgate, P. S. Baran, *J. Am. Chem. Soc*. **2016**, *138*, 2174.
2. J. Wang, B. P. Cary, P. D. Beyer, S. H. Gellman, D. J. Weix, *Angew. Chem, Int. Ed.*, **2019**, *58*, 12085
3. A. Tlahuext-Aca, R. A. Garza-Sanchez, M. Schafer, F. Glorius, *Org. Lett*. **2018**, *20*, 1546.
4. R. Mao, J. Balon, X. Hu, *Angew. Chem, Int. Ed*. **2018**, *57*, 13624.
5. W. Zhao, R. P. Wurz, J. C. Peters, G. C. Fu, *J. Am. Chem. Soc.* **2017, 139**, 12153.
6. H, Li, C. P. Breen, H. Seo, T. F. Jamison, Y. Q. Fang, M. M. Bio, *Org. Lett.* **2018**, *20*, 1338.
7. M. –C. Fu, R. Shang, B. Zhao, B. Wang, Y. Fu, *Science*, ***2019***, 363, 1429.
8. L. Huang L, A. M. Olivares, D. J. Weix, *Angew. Chem, Int. Ed*. **2017**, *56*, 11901.
9. J. Rong, L. Deng, P. Tan, C. Ni, Y. Gu, and J. Hu, *Angew. Chem, Int. Ed*. **2016**, *55*, 2743.
10. J. Li, C. A. D. Caiuby, M. W. Paixão, C.-J. Li, *Eur. J. Org. Chem*. **2018**, 2498.
11. D. Leifert, C. G. Daniliuc, and A. Studer, *Org. Lett*. **2013,** *15,* 6286.
12. W. Sha, J.-T. Yu, H. Yang,Y. Jiang, J. Cheng, *Chem. Commun.* **2014**, *50*, 9179.
13. Y.-F. Chen, J.-C. Hsieh, *Org. Lett.* **2014***, 16,* 4642.
14. W.-M. Cheng, R. Shang, M.-C. Fu, Y. Fu, *Chem. Eur. J*. **2017**, *23*, 2537.
15. S.-C. Lu, H.-S. Li, Y.-L. Gong, X.-L. Wang, F.-R. Li, F. Li, G.-Y. Duanb and S. Xu, *RSC Adv*. **2017**, *7*, 55891.
16. X.-Y. Zhang, W.-Z. Weng, H. Liang, H. Yang, B. Zhang, *Org. Lett*. **2018**, *20*, 4686.
17. C. Tang, Y. Yuan, N. Jiao, *Org. Lett*. **2015**, *17*, 2206.
18. W. –F. Tian, C.-H. Hu, K. –H. He, X.-Y. He, Y. Li, *Org. Lett*. **2019**, *21*, 6930.
19. J-C. Yang, J.-Y. Zhang, J. –J. Zhang, X.-H. Duan, L. -N. Guo, *J. Org. Chem*. **2018**, *83*, 1598.
20. Y. Jaiswal, Y. Kumar, J. Pal, R. Subramanian, A. Kumar, *Chem. Commun.* **2018**, *54*, 7207.
21. J.-C. Yang, J. –J. Zhanga, L.-N. Guo, *Org. Biomol. Chem*. **2016**,*14*, 9806.
22. B. Wang, Y. Dai, W. Tonga, H. Gong, *Org. Biomol. Chem.* **2015**,*13*, 11418.
23. W. Sha, J.-T. Yu, H. Yang, Y. Jiang, J. Cheng, *Chem. Commun.* **2014**, *50*, 9179.
24. S. Liu, W. Pan, S. Wu, X. Bu, S. Xin, J. Yu , H. Xu, X. Yang, *Green Chem*. **2019**, *21*, 2905.
25. S. Rohe, T. McCallum, A. O. Morris, L. Barriault, *J. Org. Chem*. **2018**, *83*, 10015.
26. W.-Y. Wang, X. Feng, B.-L. Hu, C.-L. Deng, X.-G. Zhang, *J. Org. Chem*. **2013**, *78*, 6025.
